# A new Early Cretaceous lizard in Myanmar amber with exceptionally preserved integument

**DOI:** 10.1038/s41598-022-05735-5

**Published:** 2022-01-31

**Authors:** Andrej Čerňanský, Edward L. Stanley, Juan D. Daza, Arnau Bolet, J. Salvador Arias, Aaron M. Bauer, Marta Vidal-García, Joseph J. Bevitt, Adolf M. Peretti, Nyi Nyi Aung, Susan E. Evans

**Affiliations:** 1 Department of Ecology, Laboratory of Evolutionary Biology, Faculty of Natural Sciences, Comenius University in Bratislava, Mlynská dolina, 84215 Bratislava, Slovakia; 2 Department of Natural History, Florida Museum of Natural History, Gainesville, FL USA; 3 Department of Biological Sciences, Sam Houston State University, Huntsville, TX USA; 4 Institut Català de Paleontologia Miquel Crusafont, Universitat Autònoma de Barcelona, Barcelona, Spain; 5 School of Earth Sciences, University of Bristol, Bristol, UK; 6 Unidad Ejecutora Lillo (CONICET, Fundación Miguel Lillo), San Miguel de Tucumán, Argentina; 7 Department of Biology and Center for Biodiversity and Ecosystem Stewardship, Villanova University, Villanova, PA USA; 8 Department of Cell Biology and Anatomy, University of Calgary, Calgary, Canada; 9 Australian Centre for Neutron Scattering, Australian Nuclear Science and Technology Organisation, Sydney, Australia; 10 GRS Gemresearch Swisslab AG, Baumschulweg 13, 6045 Meggen, Switzerland; 11 Peretti Museum Foundation, Baumschulweg 13, 6045 Meggen, Switzerland; 12 Myanmar Geosciences Society, c/o Department of Geology, University of Yangon, 11041 Yangon, Myanmar; 13 Department of Cell and Developmental Biology, University College London, London, UK

**Keywords:** Palaeontology, Herpetology

## Abstract

We here report on a well-preserved juvenile lizard specimen in Albian amber (ca. 110 mya) from the Hkamti site (Myanmar). This new taxon is represented by an articulated skull and the anterior portion of the trunk, including the pectoral girdle and forelimbs. The scleral ossicles and eyelid are also visible, and the specimen exhibits pristine detail of the integument (of both head and body). In a combined molecular and morphological analysis, it was consistently recovered as a scincoid lizard (Scinciformata), as sister to *Tepexisaurus* + Xantusiidae. However, the phylogenetic position of the new taxon should be interpreted with caution as the holotype is an immature individual. We explored the possibility of miscoding ontogenetically variable characters by running alternative analyses in which these characters were scored as missing data for our taxon. With the exception of one tree, in which it was sister to Amphisbaenia, the specimen was recovered as a Pan-xantusiid. Moreover, we cannot rule out the possibility that it represents a separate lineage of uncertain phylogenetic position, as it is the case for many Jurassic and Cretaceous taxa. Nonetheless, this fossil offers a rare opportunity to glimpse the external appearance of one group of lizards during the Early Cretaceous.

## Introduction

Lizards (including amphisbaenians) and snakes, form Squamata, a clade of primarily terrestrial reptiles. They show a diversity of foraging modes, antipredation strategies, and Baupläne. Such adaptations help them to be highly successful in an extended range of environments, and they are the largest group of non-avian reptiles^1–3^. Squamates have a history estimated to date back over 200 million years^4–7^. Their early Mesozoic record remains limited, but their Cretaceous fossil record has been improving, especially in the last few decades. Nonetheless, this Cretaceous record is heavily biased toward northern continents; far less is known of the Mesozoic squamate record in Gondwana, leaving many aspects of the evolutionary history and palaeobiogeography of lizards uncertain.

The amber mines of Katchin State, northern Myanmar form a series of deposits dated from ~ 110 Ma (Hkamti amber; i.e., the “new mine”) to ~ 72 Ma (Tilin site, see Xing and Qiu^8^; Nyunt et al.^9^), whereas specimens from elsewhere in the Hukawng valley (for the map of the localities, see Supplementary Data 1) are dated to 99 Ma^10^. These amber deposits have yielded a significant number of mostly early Cenomanian squamates as inclusions, including some exquisitely preserved lizards^11–17^ and snakes^18^. Elsewhere in the world, a majority of Albian and Cenomanian terrestrial lizard fossils are represented by disarticulated and isolated elements^19–25^, whereas Myanmar amber is famous for the extraordinary preservation of articulated fossils with preserved integument, although many of these are limited to isolated limbs and/or tails^12^. Therefore, the amber deposits in Myanmar provide a unique window into the mid-Cretaceous world. The Burma Terrane has been reconstructed as part of a Trans-Tethyan island arc at the time of amber deposition, at least in several models^26,27^, with the amber biota representing an endemic island fauna, possibly of Gondwanan derivation^26^. The best represented group is Gekkota, which today contains over 2000 extant species of geckos and pygopods^3^, and is distributed worldwide in warm temperate to tropical areas^28,29^. However, the skeletons of these lizards are often delicate and the global gekkotan fossil record is relatively poor, making the Myanmar amber deposits particularly significant for this clade^11,12^. Other lineages—including iguanians—may also be represented from these deposits^12,16,17^.

In this paper, we report on a well-preserved juvenile specimen from the Albian amber of the Hkamti site (ca. 110 mya). The specimen is represented by the articulated skull and the anterior portion of the trunk, including the pectoral girdle and anterior limbs, and is characterized by pristine detail of the integument. In this paper we describe the specimen in detail using high-resolution X-ray microcomputed tomography (synchrotron data), and discuss the possible affinities of the new taxon.

## Systematic Palaeontology

Squamata Oppel, 1811^30^.

Scinciformata Vidal and Hedges, 2005^31^.

Scincoidea Oppel, 1811^30^.

? Pan-Xantusiidae Gauthier et al., 2012^2^.

*Retinosaurus* gen. nov.

### Etymology

The Greek word "Retine" which is the general term for all resin liquids exuded from tree trunks (lithified as amber) and *saurus* meaning lizard.

### Diagnosis

As for *Retinosaurus hkamtiensis*, the only known species.

*Retinosaurus hkamtiensis* gen. et sp. nov.

LSID for this species: zoobank.org:pub:87548612-CECB-4631–9076-07A65813A7B9.

### Etymology

*hkamtiensis;* after the locality Hkamti.

### Holotype

**Figure 1:**
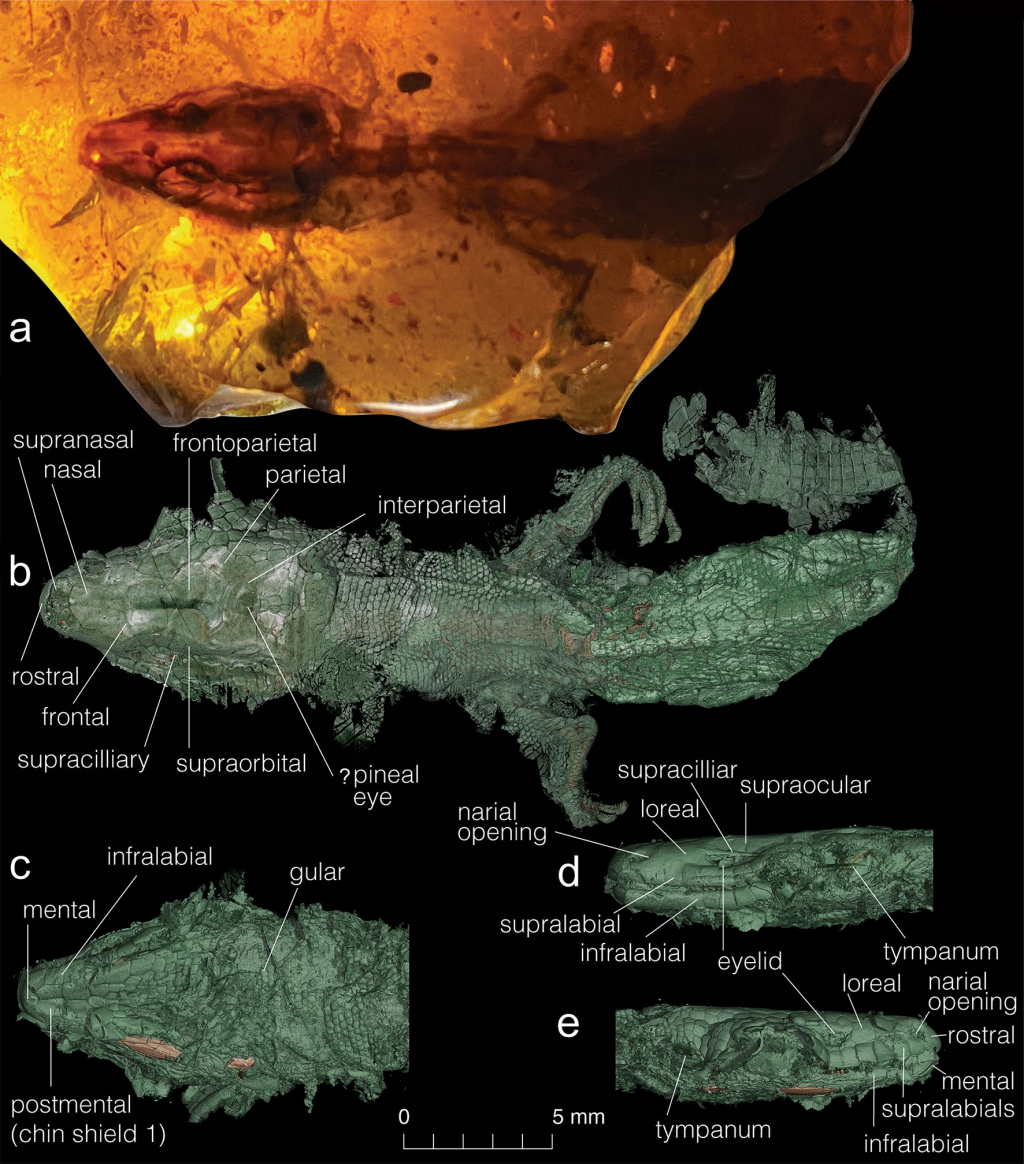
General view of *Retinosaurus hkamtiensis* (GRS 29689). (**a**) Photograph of the specimen within the amber resin in dorsal view. (**b**–**d**) HRCT rendering of the integument surface. Note that the integument is not visible in the photograph, as it may be preserved as a translucent layer. (**b**) dorsal view of the body, (**c**) ventral side of the head, (**d**) left lateral and (**e**) right lateral views of the head. Greenish colour indicates the skin, light brown indicates the bones preserved inside.

**Figure 2:**
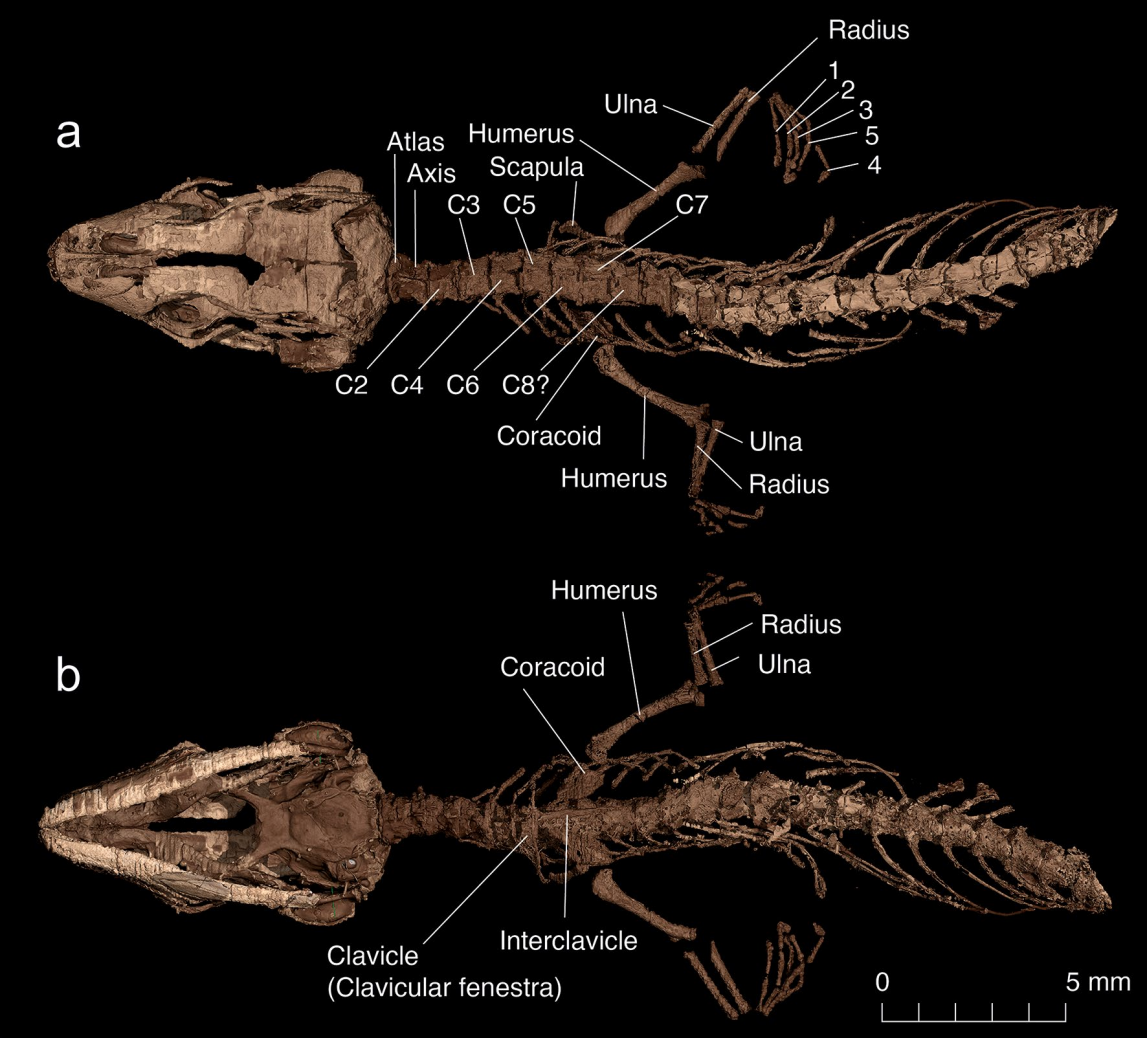
(**a**) Dorsal and (**b**) ventral views of *Retinosaurus hkamtiensis* (GRS 29689). The images show the skeleton of the specimen after removing the calcified material within the abdomen.

**Figure 3:**
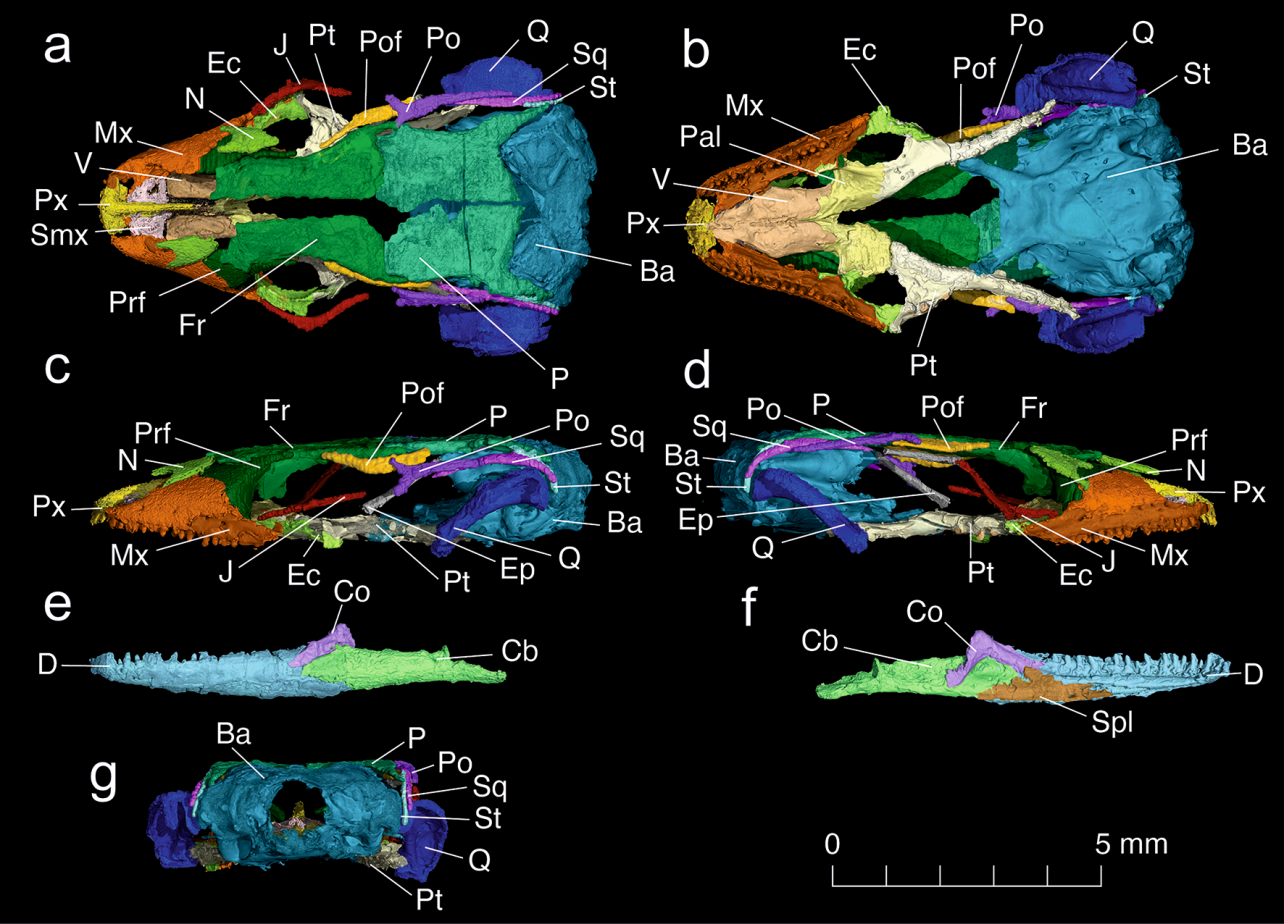
Skull of *Retinosaurus hkamtiensis* (GRS 29689) based on X-ray microcomputed tomography (synchrotron data). Individual bones segmented using VGSTUDIO MAX (Volume Graphics GmbH), rendering each bone as a volume. Cranium in dorsal (**a**), ventral (**b**), left lateral (**c**), right lateral (**d**), and posterior (**g**) views, Jaw in lateral (**e**) and medial (**f**) views. *Ba* braincase, *Cb* compound bone, *Co* coronoid, *D* dentary, *Ec* ectopterygoid, *Ep* epipterygoid, *Fr* frontal, *J* jugal, *Mx* maxilla, *N* nasal, *P* parietal, *Pal* palatine, *Po* postorbital, *Pof* postfrontal, *Prf* prefrontal, *Pt* pterygoid, *Px* premaxilla, *Spl* splenial, *Q* quadrate, *Smx* septomaxilla, *Sq* squamosal, *St* supratemporal, *V* vomer.

**Figure 4:**
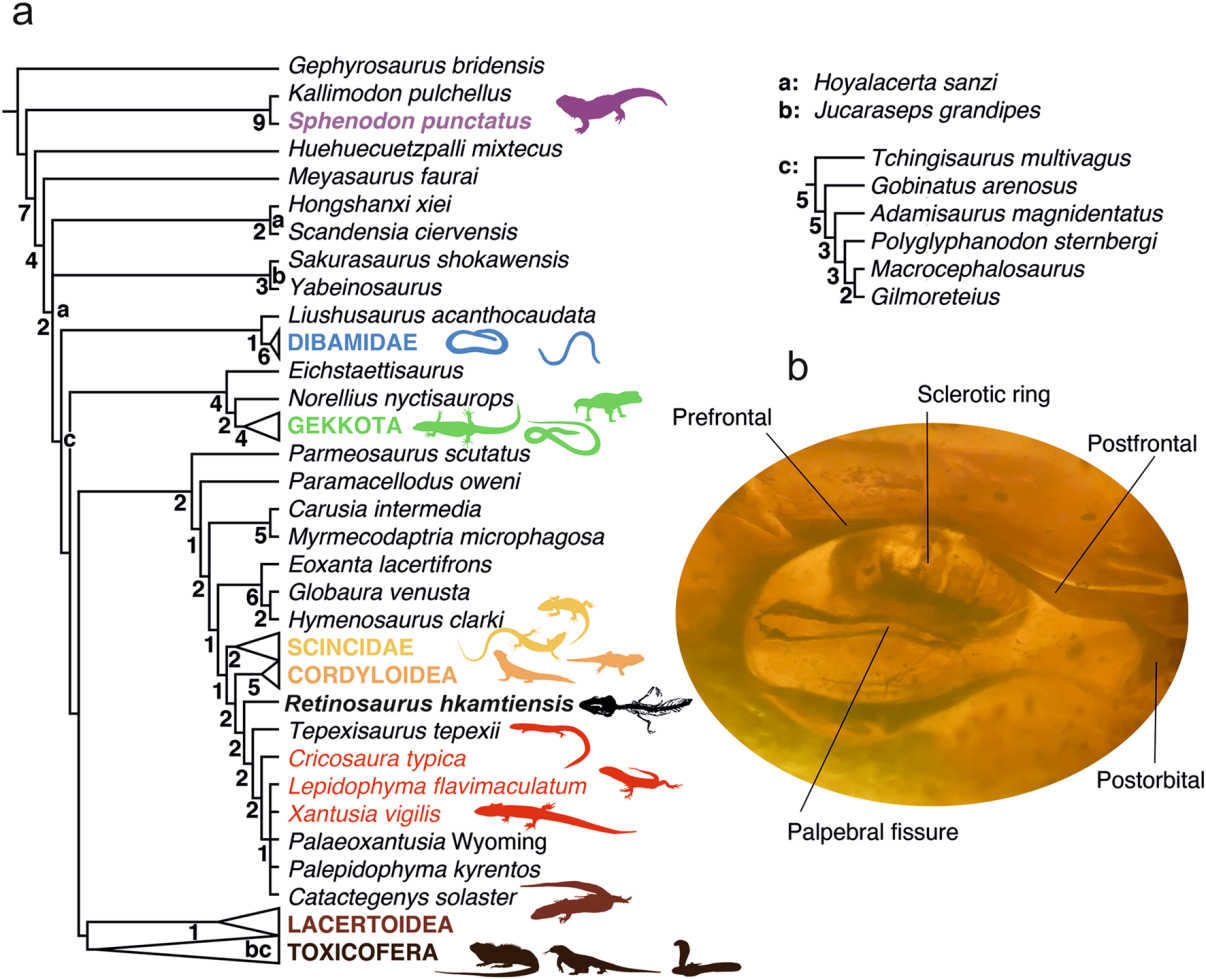
(**a**) Phylogenetic position of *Retinosaurus hkamtiensis* (GRS 29689) using a combined data set of morphological and molecular data, including all characters (i.e. not coding ontogenetically variable characters of *Retinosaurus* as ?), and treating characters as additive following Gauthier et al.^2^. Terminals and groups numbered a–c were identified as wildcard taxa, therefore the strict consensus was calculated without them, and their alternative positions indicated in the tree. Using the combined data set, *R. hkamtiensis* was recovered as a stem-xantusiid. Numbers below nodes indicate Bremer support values. Letter at nodes indicate alternative positions of wildcard taxa (right). Nodes with no support values were collapsed when wildcards were included in the consensus. (**b**) Inset left side of *R. hkamtiensis* showing the preservation of the eyelids and circumorbital bones.

GRS 29689, an amber-inclusion with a well-preserved skull, including the mandible, part of the hyoid (ceratobranchial 1), and a partial postcranial skeleton, as well as well-preserved skin tissues (Figs. 1, 2, 3, 4b and Supplementary Figs. 1–4). The specimen is housed in the Peretti Museum Foundation (PMF), Gem Research Swiss Laboratory (GRS) in Meggen Switzerland. The PMF fulfills all requirements to hold a legal collection under Swiss law and provides access to all bona fide researchers. Several coleopterans were trapped with the specimen (see Supplementary Data 1).

### Type locality, horizon and material

The specimen was recovered from the Hkamti District at Patabum (Sagaing Region), in close proximity of the Jade mines, 100 km to the southwest of the Hukawng Basin in the northern Myanmar Central Basin^32^.

### Age

Amber from this mine at the Hkamti District has been dated to the Albian, ca. 110 million years ago (Ma), using zircon U–Pb isotopes^8^.

### Diagnosis

A lizard differentiated from other named Myanmar fossil squamates in a combination of features including short jaws, absence of a lacrimal, and procoelous vertebrae (contra *Oculudentavis khaungraee* Xing et al.^33^, *O. naga* Bolet et al.^17^), and fully limbed with classical lizard body proportions (contra limb-attenuated as in *Barlochersaurus winhtini* Daza et al*.*^13^). *Protodraco monocoli* Wagner et al.^16^ is an isolated limb, but differs from *Retinosaurus* in having finer scalation. Allowing for immaturity, *Retinosaurus* also differs from other roughly contemporaneous fossil squamates known from Europe, Asia, and the Americas in the following combination of characters: depressed (box-shaped) skull; nasal process of unpaired premaxilla long, almost reaching frontals; anterior width of nasals exceeds nasofrontal joint width; elongate frontal plate only weakly emarginated by orbits (contra *Eichstaettisaurus* Kuhn^34^, *Liushusaurus* Evans and Wang^35^, *Meyasaurus* Vidal^36^*, Huehuecuetzpalli* Reynoso^37^); anteriorly well-developed subolfactory processes that extend toward the ventral midline; interdigitated fronto-parietal suture (as in *Yabeinosaurus* Endo and Shikama^38^*, Sakurasaurus* Evans and Manabe^39^, but contra *Huehuecuetzpalli*, *Tepexisaurus* and extant xantusiids), with parietal tabs underlying frontals; paired parietals lacking ventral fossa for supraoccipital processus ascendens (contra *Yabeinosaurus, Sakurasaurus, Kuroyuriella* Evans and Matsumoto^40^*, Hoyalacerta* Evans and Barbadillo^41^*, Dorsetisaurus* Hoffstetter^42^, *Purbicella* Evans et al*.*^43^, *Jucaraseps* Bolet and Evans^44^, *Huehuecuetzpalli*, paramacellodids, polyglyphanodontians); lacrimal bone absent (contra *Purbicella*); palatal dentition absent (contra e.g., *Dalinghosaurus* Ji^45^, *Yabeinosaurus, Purbicella*); ectopterygoid with hooked posterior process that is laterally exposed (as *Tepexisaurus* Reynoso and Callison^46^); ectopterygoid contacts palatine to exclude the maxilla from the lateral margin of the suborbital fenestra; large, deeply recessed lateral opening of the recessus scalae tympani; jaw joint lies well anterior to level of occipital condyle (contra e.g., *Huehuecuetzpalli*, *Tepexisaurus*); open Meckel’s groove (contra derived state in extanct xantusiids); retention of a separate splenial and angular (contra derived state in extant xantusiids); homodont pleurodont dentition of moderately pointed and unicuspid tooth crowns (contra bicuspid as in *Meyasaurus, Hakuseps* Evans and Matsumoto^40^, *Pedrerasaurus* Bolet and Evans^47^; multicuspid in *Asagaolacerta* Evans and Matusmoto^40^ and many polyglyphanodontians including *Kuwajimalla* Evans and Manabe^48^; robust and striated, as in *Saurillodon* Estes^49^; truncated with anteroposteriorly directed apical groove in contogeniids, or rounded in *Gueragama* Simões et al.^50^); splenial short, not reaching mid-point of dentary; long straight retroarticular process (e.g., contra *Meyasaurus, Tepexisaurus, Huehuecuetzpalli*); zygapophysial facet between atlas and axis; first and second intercentrum small and not in contact with each other; cruciform interclavicle with long anterior process (contra rhomboid, as in *Scandensia* Evans and Barbadillo^51^*,* or T-shaped in *Huehuecuetzpalli*); short robust ungual phalanges with a terminal hook (contra slender and elongated in e.g., *Scandensia*); phalangeal formula of manus 2:3:3:4:3, with the two intermediate phalanges on digit 4 unusually short; cephalic scales have a regular dorsal scutellation pattern of alternating single and paired scales from front to back on the skull roof; four large scales and several tiny scales covering the eyelid; and horizontal palpebral fissure present.

#### Specimen description

The specimen preserves the skull, anterior vertebral column, forelimbs and pectoral girdle of a small lizard, as well as the skin and scales covering the body (Fig. 1) and skull. Surprisingly, the trachea and bronchi are also preserved (see Supplementary Data 1). The specimen is small (estimated Snout-Pelvis Length [SPL] is around 35 mm), and all indications are that this was a juvenile animal. The skull roof is incompletely ossified with a large fronto-parietal fontanelle (Figs. 2, 3); the sutures are not tightly connected (as shown by disarticulation and displacement of several skull bones); the vertebral neural arches are not fused in the dorsal midline; the scapula and coracoid are not co-ossified; the epiphyses of the forelimb bones are not ossified; and the carpals and phalanges appear weakly ossified. The immaturity of the specimen is an important consideration with respect to the description (for a detailed description of each skeletal element and illustrations of the virtually isolated braincase, inner ear, and head scaling, see Supplementary Data 1), as bone shapes and fusions may alter the appearance of the adult skull. On the whole, the skull is box-shaped—depressed and anteroposteriorly somewhat elongate. It is widest in the region of the parietals and then gradually narrows into a rounded anterior tip. Its maximum width (at the level of the quadrates) is 5.8 mm, whereas its anteroposterior length (from the tip of the snout to the occipital condyle) along the midline is 9.5 mm. The pre-orbital region is short, whereas the post-orbital portion is extended posteriorly. There is a small, narrow supratemporal fenestra, and an elongate infratemporal fenestra (Fig. 3). The jaw joint lies well anterior to the occipital condyle, giving the quadrate a strongly oblique orientation. Although there is some postmortem compression, based on the position of other bones this is unlikely to fully account for the recumbent quadrate position. The specimen preserves parts of the anterior vertebral column, pectoral girdle, and forelimb. A total of 23 procoelous presacral vertebrae are preserved, with ten anterior vertebrae clearly visible (of which 7–9 may be cervicals), whereas a further 13 vertebrae and their ribs are enclosed in a calcite sheath that partially obscures their structure.

Integumentary structures are unusually well preserved in the fossil (Fig. 1). The cephalic scales are enlarged and well preserved and follow a regular dorsal scutellation pattern of alternating single and paired scales from front to back of the skull roof. Smaller scales ranging in shape from rectangular to hexagonal cover the dorsal surface of the body, but those on the ventral side of the head are larger and rectangular. The left side preserves the eyelid and scleral ossicles which form a sclerotic ring (inset, Fig. 4). The eye has rectangular scleral ossicles, seven of which are visible in dorsal view, and assuming that the ventral ones are similar in size and spacing to the dorsal ones, the eye is estimated to have had at least 14 ossicles in total. The eyelids of the left eye are also preserved, defining a horizontal palpebral fissure, and the eye was clearly not covered by a brille or spectacle (inset, Fig. 4).

### Phylogenetic placement of GRS 29689

The specimen is fairly complete. In all analyses (see Supplementary Data 1 for details of phylogenetic analyses), the strict consensus of all the trees recovered using the combined dataset (i.e. the morphological data matrix with a molecular partition, see “Methods”) yielded a highly unresolved tree, partly due to the unstable position of the Early Cretaceous Spanish (Las Hoyas) taxa *Hoyalacerta sanzi*^41^ and *Jucaraceps grandipes*^44^, and of the Cretaceous Polyglyphanodontia^2^. To increase the resolution of the tree, another strict consensus tree was calculated without the unstable taxa, and their alternative positions are indicated on the simplified consensus (Fig. 4).

In the combined evidence analysis, where we scored all possible morphological characters, and used the character ordering proposed by Gauthier et al*.*^2^ (see “Methods”), *Retinosaurus hkamtiensis* was consistently recovered as a scincoid lizard. *Retinosaurus* was placed as the sister taxon to *Tepexisaurus* (Early Cretaceous, Mexico^46^) + Xantusiidae. Relationships within Xantusiidae were poorly resolved. *Cricosaura* was recovered as sister to an unresolved clade formed by *Xantusia* and *Lepidophyma*, the extinct *Palaeoxantusia* (Eocene, North America^52^), *Palepidophyma* (Eocene, North America^53^), and *Catactegenys* (Late Cretaceous, North America^54^). The sister group relationship between *R. hkamtiensis* and the clade formed by *Tepexisaurus* and xantusiids (including *Catactegenys*) was supported by 12 characters (present unambiguously in all trees), namely: character 7: ethmoideal foramina exit via external naris (reversal to 0 state); character 24: nasals not in ventral contact beneath premaxillary internasal process except near the apex (1); character 137: lacrimal absent; character 141: lacrimal duct enclosed in the prefrontal except ventrally; character 155: posterior process of jugal absent; character 220: vomeronasal nerve exit dorsal to vomer (reversal to 0 state); character 308: crista prootica extends onto basipterygoid process forming open or closed bony canal; character 324: dorsum sellae shallow and poorly differentiated with, at most, shallow fossa and low crista sellaris; character 334: distal end of basipterygoid process not expanded (reversal to 0 state); character 410: retroarticular process breadth relative to mandibular condyle narrow (reversal to 0 state); character 641: fleshy eyelids absent (i.g., eyelids are thin).

The holotype specimen of *Retinosaurus* is clearly immature. We explored the possibility of miscoding ontogenetically variable characters, by running alternative analyses scoring these characters as missing data (?) for *Retinosaurus* (see Table S1). In the analyses using all characters unordered and those of *Retinosaurus* coded as preserved (i.e. not scoring ontogenetically variable characters as unknown for *R. hkamtiensis*; see “Methods”), *R. hkamtiensis* was recovered as sister to amphisbaenians in one tree. All other analyses recovered *Retinosaurus* as a Pan-xantusiid (i.e. crown Xantusiidae + stem) as did the total evidence approach (see Table S1).

## Discussion

### *Retinosaurus* and Pan-xantusiids

The results of the main phylogenetic analysis outlined above placed *Retinosaurus* on the xantusiid stem. *Retinosaurus* resembles crown xantusiids in some characters of body scutellation (see Gauthier et al*.*^55^; Supplementary Data 1), and in a combination of a few skull features, although none is unique to Xantusiidae (see “Phylogenetic placement of GRS 29689”).

There are also many differences between *Retinosaurus* and crown xantusiids, including the following character states present in *Retinosaurus*: (1) open Meckel’s groove; (2) retention of a separate splenial and angular; (3) high tooth count; (4) anterior width of nasals exceeds nasofrontal joint width; (5) absence of ventral (epipterygoid) process of the parietal; (6) parietal with relatively long (not reduced) supratemporal processes; (7) frontoparietal suture moderately interdigitated; (8) supratemporal longer than squamosal-parietal contact; (9) postfrontal and postorbital separate; (10) absence of quadrate accessory process arising from anteromedial edge of quadrate head; (11) vomers unfused; (12) vomer extends backwards beyond anteriormost contact of palatine with maxilla; and (13) absence of a brille. States observed in *Retinosaurus* for some of these characters (e.g., 2, 5, 9, 10, 11) may, however, vary ontogenetically and are thus problematic because of the assumed juvenile status of the specimen. Among squamates, the combination of large cephalic scales, square to tubercular scales on the dorsum, and large rectangular ventral scales forming rows on the belly are widespread among Scincoidea and Lacertoidea—two clades that Camp^56^ and many subsequent morphological studies^2,41,51,57,58^ assigned to the infraorder Scincomorpha. However, all molecular and combined evidence analyses refute this grouping^5,7,59^ and most herpetologists now accept that scincoids and lacertoids are not closely related. The similarity between the pholidosis of *Retinosaurus* (see Supplementary Data 1) and that of Scincoidea and Lacertoidea is mirrored in the two alternative positions in our phylogenetic analyses. Although *Retinosaurus* was recovered consistently as a stem-xantusiid, it is important to note the presence of eyelids, which in the crown group are fused into a brille. If *Retinosaurus* is a stem-xantusiid, it would imply that the appearance of large cephalic scutes and small scales bordering the eyes predates the evolution of the brille. Another potentially important observation from the integumentary system is the lack of osteoderms in *Retinosaurus*. This is likewise consistent with the allocation of this fossil to the Lacertoidea (all of which lack body osteoderms) or the Xantusiidae (the only scincoidean lineage consistently lacking osteoderms). However, the absence of osteoderms in *Retinosaurus* could also be due to its immaturity, as osteoderms have only rarely been reported in hatchlings and other comparatively small, skeletally immature individuals of some lizard species^60–62^. Additionally, it is unlikely that fusion of the eyelids occurs in posthatching stages, as this event occurs prior to eclosion in other squamates with brilles^63,64^.

There is no unequivocal fossil record of Pan-Xantusiidae outside of North America. Although the Mongolian Late Cretaceous *Eoxanta lacertifrons*^65^ was placed as a sister taxon to Xantusiidae, this attribution has not been supported by subsequent studies (‘Scincomorphaʼ incertae sedis^66^; Globauridae^2^). If correctly placed, *Tepexisaurus* would be the earliest Pan-xantusiid. *Tepexisaurus tepexii* is a well-preserved skeleton from the Lower Cretaceous (Albian, like *Retinosaurus hkamtiensis*) of Mexico^46^ that was originally interpreted as a stem-scincoid. However, Gauthier et al*.*^2^ recovered it as a stem-xantusiid, albeit on the basis of a single character state (282[1]: ectopterygoid hooked posterior process flat and exposed dorsally, ventrally and laterally). The posterior process of the ectopterygoid is well-developed in *R. hkamtiensis*, being exposed in lateral view as well. *R. hkamtiensis* differs from *T. tepexii* in several features (1) interdigitated frontoparietal suture vs. a straight, transversally oriented one; and (2) reduced tooth count (maxillary tooth count 17 vs. 23, premaxillary 9 vs. at least 13 [note, however, that the tooth count in lizards should not be interpreted as absolute due to its variability and this character state is far less informative in regards of differences at generic level. Moreover, tooth number increases through ontogeny]). In any case, many important characters are unknown in *T. tepexii,* e.g., whether the parietal is paired or not (this taxon requires a detailed revision using micro-computed tomography). There are also remnants of soft tissue preserved on some vertebrae and ribs. These appear to be patches of granular or hexagonal integumentary scales (see Reynoso and Callison^46^: fig. 3) resembling the condition in *R. hkamtiensis* in which, small scales ranging from rectangular to hexagonal shape cover the dorsal surface of the body.

Stem-xantusiids are purportedly also represented by contogeniids, the earliest of which is *Utahgenys evansi*^67^ from the Turonian of Utah. Contogeniids reportedly^67,68^ resemble extant xantusiids—in (1) presence of a distinct coronoid articulation facet on the medial surface of the coronoid process of the dentary; (2) dentary posteroventral process distinct and much larger than coronoid process; (3) posteroventral process of dentary reaches further posteriorly than coronoid process; and (4) a distinct, ventrally directed curvature of the supradental shelf of the maxilla behind the tooth row. However, Nydam and Fitzpatrick^67^ placed contogeniids on the stem of Xantusiidae as they retained an open Meckelian canal on the dentary and a separate splenial (in contrast to the spleniodentary of xantusiids). Nonetheless, it is important to bear in mind that none of the contogeniid taxa is represented by any significant skull material, except for the more or less fragmentary jaws, and no postcranial material is available, so their assignment (at both family and higher clade level) remains speculative. Thus, comparison with *Retinosaurus* is restricted to jaws. *Retinosaurus hkamtiensis* shares some character states with contogeniids (1, 2 and 3 above, including an open Meckelian canal on the dentary, which is the plesiomorphic condition present in most lizards), but it differs from contogeneiids in, e.g., tooth morphology: (1) presence of moderately pointed and unicuspid tooth crowns vs. truncated tooth crows (tricuspid in *Utahgenys*); and (2) tooth crowns without anteroposteriorly directed apical groove.

However, although our analyses mostly placed *Retinosaurus hkamtiensis* on the xantusiid stem, this position is not supported by an unequivocal suite of derived characters, nor does it have strong Bremer support (2). Moreover, alternative analyses, like that in which characters were treated as unordered, recovered *Retinosaurus* in a different position, as sister to amphisbaenians. This situation, where the placement of an early Cretaceous lizard is poorly supported and quite labile, is not exceptional. It has been reported for many Jurassic and Cretaceous squamate taxa (e.g., *Scandensia*, *Meyasaurus, Yabeinosaurus,* and *Oculudentavis*; see Vidal^36^; Evans and Barbadillo^51,69^; Dong et al*.*^70^; Bolet et al.^17^), some of which (e.g. *Meyasaurus*, *Yabeinosaurus*) are known from multiple complete specimens.

### Palaeobiogeography

The Burma Terrane has been reconstructed as part of a Trans-Tethyan island arc at the time of amber deposition^26,27^, with the amber biota representing an endemic island fauna, possibly of Gondwanan origin^26^. Although the phylogenetic position of the late Early Cretaceous *Retinosaurus hkamtiensis* gen. et sp. nov. remains uncertain, analyses based on the existing evidence recovered *R. hkamtiensis* as a scincoid lizard (i.e. Scinciformata) and as the sister taxon to the Pan-Xantusiidae clade (sensu Gauthier et al*.*^2^). Xantusiidae is endemic to North and Central America and comprises 34 species in three genera (*Xantusia*, *Lepidophyma*, and *Cricosaura*) that have a fragmented distribution in the Americas^2,71^. Early xantusiid history is potentially documented by *Catactegenys solaster*^54^ (this taxon was also included in our phylogenetic analyses) from the late Campanian Aguja Formation of southern Texas. *Catactegenys solaster* is based only on isolated jaw fragments, but shares features with crown xantusiids, including a closed Meckelian canal and a fused spleniodentary. If the Early Cretaceous Mexican species *Tepexisaurus tepexii* lies on the xantusiid stem, it places the group on the North American continent by the Albian.

If the recovered position of *R. hkamtiensis* is corroborated (e.g. by more complete and mature specimens), it might indicate that Pan-Xantusiidae had a wider distribution in the past. The divergence time of Xantusiidae and Cordyliformes is estimated to have occurred in the Jurassic^7^. During that time, some portions of Myanmar were still connected to the North Coast of East Gondwana—the Burma Block could not have rafted from Gondwana to SE Asia before the Early Cretaceous^26^. The ancestors of *Retinosaurus* might have survived for about 50 million years in these islands, which would explain their presence here, while another radiation moved to North America. Note, however, that other hypotheses about the origin and paleoposition of the Burma Terrane microplate exist (Lich et al*.*^72^). The topic is still rather controversial and leaves room for further interpretations regarding the origin of the animal lineages occurring there during the Cretaceous.

## Conclusions

**Figure 5:**
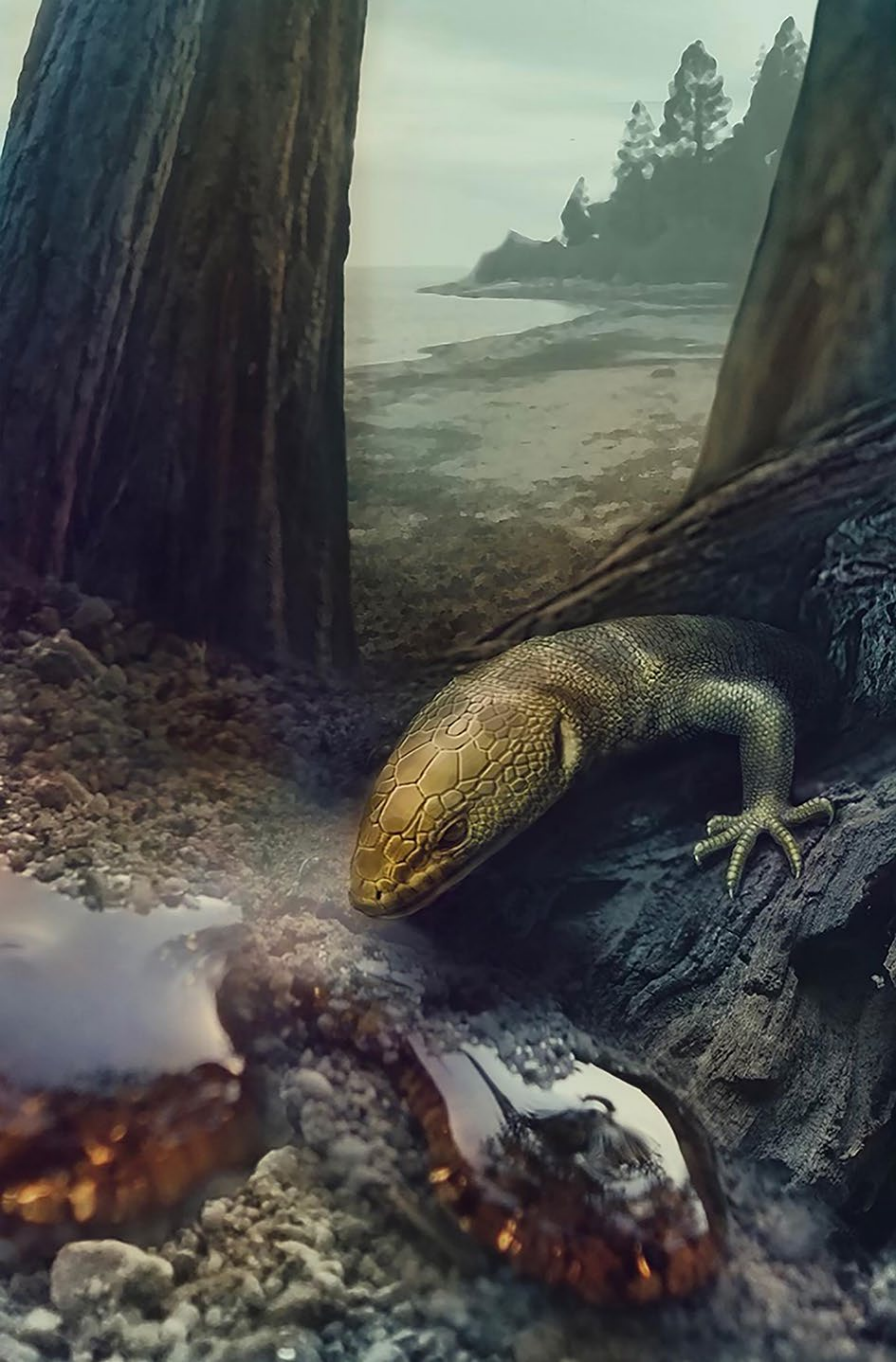
*Retinosaurus hkamtiensis* prior to being trapped in tree resin 110 mya (Scientific illustration by Stephanie Abramowicz).

Among fossils in general, those preserved in amber represent a rare and unique insight on extinct organisms. Amber often contains 3-dimensionally preserved animals (or their parts), frequently including soft tissues^73^. Exclusively based on such preservation, we know that the typical scaling pattern of at least some groups of lizards had already evolved by the Cretaceous, e.g., the granular head scaling and adhesive toe pads of geckos^11,12^. Myanmar amber is also important in yielding unusual taxa from a forest ecosystem rarely represented in the fossil record^12^. Most of the Myanmar vertebrate fossils described to date were recovered from amber sites of Cenomanian age, but *Retinosaurus hkamtiensis* was trapped in an araucarian tree resin^74^ from a site dated to the late Early Cretaceous (Albian, 110 mya, Fig. 5). Although the precise phylogenetic position of *R. hkamtiensis* remains open, this fossil has exceptionally preservation of the integument and provides a rare glimpse of the external appearance of one group of lizards during the Early Cretaceous.

## Methods

### Specimen sourcing

The specimen was acquired ethically from a government licensed gem dealer in 2019, in a Myanmar government approved show, and was subsequently exported legally from Myanmar. In this paper, we follow a very strict protocol as to the origin of the amber piece, its acquisition, and the legalization of its final repository. The material came from a non-conflict zone in the Hkamti area^32^ and, at the time of acquisition, the Sea Sun Star company dominated the mining operations. This company is not listed in the United Nations Human Rights Council report as being involved in the Myanmar conflict^75^. Detailed information on the ethical acquisition of PMF Ref-29689 specimen can be found in the following link: https://bit.ly/2x8gnVj. Paper trail, invoices, and customs forms are also available from the Peretti Museum Foundation website (www.pmf.org).

### Scanning

Microtomographic measurements of the specimen were performed using the Imaging and Medical Beamline (IMBL) at the Australian Nuclear Science and Technology Organisation’s (ANSTO) Australian Synchrotron, Melbourne, Australia. For this investigation, acquisition parameters included a pixel size of 5.8 × 5.8 μm, monochromatic beam energy of 28 keV, a sample-to-detector distance of 100 cm and use of the “Ruby” detector consisting of a PCO.edge sCMOS camera (16-bit, 2560 × 2160 pixels) and a Nikon Makro Planar 100 mm lens coupled with a 20 μm thick Gadox/CsI(Tl)/CdWO4 scintillator screen. As the height of the specimen exceeded the detector field-of-view, the specimen was aligned axially relative to the beam and imaged using three consecutive scans, each consisting of 1800 equally spaced angle shadow-radiographs with an exposure length of 0.50 s, obtained every 0.10° as the sample was continuously rotated 180° about its vertical axis. Vertical translation of the specimen between tomographic scans was 11 mm. 100 dark (closed shutter) and beam profile (open shutter) images were obtained for calibration before and after shadow-radiograph acquisition. Total time for the scan was 52 min. The raw 16-bit radiographic series were normalized relative to the beam calibration files and combined using IMBL Stitch software to yield a 32-bit series with a field-of-view of 14.8 × 29.4 mm. Reconstruction of the 3-D dataset was achieved by the filtered-back projection method and TIE-Hom algorithm phase retrieval^76^ using the CSIRO’s XTRACT^77^. The reconstructed volume data were segmented, rendered and visualized using VGStudio Max 3.5 (Volume Graphics, Heidelberg, Germany).

### Phylogenetic analysis

The new specimen was retrofitted into an expanded version of the morphological character-taxon matrix of Gauthier et al.^2^ (see Supplementary Data 1, 2) with 14 additional terminals, including *Catactegenys*^54^, with rescores of *Eichstaettisaurus*^78^, *Jucaraseps*^44^, and several polyglyphanodontians^79^. A combined evidence analysis was performed, merging the morphological data matrix with a molecular partition that included 140 extant terminals (from Zheng and Wiens^80^). The complete data set includes the molecular partition with 52 genes in a supermatrix and morphology, and all fossil terminals. We were able to score *Retinosaurus* for 384 of 610 characters in the morphological data set of Gauthier et al*.*^2^, plus 65 of 81 characters from the additional characters of Reeder et al*.*^59^, which are mostly external scalation characters, making a total of 449 characters out of 691 morphological characters (65%). While our preferred result is the total evidence matrix, with characters scored as additive following Gauthier et al.^2^ (Fig. 4), the immaturity of the specimen may have led to some characters being mistakenly scored as plesiomorphic. For this reason, we made an exploratory analysis in which we scored as unknown the characters in *Retinosaurus* that could be affected by ontogeny. In this second analysis a total of 429 characters out of 691 (62%) were scored for *Retinosaurus*. The data matrix was analyzed using parsimony as the optimality criterion with the software TNT^81^, using *Gephyrosaurus bridensis* as outgroup, and additive characters were ordered as in Gauthier et al.^2^. To explore the stability of the results, we also ran some alternative analyses for both total evidence matrices (with and without characters affected by ontogeny) with all characters scored as unordered (see Table S1). In all cases, the search was performed until 20 hits of minimum length were found. Each hit was run using 20 random addition sequences, each one subject to sectorial searches, ratchet, and tree drift, and fusing trees every 5 sequences^82,83^. Bremer support values^84^ were calculated using TBR swapping, keeping trees up to 10 steps longer. In the combined evidence analyses, wildcard taxa were identified using the results of an increased tree resolution search and by removing up to three taxa in the strict consensus tree. Subsequently a full consensus tree was calculated without considering the position of wilcard taxa in the optimal trees but indicating the alternative positions for all the wildcard groups.

## Supplementary Information

Supplementary Information 1.

Supplementary Information 2.

Supplementary Information 3.

## Data Availability

Digital surface models of the figured fossil specimen GRS 29689 are available on the sketchfab: https://sketchfab.com/3d-models/head-merged-c23aa8be6aba4ff2a4049e163a21c826.
