# Supplementary material for: A new Early Cretaceous lizard in Myanmar amber with exceptionally preserved integument
Source: Sci Rep. 2022 Jan 31;12:1660. doi: 10.1038/s41598-022-05735-5 (PMC8803969; doi:10.1038/s41598-022-05735-5)
Supplement: Supplementary file 1 — Supplementary Information 1. [file 41598_2022_5735_MOESM1_ESM.docx]

**Supplementary Data 1**

**Contents:**

**1. Detailed description and some virtually isolated elements of *Retinosaurus hkamtiensis***

**2. Coleopterans trapped with the specimen**

**3. List of characters**

**4. Results obtained using different data set and character ordering**

**5. Autapomorphies of *Retinosaurus hkamtiensis***

**6. The locality**

**7. References**

**1. Detailed description and some virtually isolated elements of *Retinosaurus hkamtiensis***

*Note:* Standard anatomical orientation system and nomenclature follows Romer^1^ and Evans^2^. Most of the bones described here are visible on Figs. 2 and 3 except the hyoid. This element is figured on Supplementary Fig 1.

***Skull***

**Premaxilla:** This is a T-shaped bone, apparently unpaired. The nasal process is long and slender. It extends posteriorly, separating the nasals for much of their length, and approaching, but failing to contact, the frontal. The bone bears nine tooth positions (five teeth are still attached). The supradental shelf is well-developed. The maxillary process of the premaxilla is short. It is overlapped slightly by the premaxillary process of the maxilla, possibly forming an oval premaxilla-maxillary fenestra (however, this could be the result of postmortem disarticulation of this region).

**Septomaxilla:** The paired septomaxillae are roughly triangular, plate-like elements that, as preserved, are almost completely separated in the midline, except at their posterior tips. Anterolaterally, each septomaxilla contacts the internal ramus of the premaxillary process of the maxilla.

**Maxilla:** Both maxillae are almost completely preserved. There are about 17 maxillary tooth positions (13 teeth are attached on the left maxilla, 15 on the right). The supradental shelf is dorsally convex. Its posterior portion (at around the level of the 13th tooth position, counted from anterior) is expanded medially, forming the palatine articulation. A groove representing the jugal articulation is visible on the posterodorsal surface of the maxilla, and there is a medial ectopterygoid facet on the posteroventral surface. Anteriorly, the premaxillary process is divided into a robust external ramus and a thinner, more dorsomedially oriented internal ramus. The posterior, orbital, ramus is slightly longer than the premaxillary process, reaching roughly half way below the orbit and tapering to a short post-dental ramus. The maxillary nasal (facial) process is well-developed, with a medial inclination of its dorsal portion. The labial surface is perforated by five to six foramina of varying size. The lingual surface of the facial process bears a large, antero-posteriorly elongate recess that forms part of the nasal cavity. Dorsal to it, there is a large facet for the prefrontal. The lacrimal foramen lies between the prefrontal and the maxilla, and there is a narrow groove (clearest on the right maxilla) that runs anteroventrally from the lacrimal foramen to meet the larger nasal recess.

**Nasals:** Both nasals are preserved, but they have been displaced from their normal anatomical positions. This is especially the case for the right bone which is located above the anterior portion of the orbit. The nasals are small relative to other skull roof elements. Each of them forms a slightly dorsally convex, triangular plate, tapered posteriorly and gradually widening anteriorly, where it divides into two long, pointed processes - the premaxillary and anterolateral processes.

**Prefrontal:** This is a roughly triradiate element, with anterior, ventral and posterodorsal processes. The long narrow posterodorsal process tapers to a point and forms the anterodorsal margin of the orbit. In medial aspect, it bears a facet for the frontal. The ventral process supports the orbitonasal flange in its midsection. This forms the anterior wall of the orbit and separates the orbit from the nasal chamber. The anterior edge of this process, as well as the broad, flat anterior process of the prefrontal, firmly contacts the maxilla, but its narrow ventral tip contacts the palatine and is separated from the maxilla by the lacrimal foramen. The internal surface of the prefrontal is deeply excavated for the nasal capsule. There is no separate lacrimal bone.

**Jugal:** This is a slender, L-shaped bone, comprising two processes that lie at a wide, obtuse angle to one another. The long, thin (rod-like) postorbital process is slightly sigmoid, in that it has a pointed dorsal end that gently curves posteriorly. The suborbital maxillary process is shorter and has a triangular cross-section, due to the presence of a medial ridge (sensu Čerňanský *et al*.^3^). At its posteroventral angle, the external surface of the right jugal is pierced by a large suborbital foramen.

**Frontals:** The frontals are paired and separated by a gap, most likely reflecting postmortem dislocation. The median edges of each bone are well ossified and appear to show an irregular structure of rounded interdigitations, suggesting that there would have been a firm interfrontal suture (rather than fusion) in the adult animal. The resulting frontal plate is elongate and relatively broad, with lateral margins that are concave, but not markedly so (interorbital width similar to anterior frontal width and roughly 67% of posterior frontal width). Posterolaterally, each bone is drawn into a narrow rod-like process that fits into the anterolateral edge of the parietal, as part of the laterally interdigitating fronto-parietal suture. On each side, the prefrontal and postfrontal attach to the lateral margin of the frontal, approaching one another but leaving a small portion of the frontal exposed in the orbital margin. In ventral aspect, the subolfactory processes (cristae cranii frontalis) are well-developed below the anterior one third of the frontal, where they curve ventromedially. In the midsection of the frontal, the subolfactory processes are less developed and in the posterior one-third, they are repesented only by a rounded ridge. Posterolaterally, each frontal bears a ventral facet for a parietal tab and, at least on the left element, shows traces of the fronto-parietal interdigitation.

**Parietals:** The right and left parietals are separated in their anterior two-thirds by a frontoparietal fontanelle and an irregular gap that may represent breakage and/or may have surrounded a parietal foramen but is too large to represent the foramen by itself. Both features indicate incomplete ossification of the parietal plate, a juvenile feature. Further posteriorly, the two bones seem to meet in the midline in a short straight suture, but this part of the bone is still only weakly ossified. Together the parietals form a roughly square parietal table with supratemporal processes that run posteriorly and only slightly laterally. The lateral margins of the parietal table are slightly concave and form the dorsal borders of the narrow, anteroposteriorly elongate supratemporal fenestrae (the fenestra is bordered laterally by the postorbital and squamosal). The anterolateral corners of the parietal table are abruptly stepped due to the presence of the facets for the posterior frontal processes. The suture with the frontal is only ossified in its lateral part, where it is seen to be distinctly interdigitated. The posterior margin of the parietal is an inverted W-shape, with the short (roughly 35% of the median length of the parietal table) supratemporal processes projecting posterolaterally, and the posteromedial part of the parietal table expanding into a somewhat triangular extension. Seen in occipital view, the posterior margin of the parietal is on almost the same level as the anterodorsal surface of the braincase (=supraoccipital), although the two bones apparently do not meet in the midline and there is no ventral fossa on the parietal for the processus ascendens of the supraoccipital. In ventral aspect, a pair of low crests border the lateral margins of the parietal table. The ridges continue onto the supratemporal processes, forming a ventrolateral ridge that runs along the lateral margin of the process. This leaves only a narrow surface for adductor muscle origin, suggesting the bite may not have been strong, although this may reflect the immaturity of the specimen. A ventral (epipterygoid) process appears to be absent, but again, this could be ontogenetic.

**Postfrontal:** The postfrontals brace the frontal–parietal suture on each side. Each bone is slender and anteroposteriorly elongate. Its anterior ramus is directed slightly medially, running parallel to the posterolateral margin of the frontal. The slightly broader posterior ramus is oriented roughly anteroposteriorly, expanding posteromedially to a short contact with the parietal.

**Postorbital:** The postorbitals are triradiate and anteroposteriorly elongate. The anterior part of each postorbital is divided into two short processes, whereas the bone tapers posteriorly into a long, needle-like squamosal process. Thus, the shape of the bone resembles the letter Y on its side. The anterior ventral process probably contacted the jugal in life, but this contact has been lost as both jugals and postorbitals have moved from their normal anatomical positions. The contact for the anterodorsal process is unclear, due to the disarticulation, but it probably met the posterior ramus of the postfrontal.

**Squamosal:** The squamosals have a slender, hockey-stick shape, with an elongate anterior portion that contacts the postorbital, and a ventrally curved posterior half that meets the supratemporal along its medial side and the quadrate at its tip. The squamosal forms the posterolateral margin of the supratemporal fenestra. There is no direct contact between the squamosal and the parietal.

**Supratemporal:** This is a long, narrow, element, wedged between the squamosal laterally, and the supratemporal process of the parietal and opisthotic paroccipital processes medially. It is exposed only as a slender splint in the posterolateral corner of the skull.

**Quadrate**: The quadrate is a dorsoventrally elongate, but anteroposteriorly slender element with an oblique orientation. In lateral view, the dorsal portion of the bone curves posteriorly and forms an irregularly rounded cephalic condyle. The condyle differs in shape on the two sides and there seem to have been different patterns of breakage. The tympanic crest is sharp, but narrow, and forms the rim of a relatively deep concha, running the entire height of the bone. Medially, a strong pillar of bone supports the cephalic condyle dorsally and the narrow, saddle shape, mandibular condyle ventrally. A small foramen perforates the bone slightly above the mandibular condyle, and another pierces the quadrate at approximately the mid-height of the posterior surface. As revealed by micro-CT, the two foramina are connected by a canal and may have carried the chorda tympani nerve.

**Vomer:** The vomers form the anteromedian portion of the palate, anterior to the palatine. They are anteroposteriorly elongate, extending backwards beyond the anteriormost contact of the palatine with the maxilla. The vomers are in median contact almost along their entire length, but the CT scans reveal that they are not fused. The anterior portion of the vomer is flattened, but the rest of the bone is slightly convex in ventral view and bears a deep longitudinal depression on the dorsal surface which floored the nasal capsule. The vomers are widest posteriorly, but immediately anterior to the mid-section, they taper abruptly toward the anterior tip. The posterior region of the vomer is tapered and is wedged into the anterior part of the palatine. Foramina on the palatal surface near the midline (sensu Gauthier *et al*.^4^) are paired. With the maxillae in articulation, the anterolateral corners of the vomers meet, or almost meet, the supra-alveolar shelves of the maxillae, separating each posterior choanal opening from a smaller, slit-like vomeronasal opening. There are no vomerine teeth. The vomeronasal nerve exit appears to be dorsal to the vomer.

**Palatine:** The palatines are trapezoidal bones that bifurcate anteriorly to clasp the vomer. They have a short anterior contact and are then separated in the midline by the interpterygoid vacuity. The medial branch of the palatine vomerine process is longer than the lateral branch and tapers anteriorly. The maxillary process narrows as it extends laterally, before expanding again into a broad and anteroposteriorly elongate contact with the maxilla. The palatine dorsal canal (sensu Gauthier *et al*.^4^) is shallow. A low transverse ridge crosses the anterior part of the palatine dorsally and makes contact with the orbitonasal flange of the prefrontal. The ventral surface is grooved by a short, broad choanal fossa of moderate depth lying between the maxillary and vomerine processes. The fossa gradually decreases in depth toward the posterior end of the element. The pterygoid process extends posteriorly. The palatine, together with the pterygoid and ectopterygoid, border a large, ellipsoid suborbital fenestra. There are no palatine teeth.

**Ectopterygoid:** Only the right ectopterygoid is completely preserved. It is oriented posteromedial to anterolateral and forms a bridge between the pterygoid and the maxilla. The ectopterygoid is an axe-shaped element consisting of lateral and medial parts separated by a short neck. The lateral part is elongated and extends forward to contact the maxillary process of the palatine. Thus, the maxilla is excluded from the suborbital fenestra. The posterolateral tip of the maxillary process is slightly hooked.

**
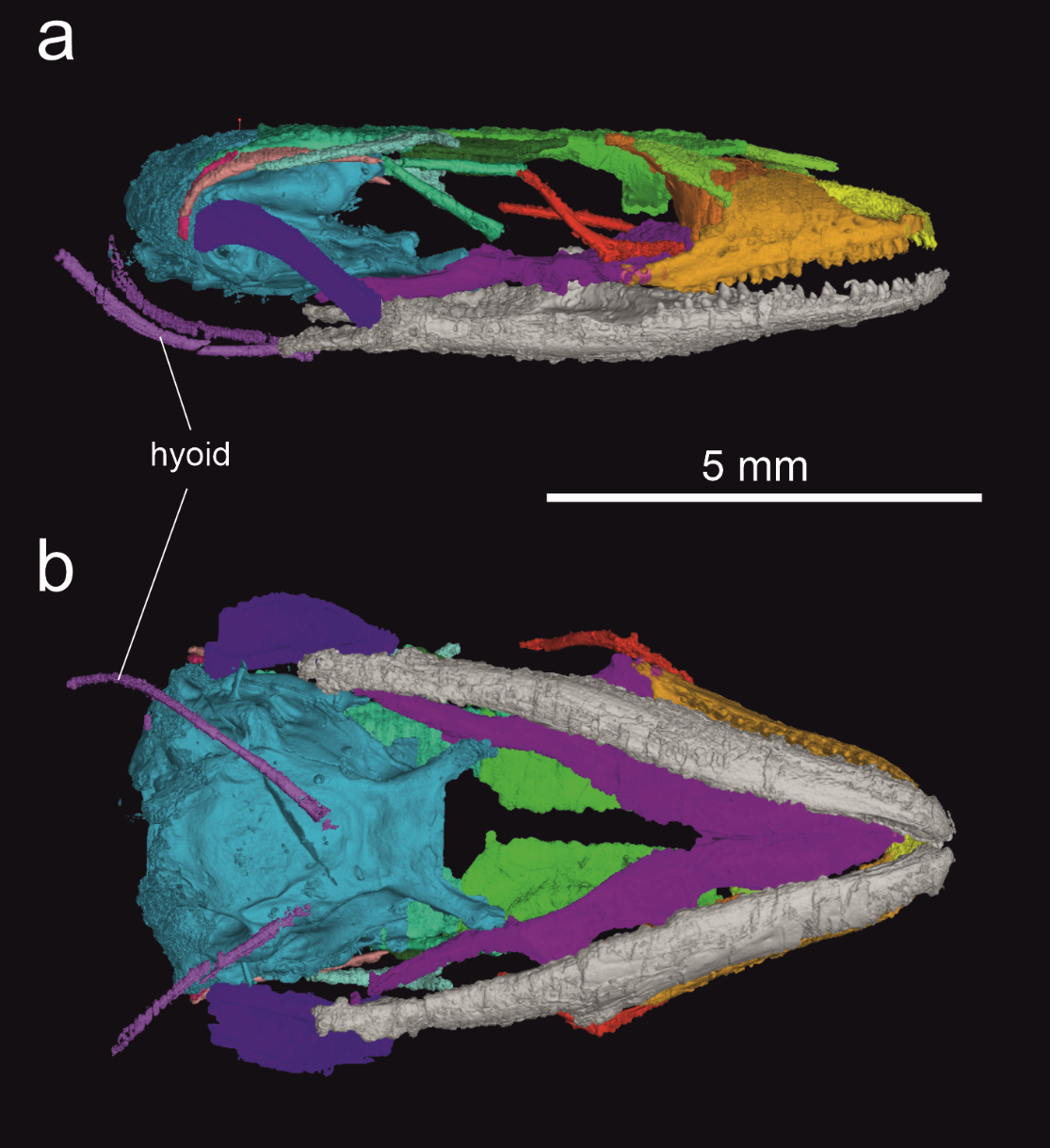
**

**Supplementary Fig. 1.** *Retinosaurus hkamtiensis* gen. et sp. nov. The position of the hyoid in **a** lateral; and **b** ventral views.

**Pterygoid:** The pterygoids are widely separated by the interpterygoid vacuity. They are triradiate, Y-shaped elements that are the longest of the palatal bones. The anterolaterally oriented transverse process is narrow, pointed, and contacts the ectopterygoid posterolaterally in a wedge-like articulation. This transverse process is of similar length to the robust, flattened palatine process, which is oriented anteromedially. The obtuse process (sensu Klembara^5^) is small, and present only as a bulge. The elliptical dorsal epipterygoid fossa (fossa columellae) lies posterior to the obtuse process, at the mid-region of the pterygoid. The quadrate process is long and curves weakly posterolaterally, ending well anterior to the level of the occipital condyle. There are no pterygoid teeth.

**Epipterygoid:** Both epipterygoids are preserved, but they have been displaced from their normal anatomical position, especially on the right side. Given the position of the epipterygoid fossa on the pterygoid, the epipterygoids would have been located entirely anterior to the prootic (Fig. 3). They are elongated, columelliform elements, the bases of which are slightly broader than the dorsal tips.

**Hyoid:** The paired first ceratobranchial elements are preserved, diverging posterolaterally and curving dorsally (Supplementary Fig. 1). They are long, slender rods, in which the curvature is more distinct in the posterior portion.

***Braincase***

**Supraoccipital:** The supraoccipital forms the dorsal portion of the braincase (Supplementary Fig. 2a, c, e, f), capping the otic capsule, and forming the dorsal margin of the foramen magnum. An ossified ascending process (processus ascendens) is absent and the supraoccipital seems to lie at roughly the same level as the posterior margin of the parietal. It is possible that they met, or overlapped, in the adult skull. The central body of the supraoccipital is anteroposteriorly short, but the bone is expanded laterally. In dorsal view, these lateral expansions extend anteriorly into triangular bony processes that contact the prootic. In anterior view, these processes extend ventrally and the endocranial opening of the endolymphatic foramen is visible perforating this inner face of the supraoccipital. The


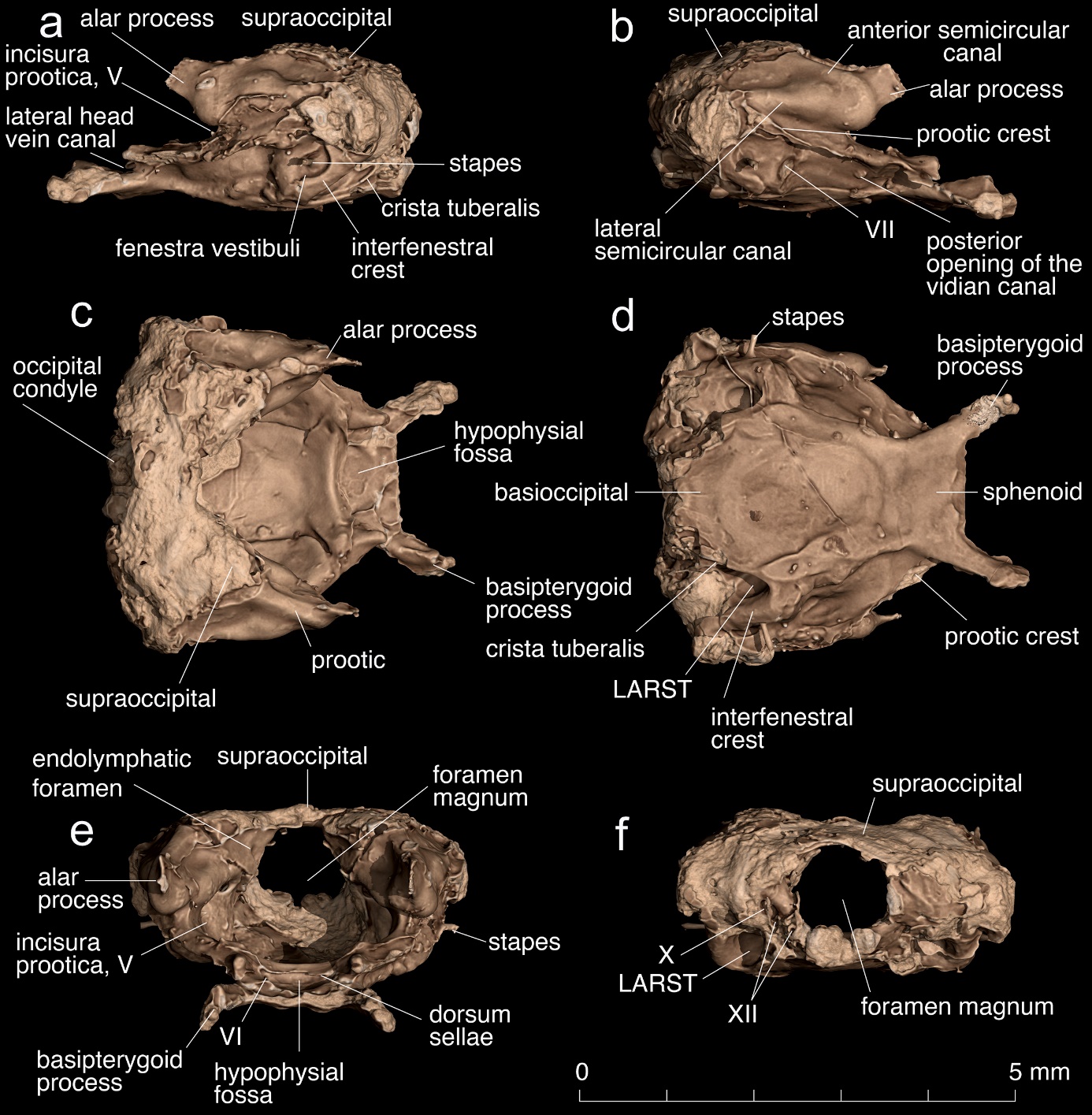


**Supplementary Fig. 2** *Retinosaurus hkamtiensis* gen. et sp. nov. Virtually segmented braincase in **a** left; **b** right; **c** dorsal; **d** ventral; **e** anterior; and **f** posterior views.

posterior border of the supraoccipital is sinuous and forms the anteriorly concave margin of the foramen magnum.

**Prootic.** The prootics form the dorsolateral borders of the braincase (Supplementary Fig. 2b, c). A well-developed anterodorsal alar process is present and forms a blunt ended plate. The dorsal part of the prootic body bulges laterally and, mostly, medially, marking the passage of anterior semicircular canal. Below it, on the lateral surface, is a second rounded ridge marking the passage of the lateral semicircular canal. Ventrolateral to the ridge so formed, the lateral surface is extended into a prootic crest (crista prootica), oriented from posterodorsal to anteroventral. A small foramen for the facial nerve opens ventromedial to the anteriormost part of the prootic crest, and further posteriorly, the prootic forms the anterior margin of the fenestra vestibuli.  Medially, the bone contains a large, concave, acoustic recess from which anterior and posterior branches of the vestibulocochlear nerve enter the inner ear.

**Oto-occipital.** The oto-occipitals are formed by co-ossification of the opisthotics and

exoccipitals (Supplementary Fig. 2a, b, f), the line of demarcation between them being more obvious medially than laterally. The vagus foramen is visible on the boundary between the two, and the exoccipital portion is perforated by at least one small foramen for the hypoglossal nerve. Further anteroventrally, an expanded lateral opening for the recessus scale tympani is framed by crests from the opisthotic (interfenestral crest) and exoccipital (crista tuberalis) dorsally and posteriorly, and by the basioccipital ventrally. Together these form a deep chamber, with a large perilymphatic foramen opening into its roof. The large recess is separated from the more anterior fenestra vestibuli by the thick interfenestral crest. The medial opening of the recessus scalae tympani is much narrower. The exoccipital component forms the lateral borders of the foramen magnum and contributes to the occipital condyle. The paroccipital processes are robust, but very short and not markedly expanded at their tips.

**Basioccipital.** The basioccipital is a broad element, forming the posteroventral

portion of the braincase and the central part of the concave occipital condyle (Supplementary Fig. 2d, f). It contributes to the ventral margin of the compensatory window into which the recessus scalae tympani opens laterally. The bone is roughly trapezoidal in ventral view. The basal tubera are very weakly developed and form little more than a slight ventrolateral ridge, although this may have changed with maturity.

**Sphenoid.** The sphenoid (fused basisphenoid and parasphenoid) lies anterior to the basioccipital (Supplementary Fig. 2d, e). The basipterygoid processes, which articulate with the pterygoids, are large and extend anterolaterally. Their distal ends are slightly thickened, but not expanded. A cultriform process is absent, but whether due to breakage or failure to form is unclear. Laterally, a broad crest-like extension of the sphenoid extends out above the root of the basipterygoid process, creating a partial canal that may be for the lateral head vein. Posterior to this, the bone is pierced by a second foramen that represents the posterior opening of the vidian canal. On the internal side, the alar processes are well-developed, medially connected by a ridge, forming the dorsum sellae. Abducens nerve foramina perforate the lateral margins of the dorsum sella opening into small recesses (for lateral rectus muscle attachment) on either side of the large central hypophyseal fossa. Foramina for the internal carotid arteries open into the posterolateral corners of the hypophysial fossa (clearest on the right). The vidian canals open anteriorly just medial to the base of each basipterygoid process.

**Stapes:** The stapes is preserved on both sides (Supplementary Fig. 2d). It is a small, straight bone with a relatively short, unperforated cylindrical shaft. The footplate is rounded, and fits within the fenestra vestibuli of the braincase, between the prootic and otooccipital.

**The inner ear:** A digital endocast was made of the inner ear region, based on the CT scan data (Supplementary Fig. 3). As reconstructed, the inner ear has a compact appearance. The vestibule, which houses the statolithic mass, is separated from the cochlear recess by a constriction. The anterior ampullary recess is connected to the vestibule. The anterior semicircular canal leaves the anterior ampullary recess in the prootic and turns posterodorsally to meet the crus commune in the supraoccipital. The lateral semicircular canal runs posteriorly from the lateral ampullary recess, to reach the level of the posterior ampullary

**Supplementary Fig. 3** *Retinosaurus hkamtiensis* gen. et sp. nov. Virtually segmented endocast e of the inner ear region in **a** lateral; **b** dorsal views.

recess in the oto-occipital, crossing the suture between otoccipital and prootic. The posterior semicircular canal extends posterolaterally from the posterior ampullary recess, then turns dorsally to meet the crus commune. The ventral cochlear recess resembles a triangular pendant. It contained the auditory organ or basilar papilla. Anteromedially, there is a small swelling – reflecting the cavity of the endolymphatic duct recess. Unlike the inner ear of arboreal lizards^6,7^, the digital endocast reveals that the semicircular canals are situated relatively close to the vestibule, but not as close as in head-first burrowers^8^.

***Mandible***

**Dentary:** The dentary is a slender, elongate element (Fig. 3 e, f), with a slight medial curvature at its symphysial end where the two dentaries meet at a short contact. It is longer than the post-dentary part of the mandible. The dentary bears approximately 20-22 tooth loci, with many teeth showing active lingual replacement. The Meckelian canal is narrow, but fully open. It is roofed by the subdental shelf and narrows at the level of the 14th tooth position due to presence of the splenial facet on its ventromedial margin. The internal alveolar foramen is located at the level of the 18th tooth position. The labial surface of the dentary is pierced by a row of six neurovascular foramina. The dentary bears a strong coronoid process, and a longer ventral angular process that extends posterior to the dorsal process of the coronoid. The process fits into an anterior notch in the angular.

**Splenial:** The splenial is best preserved in the left mandible (Fig. 3f). It is an independent, mediolaterally flat element. The bone is short, reaching only the level of the 14th tooth position anteriorly. An anterior inferior alveolar foramen perforates the anterior section, at the level of the 17th tooth position. An anterior mylohyoid foramen is located posteroventral to it, at the level of approximately the 18th tooth position.

**Coronoid:** Both coronoids are preserved (Fig. 3e, f). The coronoid process is large, triangular and dorsally extended, so that it is exposed in lateral aspect. The anterolateral margin bears a ridge for muscle attachment. The bone also has a short anteromedial process that does not extend forward beyond the posterior end of the tooth row and overlaps the external surface of the mandible to a small extent. Ventrally, the anteromedial process of the coronoid abuts the splenial. The posteromedial process is long and ventrally extended. It has a twisted appearance and, on its medial side, a strong muscular crest forms the anterior border of the adductor (mandibular) fossa.

**The posterior mandible region:** The posterior parts of the lower jaws are embedded in soft tissue matrix of a similar density to bone. Therefore, it is difficult to determine the separation vs. fusion of the bones located here. The surangular is an elongated element, with a roughly horizontal dorsal margin that rises slightly posterodorsal to the coronoid. Here, at the level of the vertical posterior margin of the coronoid, the surangular is pierced by the anterior surangular foramen. At its posterior end, the bone expands slightly and forms the anterior border of the glenoid fossa. A posterior surangular foramen perforates the bone slightly in front of the fossa. In medial aspect, the bone participates in a long, but shallow adductor fossa.

The angular is a long, slender bone that is exposed on the ventrolateral side of the mandible. The posterior mylohyoid foramen is visible in medial view, located at the level of the coronoid process.

As usual in squamates, prearticular and articular appear to be fused. The prearticular extends anteriorly to the level of the coronoid. The articular is short and robust, forming most of the dorsal glenoid fossa for the quadrate. The oblique orientation of the quadrates is reflected by the steep, almost vertical joint surface of the articular. The bone bears a long, straight, posteriorly directed retroarticular process, that gradually narrows toward the tip.

**Dentition:** The tooth implantation is pleurodont with the tooth bases ankylosed to their sockets. The dentition is homodont, with the teeth appearing to be of constant height along the tooth row. Along the row, the teeth are separated by an interdental gap roughly equal to 0.5 of the mesiodistal width of the tooth neck, with the gap increasing in the posterior part of the tooth row. The teeth are generally slender, although they increase slightly in robustness posteriorly. Each tooth is conical, with a crown that is slightly flattened lingually, giving them a concave appearance in lingual aspect. In the anterior part of the tooth row, the tooth apices are moderately pointed and unicuspid, with a slight posteromedial curvature. Some of the tooth bases are eroded by lingual resorption pits, and some of the empty tooth positions contain the crowns of replacement teeth.

***Postcranial skeleton***

The specimen preserves parts of the anterior vertebral column, pectoral girdle, and forelimb.

A total of 23 presacral vertebrae are preserved, with ten anterior vertebrae clearly visible, and 13 further vertebrae and their ribs enclosed in a calcite sheath that partially obscures their structure. Without the sacrum, it is not possible to estimate the total number of dorsal vertebrae. However, based on the fully developed forelimbs, the presacral vertebral number is likely to have been within the range of normal limbed squamates, such as gekkotans (23–30) and xantusiids (26–30), but far from presacral counts of 40 where limbs become strongly reduced in size, or 50 where limb loss is evident^9^. The sternal plate is not recoverable, but there were an estimated seven or eight cervical vertebrae (atlas, axis, one vertebra with no cervical ribs, three vertebrae with short cervical ribs, and one vertebra with a long rib that does not seem to be connected to the sternum. The next posterior vertebra has a long rib that may or may not connect to the missing sternum – although the overall preservation is rather poor, the condition in *Retinosaurus* resembles that present in many extant lizards, in which the first two long ribs do not attach to the sternum. The pedicles of the atlantal neural arch are slender, whereas the arrow-shaped laminae are broad. There is a well-developed zygapophyseal articulation between the atlas and axis. The 1st and 2nd intercentra are small, and both are keeled, as are also the third to fifth intercentra. The more posterior vertebrae are broader in dorsal view, with only a small interzygapophyseal constriction. Posterior cervicals and dorsals do not appear to bear intercentra. The neural canal is large, and the neural arch pedicel is slender in lateral view (these are juvenile features). The centra are incompletely developed but they do appear to bear small posterior condyles, indicating procoely (the procoelous type of the centra can be recognized especially when CT horizontal slices are observed). There is no visible zygantrum-zygosphene articulation, although this may reflect immaturity. As mentioned above, the first rib is associated with the 4th cervical vertebra. Vertebra 21 bears a shortened rib, and the two damaged vertebrae following it also seem to bear short ribs. This suggests they are posterior dorsals, a few vertebrae anterior to the sacral region.

The pectoral girdle comprises the unfused scapula and coracoid, the interclavicle, and the clavicles. The scapula is slender with a curved anterior margin but no distinct emargination. It is separated from the coracoid and the latter bears a deep primary emargination as well as a large coracoid foramen. The interclavicle is cruciform, with a relatively long anterior process (~14% of the length of the posterior stem), and the slender clavicles meet at its tip, leaving a large gap between themselves and the lateral processes of the interclavicle. The left clavicle is the best preserved and it is distinctly sigmoid, with a posterior curvature of its medial tip, where it meets the interclavicle, and a pronounced angulation in its midsection so that the lateral one third of the bone ascends sharply upwards to meet the anterodorsal corner of the scapula. Both forelimbs are preserved. The humerus is a robust bone with expanded proximal and distal ends oriented in different planes, but no ossified epiphyses. There is a short proximal deltopectoral crest, and distally a small ectepicondylar foramen. The radius and ulna are shorter (~64% of humeral length) and have only a slight proximal and distal expansion. The ulna lacks an olecranon, but this is probably ontogenetic. The carpus is, at most, weakly ossified. The metacarpals are relatively short, especially that of the fifth digit, but the penultimate phalanges are relatively long, supporting ungual phalanges that are short, broad, and tapering at their tips. The phalangeal formula is 2:3:3:4:3, with the two intermediate phalanges on digit 4 being unusually short.

**Orbital region**

The left side of the skull preserves an intact circumorbital series, within which rectangular scleral ossicles are preserved, seven of which are visible in dorsal view (inset, Fig. 4). If the ventral ossicles are similar in size and spacing to the dorsal ones, the eye would have at least 14 scleral ossicles in total. The left eye also has preserved the eyelids, defining a horizontal palpebral fissure, and was clearly not an eye covered by a brille or spectacle. At least four large scales and several tiny scales cover the eyelid.

***Lepidosis***

Several characters preserved in the *R. hkamtiensis* specimen are shared with xantusiids (e.g. rostral scale expanded dorsally, and a single row of enlarged supraoculars between superciliary and frontal), but these characters are not exclusive to xantusiids. In fact, there are a few scale characters that are present in *R. hkamtiensis* but not in extant xantusiids (e.g., a single unpaired postmental scale).

The cephalic scales are enlarged and well preserved (Fig. 1) and follow a regular dorsal scutellation pattern of alternating single and paired scales from front to back of the skull roof. There is a single enlarged rostral, paired supranasals, unpaired nasal and frontal scales, and paired parietals. The narial opening is surrounded by the rostral, supranasal­­­­, postnasal, and the first supralabial. Behind the postnasal, there are two square loreals. There is an unpaired hexagonal nasal following the prenasal, and large and shield-like frontal (slightly larger than the two frontoparietals combined). The paired frontoparietals are pentagonal. There are four supraoculars, and the eyelids are bounded dorsally by a row of supracilliaries. The parietals are separated by the interparietal­. The left parietal scale is not well defined, but the right one shows a well-defined oblique contact with the frontoparietal. The interparietal seems to be heptagonal in shape. In the center of the interparietal, there is a raised and thickened circular scale, which could be the position of the parietal eye. This scale projects ventrally into the interparietal suture, although it is possible that this could represent a scale shifted from another part of the head. There are eight rectangular supralabials and infralabials. The supralabials reach the level of the orbit but are separated from the eyelids by at least three rows of small scales. The occipital scales and the scales in the temporal region have a rhomboid shape. Smaller scales ranging from rectangular to hexagonal cover the dorsal surface of the body, but those on the ventral side are larger and rectangular. On the ventral side of the head, there is a large and triangular mental scale, followed by two pentagonal postmentals (the first pair of chin shields). Following the mental scales, there are four large chin shields, which gradually increase in size antero-posteriorly. In the midline, and bounded by the chin shields, there is a series of smaller, rectangular scales. The gular scales are hexagonal, rectangular, and rounded; these scales are smaller than the chin shields and transition quickly to rounded scales on the ventral side of the neck, where the scales become organized into regular transverse rows. The scales on the ventral part of the neck are different to the ones on the dorsum, which are also organized in transverse rows, but they are squarer, and continue in the same pattern along the preserved part of the rostrum. The palmar surface of the digits bears rectangular subdigital lamellae, proximodistally short and preaxially-postaxially expanded. The claw sheaths are distinctly hooked at their tips. There are no osteoderms preserved, but this character should be treated with caution as, in most modern lizards, osteoderms form post-hatching.

**The trachea and bronchi**

The trachea and bronchi are surprisingly preserved, as revealed by CT, and these elements were possible to segment (Supplementary Fig. 4). The trachea marks the beginning of the tracheobronchial tree. It forms a long tube, which starts anteriorly in the region between pterygoids. The cartilage rings are not visible. The trachea continues posteriorly into the thorax, extending beyond the level of the interclavicle. At this level, it bifurcates into the right and left main bronchi and these, together with the trachea, form a Y-shaped structure. The bronchi are, however, only partly preserved.


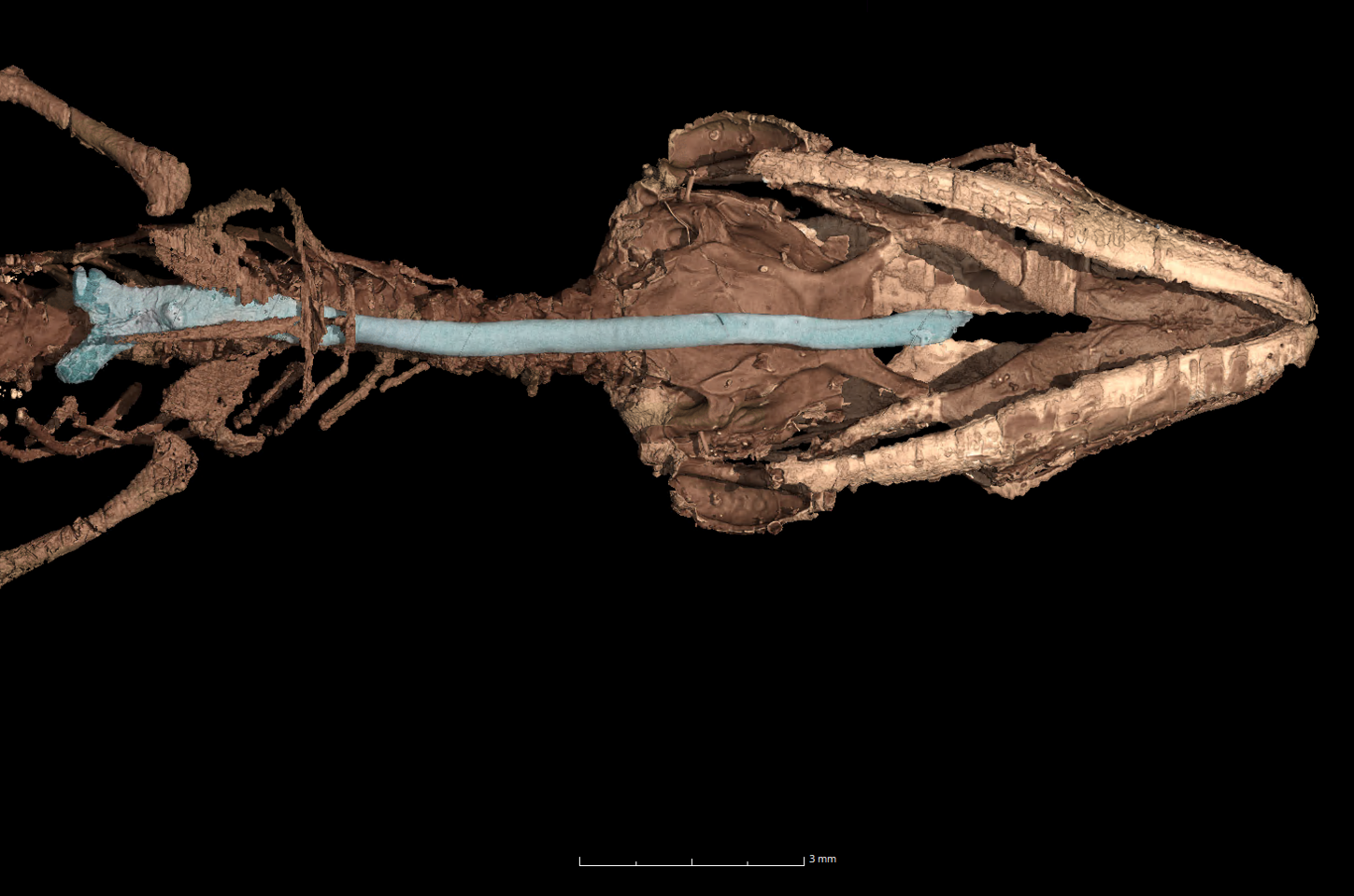


**Supplementary Fig 4.** *Retinosaurus hkamtiensis* gen. et sp. nov. The trachea and bronchi in ventral view, indicated in blue.

**2. Coleopterans trapped with the specimen**

Several coleopterans were trapped with the specimen (see Supplementary Fig. 5). Coleoptera from the Cretaceous are difficult to identify, but at least four different kinds can be recognized and it is very likely that these are necrophagous (Enrique Penalver, pers. comm.). One (Supplementary Fig.5d) with a large antennal club could belong to the Dermestidae, Sphindidae, Erotylidae, Silphidae, or Scarabaeoidea. A second one (Supplementary Fig.5b) has tentatively been identified as belonging to the family Cleridae based on the shape of the antennae, declined head, divided tarsi in the mesotarsi and a fifth being lobed, projected procoxae and large mesocoxae (these features could also apply to the Anthicidae, Mycteridae and Ptinidae). A third kind of coleopteran (Supplementary Fig.5a) was more difficult to identify, but may be be assignable to the Tenebrionoidea based on the possession of antennae without a club, and 4 tarsomeres. Another has long antennae (tentatively classified as Cerambicidae), and there is one beetle that could be attributed to Leiodidae.


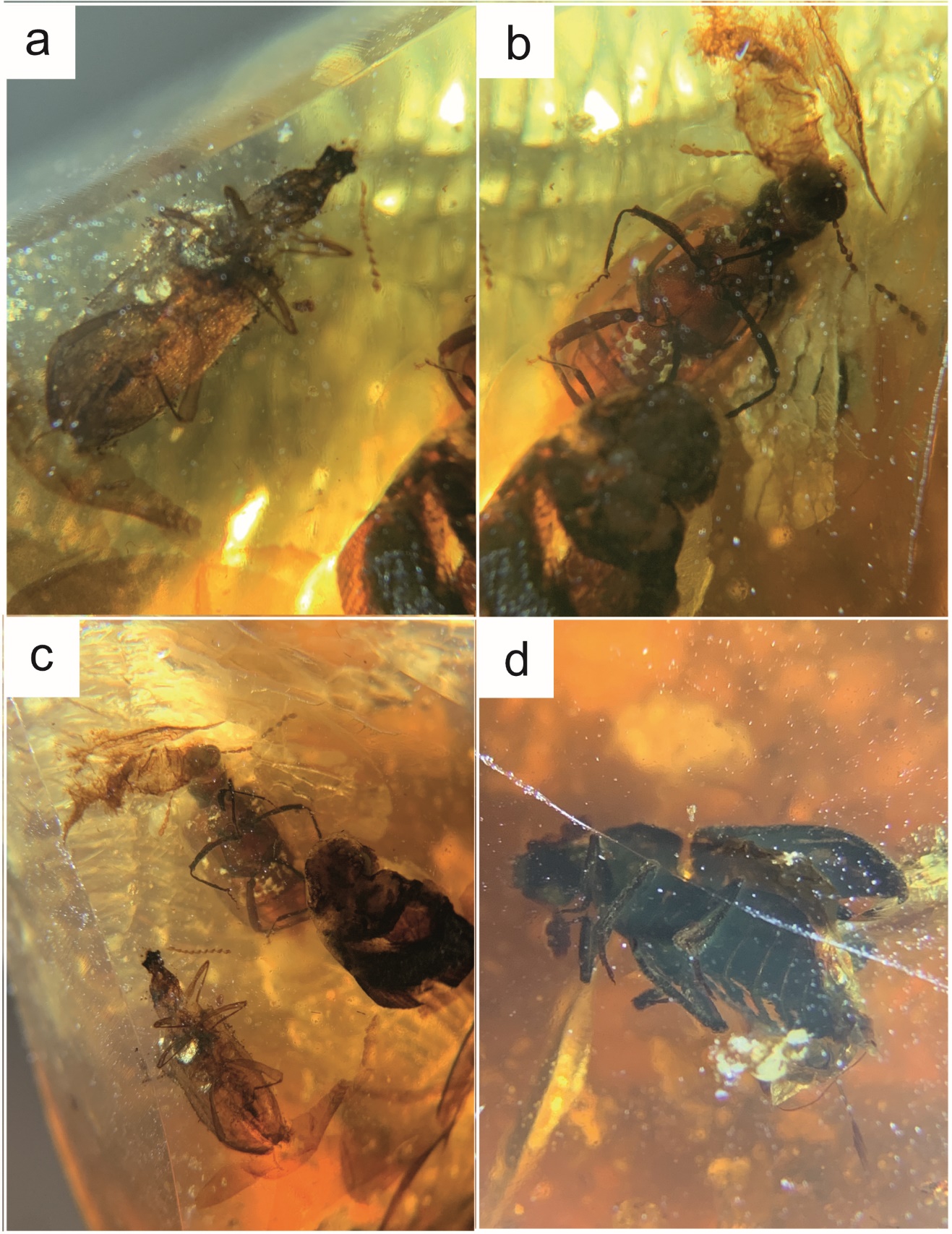


**Supplementary Fig. 5.** Details of some of the beetles preserved with *Retinosaurus* *hkamtiensis*. **a**, Undetermined beetle with 4 tarsomeres on the hind legs, antennae without club, potential Tenebrionoidea. **b**, Beetle potentially of the family Cleridae (Cleroidea) based on the shape of the antennae, head declined, mesotarsi with 5 tarsi being the penultimate lobed, procoxas projected and large mesocoxas, laterally closed, slightly separated. Some of these characters are also present in Anthicidae, Mycteridae, and Ptinidae. **c**, size comparison of **a** and **b**. **d**, This beetle with large clubbed antennae could be a member of Dermestidae, Sphindidae, Erotylidae, Silphidae, or possibly Scarabaeoidea.

**3. Data matrix (Gauthier *et al*.^4^).**

1. Premaxilla: (0) paired; (1) fused. Gauthier, Estes and de Queiroz^10^.

2. Premaxilla palatal shelf: (0) not bifid posteriorly; (1) bifid posteriorly. Rieppel^11^.

3. Premaxilla maxillary process development: (0) normal size; (1) reduced; (2) absent. Moody^12^ (ordered).

4. Premaxilla maxillary process length relative to level of palatine-maxilla suture: (0) premaxilla medial to level of palatine-maxilla suture; (1) premaxilla extends lateral to level ofpalatine-maxilla suture. Gauthier *et al*.^4^.

5. Premaxillary-maxillary fenestra: (0) absent; (1) present. Gauthier^13^.

6. Premaxilla body anterior ethmoidal foramina number: (0) two; (1) four or more. Lee^14^.

7. Premaxilla body anterior ethmoidal foramina exit via: (0) external naris, (1) premaxilla notch, Lee (2000); (2) premaxilla body; (3) between premaxilla and maxilla; (4) in maxilla, Gauthier *et al*.^4^. (ordered).

8. Premaxilla body ventral ethmoidal foramen: (0) small; (1) large; (2) absent. Gauthier *et al*.^4^.

9. Premaxilla-maxilla suture: (0) firm; (1) loose. Cundall^15^ (1995).

10. Premaxilla internasal process length: (0) less than half nasal length; (1) more than half way to frontal between nasals; (2) nearly to, or articulates with, frontal. Kearney^16^ (ordered).

11. Premaxilla internasal process shape in cross-section: (0) subtriangular; (1) compressed; (2) depressed. Gauthier *et al*.^4^.

12. Premaxilla internasal process position relative to nasal descending lamina: (0) premaxilla internasal process lies at level of nasals on skull roof; (1) long internasal process

clasped between descending nasal laminae; (2) short overlap between premaxilla and nasal lamina; (3) lamina abuts posteroventral base of premaxilla; (4) loss of nasal

descending lamina contact with premaxilla. Cundall *et al.*^17^ (ordered).

13. Premaxilla internasal process shape in anterior view: (0) tapers apically or parallel-sided across nares; (1) widens across nares. Gauthier *et al*.^4^.

14. Premaxilla internasal process size: (0) well developed; (1) very reduced/absent. Gauthier *et al.*4.

15. Premaxilla internasal process bifid in lateral view, with ancestral dorsal ramus joined by a deeper ventral ramus (premaxillary keel of Lee and Scanlon 2002) extending posteriorly

off base of internasal process: (0) absent; (1) present. Cundall *et al*.^17^.

16. Premaxilla: (0) without conspicuous vertical margin on maxillary process; (1) with conspicuous vertical margin on maxillary process. Kluge^18^.

17. Nasals: (0) paired; (1) fused. Pregill *et al*.^19^.

18. Nasals anterior width: (0) exceeds nasofrontal joint width; (1) is subequal to nasofrontal joint width. Gauthier^13^; (2) less than anterior frontal width (ordered). Gauthier *et al*.^4^.

19. Nasal-prefrontal suture: (0) present; (1) absent. Estes *et al*.^20^.

20. Nasal-maxilla suture: (0) present; (1) absent. Estes *et al*.^20^.

21. Nasal descending lamina: (0) absent; (1) present, with descending lamina extending below level of nasal-maxilla suture. Gauthier *et al*.^4^.

22. Nasal supranarial process in dorsal view: (0) well-developed; (1) reduced/absent. Gauthier *et al*.^4^.

23. Nasal-maxilla suture in cross section anteriorly: (0) maxilla overlaps nasal at roof of nasal chamber; (1) nasal partly overlaps maxilla dorsally; (2) nasal abuts maxilla; (3) nasal underlaps maxilla to floor of narial chamber. Gauthier *et al*.^4^.

24. Nasals ventral contact beneath premaxillary internasal process: (0) broad contact below; (1) or not in contact except near apex. Gauthier *et al*.^4^.

25. Nasals dorsal contact over premaxilla internasal process: (0) no contact; (1) in contact over apex; (2) broadly in contact (ordered). Gauthier *et al*.^4^.

26. Nasals reduced to narrowly elliptic elements attached to either side of premaxilla internasal process: (0) absent; (1) present. Bell^21^.

27. Nasal shape: (0) not small and cruciform; (1) small and cruciform. Gauthier *et al*.^4^.

28. Nasal length relative to frontal length: (0) nasals shorter than frontals; (1) nasals longer than frontals. Gauthier *et al*.^4^.

29. Nasal anterior extent toward premaxilla: (0) nasal extends anterior to maxillary tooth row or vomer; (1) nasal terminates posterior to end of maxillary tooth row or vomer tip. Gauthier *et al*.^4^.

30. Nasofrontal fontanelle: (0) absent, frontal and nasal firmly sutured; (1) present, poorly ossified suture between nasal and frontal on midline. Moody^12^.

31. Nasofrontal suture shape: (0) without V-shaped nasal process into frontal midline; (1) with prominent V-shaped nasal process into frontal midline. Gauthier *et al*.^4^.

32. Nasal-frontal articulation dorsally: (0) nasals suture in two V-shaped recesses of anterodorsal end of frontal; (1) nasals overlap only onto narrow horizontal shelf dorsally on

frontals. Gauthier *et al*.^4^.

33. Nasal dorsal lamina: (0) in broad contact with dorsal frontal lamina; (1) in narrow (medial, point-) contact with frontal; (2) not in contact with frontal. Rieppel (2007) (ordered).

34. Nasal medial (vertical) flange: (0) in extensive dorsoventral contact with medial frontal flange; (1) in dorsal contact only; (2) in ventral contact only; (3) not in contact with frontal. Rieppel (2007).

35. Nasal medial (vertical) flange, ventral contact with frontal: (0) abutting; (1) reduced to point contact. Rieppel^22^.

36. Frontals: (0) paired; (1) fused. Estes *et al.*^20^.

37. Frontal-maxilla suture: (0) frontal separated from maxilla by nasal-prefrontal contact; (1) frontal contacts maxilla, separating nasal from prefrontal. Gauthier^13^.

38. Frontal subolfactory processes: (0) absent; (1) arch beneath brain but do not contact; (2) arch beneath brain to articulate on ventral midline; (3) arch beneath brain

and fuse on ventral midline. Pregill *et al.*^19^ (ordered).

39. Frontal subolfactory process depth: (0) 25–35%; (1) 42–53%; (2) 58–68%; (3) 75–85%; (4) more than 89%. Gauthier^13^ (ordered).

40. Frontal subolfactory process-parasphenoid suture: (0) absent; (1) present. Lee^14^.

41. Frontal subolfactory process descending lamina-parasphenoid rostrum relationship: (0) absent; (1) descending lamina off frontal subolfactory process (continuation of frontal enclosure of optic nerve) lies dorsolateral to parasphenoid; (2) descending lamina off frontal subolfactory process tightly clasps parasphenoid dorsolaterally. Gauthier *et al*.^4^. (ordered).

42. Frontal subolfactory processes delimit deep narrow canal across most of orbit: (0) absent; (1) present. Gauthier *et al*.^4^.

43. Frontal subolfactory process prefrontal lamina: (0) absent; (1) knob-like process at anteromedial rim of subolfactory process with prefrontal facet; (2) conspicuous

descending lamina off subolfactory process articulating just behind prefrontal; (3) produced into shelf supporting prefrontal ventrally. Gauthier *et al*.^4^. (ordered).

44. Frontal subolfactory process: (0) straight; (1) forms thickened anterolaterally projecting flange. Rieppel^22^.

45. Frontal medial pillar: (0) absent; (1) separated anteriorly from subolfactory process by gap; (2) sutured to subolfactory process; (3) fused with subolfactory process. Rieppel^22^ (ordered).

46. Frontal medial flange separating olfactory tracts: (0) vertically positioned; (1) slants forward (anterior margin of subolfactory process in front of anterior margin of frontal

dorsal lamina); (2) slants backwards (anterior margin of subolfactory process behind anterior margin of frontal dorsal lamina). Rieppel^22^.

47. Frontal descending process-parietal contact, in horizontal section: (0) no contact; (1) parietal overlaps frontal laterally; (2) frontal descending process abuts parietal; (3) frontal descending process overlaps parietal laterally, at least in part. Gauthier *et al*.^4^.

48. Frontal interorbital width/frontoparietal suture width I: (0) 14-19%; (1) 20-22%; (2) 24-26%; (3) 28-34%; (4) 36-40%. Estes *et al.*^20^. (ordered).

49. Frontal interorbital width/frontoparietal suture width II: (0) less than 44%; (1) 44-47%; (2) 50-53%; (3) 55-58%; (4) 60-63%. Estes *et al.*^20^. (ordered).

50. Frontal broadly overlaps prefrontal dosally: (0) absent; (1) present. Crother *et al*.^23^.

51. Frontal supraorbital shelf: (0) absent; (1) present; (2) present and demarcated medially by narrow shallow longitudinal furrow often bearing line of foramina on the dorsal surface of the frontal. Smith^24^ (ordered).

52. Frontal anterior margin shape: (0) mainly trends anteromedially; (1) broadly transverse. Gauthier *et al*.^4^.

53. Frontal anteroposteriorly narrow, blunt prefrontal process off lateral base of subolfactory process extends into prefrontal socket: (0) absent; (1) present. Gauthier *et al*.^4^.

54. Frontal posterior margin convex and parietal anterior margin concave, in mid-sagittal section: (0) absent; (1) present. Gauthier *et al*.^4^.

55. Frontoparietal suture: (0) separate; (1) fused. Kearney^16^.

56. Frontoparietal suture interdigitation: (0) frontal overlaps parietal dorsally; (1) lightly interdigitate or simple abutment; (2) moderate interdigitation; (3) strong interdigitation; (4) deeply interdigitate [Kearney 2003b]). Estes *et al.*^20^.

57. Frontoparietal suture dorsal outline: (0) bowed anteriorly/inverted U; (1) roughly transverse; (2) shallow U or W bowed posteriorly; (3) deeply bowed posteriorly U

or W; (4) frontal postero-dorsolateral corner protrudes posterolaterally. Gauthier, Estes and de Queiroz^10^.

58. Frontal parietal lateral overlap: (0) frontal deeply overlaps parietal; (1) frontal barely overlaps parietal laterally; (2) frontal underlaps parietal laterally. Gauthier *et al*.^4^.

59. Frontoparietal fontanelle in adult: (0) absent; (1) present. Gauthier *et al*.^4^.

60. Frontoparietal suture expression in medial wall of orbit: (0) strongly inclined anteriorly; (1) vertical or slightly inclined anteriorly. Kluge^25^.

61. Frontal suboptic shelves-parietal contact: (0) parasagittal shelves (suboptic shelves) pass from posterior base of frontal subolfactory processes on either side of the dorsal

edge of the parasphenoid to near contact, or overlap, parietal medially below optic foramen; (1) frontal suboptic processes widely separated from parietal on either side of the midline at the ventral junction of frontal, parietal and parasphenoid; (2) suboptic shelves absent. Gauthier *et al*.^4^.

62. Postfrontal: (0) present; (1) absent; (2) fused to postorbital; (3) fused to frontal. Estes *et al.*^20^.

63. Postfrontal shape: (0) triradiate (notched distally or not), with subequal frontal and parietal processes wrapping around frontoparietal suture; (1) parietal process much shorter than frontal process; (2) parietal process absent, postfrontal subtriangular. Estes *et al.*^20^. (ordered).

64. Postfrontal distal shape: (0) tapering to point (passing anterior to postorbital); (1) bifid (clasps postorbital). Gauthier *et al*.^4^.

65. Postfrontal relative to parietal table: (0) ventrolateral; (1) dorsal overlap present; (2) dorsal overlap extensive. Gauthier *et al*.^4^. (ordered).

66. Postfrontal-jugal articulation: (0) widely separated; (1) nearly in contact, but still separated; (2) in contact. Gauthier *et al*.^4^. (ordered).

67. Postfrontal supratemporal shelf: (0) absent; (1) present as thin shelf extending over anterodorsal corner of supratemporal fenestra; (2) extending posteriorly further than laterally

across upper temporal fenestra; (3) to (nearly) occlude upper temporal fenestra. Estes *et al.*^20^. (ordered).

68. Postorbital: (0) present; (1) lost. Estes *et al.*^20^.

69. Postorbital shape: (0) widens anteriorly; (1) narrows anteriorly. Gauthier *et al*.^4^.

70. Postfrontal broad and flat: (0) not; (1) often very broad, always anteroposteriorly

extensive and flat, with postorbital process reduced to nub; (2) with a shaft that is club-shaped distally. Gauthier *et al*.^4^. (ordered).

71. Postorbital-parietal contact: (0) postorbital entirely distal, separated by postfrontal from parietal; (1) postorbital with discrete process extending toward parietal behind postfrontal; (2) postorbital contacts parietal ventrolaterally at frontoparietal suture; (3) or postorbital

dorsolaterally behind frontoparietal suture. Smith^24,26^.

72. Postorbital shape at skull roof contact: (0) postorbital abuts parietal dorsolaterally at narrow contact; (1) with a long anterodorsally curving head (that often extends past level of frontoparietal suture midline). Gauthier *et al*.^4^.

73. Postorbital with small compressed tab at apex passing across frontoparietal suture: (0) absent; (1) present. Gauthier *et al*.^4^.

74. Postorbital, dorsomedial head: (0) undivided; (1) divided into two heads. Kluge^25^.

75. Postorbital squamosal process: (0) present; (1) absent. Estes *et al.*^20^.

76. Postorbital restricts upper temporal fenestra (UTF): (0) absent, postorbital tapers to tip; (1) partly occludes UTF, as postorbital expands medially posteriorly; (2) enlarged postorbital completely occludes UTF. Estes *et al.*^20^.

77. Postorbital (nearly) excludes squamosal from upper temporal fenestra: (0) absent; (1) present. Wu *et al.*^27^.

78. Postorbital overlaps squamosal: (0) laterally into V-shaped recess in squamosal; (1) dorsomedially as slender tapering rod attached superficially; (2) dorsally; (3) postorbital in long V-shaped trough dorsally and then rotating dorsolaterally posteriorly; (4) squamosal lies in trough beneath postorbital. Arnold^28^.

79. Postorbital-squamosal suture: (0) firm, suture no wider than those among surrounding elements; (1) loose, sutural gap wider than that between postorbital and postfrontal, or postorbital and jugal. Arnold^28^.

80. Postorbital firmly sutured to skull roofing bones (postfrontal or parietal): (0) present; (1) postorbital barely underlaps parietal at frontoparietal suture (equals mobile joint of Arnold 1998); (2) postorbital tapers to blunt tip separated from parietal. Smith^24,26^

(ordered).

81. Postorbital-ectopterygoid contact: (0) absent; (1) present. Moody^12^.

82. Postorbital jugal ramus: (0) extends ventral to quadrate head; (1) level with quadrate head; (2) or above quadrate head. Estes *et al.*^20^. (ordered).

83. Postorbital-jugal suture: (0) long, firm, immobile, tonque-in-groove suture, with jugal largely ventrolateral to postorbital; (1) short abutting suture, with jugal reduced to tab-like dorsal tip that lies distal to postorbital; (2) jugal tapers smoothly to apex, which is loosely joined to lateral face of postorbital via connective tissue; (3) postorbital with process extending lateral to tapering apex of jugal. Gauthier *et al*.^4^. (ordered).

84. Postorbital contribution to posterior orbital margin: (0) less than 39%; (1) 39-52%; (2) 53-66%; (3) 67-80%; (4) more than 80%. Lee and Caldwell^29^ (ordered).

85. Postorbital spreads onto dorsal surface of postfrontal: (0) absent; (1) present.

86. Postorbital dorsal part, above lateral wing of parietal: (0) uniformly narrow; (1) broadened. Kluge^18^.

87. Postorbital extent posteriorly: (0) to end of parietal table or less; (1) posterior

to parietal table. Gauthier *et al*.^4^.

88. Parietal fusion: (0) paired; (1) fused. Gauthier, Estes and de Queiroz^10^.

89. Parietal ventral lappet: (0) poorly developed or absent; (1) prominent V-shaped, flat process. Estes *et al.*^20^.

90. Parietal temporal muscles originate: (0) dorsally on parietal table and supratemporal process of parietal; (1) ventrally on parietal table and dorsally on supratemporal process; (2) ventrally on parietal table and supratemporal process. Gauthier^13^ (ordered).

91. Parietal temporal fossa shape: (0) temporal muscles originate dorsally across entire parietal table all the way to frontal anteriorly (at least laterally); (1) anterolateral corner of temporal fossa terminates posteriorly, dorsal and ventral margins of temporal fossa converge behind frontal, so parietal table extends as flat surface toward orbital margin, and temporal

muscles are confined laterally. Gauthier *et al*.^4^.

92. Parietal, middle third: (0) narrow in dorsal view; (1) wide in dorsal view. Kluge^18^.

93. Parietal sagittal crest: (0) absent; (1) present; (2) projecting dorsally. Etheridge and de Queiroz^30^ (ordered).

94. Parietal nuchal fossa width: (0) narrow; (1) wide; (2) overgrown by parietal (nearly) to midline. Gauthier *et al*.^4^.

95. Parietal postparietal projection near midline (bifid distally or not; see character 97): (0) absent; (1) present. Gauthier *et al*.^4^.

96. Parietal-supraoccipital contact: (0) absent; (1) parietal overlaps supraoccipital on midline;

(2) abuts supraoccipital on midline; (3) dorsoventral parasagittal abutment; (4) supraoccipital around processus ascendens tectum synoticum forms stout, flat-topped pedicle that abuts parietal posteroventromedially. Lee and Scanlon^31^.

97. Parietal bifid supraoccipital process: (0) absent; (1) present; (2) clasping supraoccipital crest. Lang^32^ (1991) (ordered).

98. Parietal descending lamina articulates with supraoccipital ascending lamina: (0) absent, parietal descending lamina is anterior to supraoccipital ascending lamina; (1) present, parietal descending lamina is posterior to the supraoccipital ascending lamina. Gauthier *et al*.^4^.

99. Parietal extent over braincase in dorsal view: (0) does not cover occiput; (1) covers nearly all of occiput; (2) with emarginate lateral fossae, Lang^32^. Estes *et al.*^20^ (ordered).

100. Parietal posterior margin, in dorsal view: (0) does not form an elongate, slender and pointed posterior process; (1) does form an elongate, slender and pointed posterior

process. Kluge^18^.

101. Parietal supratemporal process length: (0) well-developed; (1) reduced, less than 25% of parietal width; (2) absent, Tchernov *et al*.^33^. Estes *et al.*^20^ (ordered).

102. Parietal supratemporal process orientation: (0) directed laterally; (1) directed posterolaterally; (2) directed posteriorly. Gauthier *et al*.^4^. (ordered).

103. Parietal contribution to back of the upper temporal fenestra: (0) short supratemporal process, parietal only forms about half of the upper temporal fenestra posterior arch, with supratemporal forming distal half; (1) long parietal supratemporal process extends distally to near the quadrate head. Gauthier *et al*.^4^.

104. Parietal foramen: (0) present; (1) absent. Estes *et al.*^20^.

105. Parietal foramen position: (0) in parietal; (1) at frontoparietal suture; (2) in frontal. Estes *et al.*^20^. (ordered).

106. Parietal supraorbital process: (0) absent; (1) present; (2) deeply clasping frontal orbital margin. Tchernov *et al*.^33^ (ordered).

107. Parietal postorbital process: (0) absent; parietal barely, if at all, laps behind postorbital apex in horizontal section; (1) parietal vertically oriented lappet extends laterally to overlap

postorbital to form anteromedial margin of upper temporal fenestra. Gauthier *et al*.^4^.

108. Parietal epipterygoid process: (0) absent; (1) distinct process; (2) reaches alar process of

prootic. Estes *et al.*^20^. (ordered).

109. Parietal-prootic contact: (0) absent; (1) contact at apex of alar process; (2) extensive conformable contact, with parietal overlapping prootic laterally throughout length; (3) discrete ventral process of parietal overlaps prootic alar process laterally. Lee^29^.

110. Parietal ventral triangular downgrowths of temporal muscle origin overlap prootic laterally, with latter abutting former medially, just anterior to supraoccipital: (0) absent;

(1) present. Gauthier *et al*.^4^.

111. Maxilla (post-) premaxillary process contact: (0) not in contact; (1) in contact, or nearly so, but always excluding premaxilla from vomer dorsally; (2) in contact and vertically expanded. Estes *et al.*^20^.

112. Maxilla premaxillary process dorsal surface grooved (often enclosed) for passage of a deeper and more internally placed ramus of the subnarial artery: (0) absent; (1) present. Smith^24,26^.

113. Maxilla and vomer: (0) do not meet at anterior margin of fenestra exochoanalis; (1) meet at anterior margin of fenestra exochoanalis. Rieppel *et al.*^34^.

114. Maxilla facial process length/maxilla length: (0) 10-15%; (1) 16-23%; (2) 25-36%; (3) 38-55%; (4) more than 56%. Gauthier^13^ (ordered).

115. Maxilla facial process height: (0) tall, to skull roof; (1) reduced; (2) absent; (3) columnar process received in longitudinal concavity on anterior face of prefrontal (ordered).

116. Maxilla facial process apical surface faces: (0) laterally; (1) dorsolaterally; (2) anterodorsally; (3) large, triangular, dorsally directed surface sharply set off from nearly vertical external surface of facial process. Smith^24^.

117. Maxilla facial process medial face with a posterodorsally trending ridge demarcating the anterior limits of a shallow, oval fossa—the naso-lacrimal fossa—bordered by the lacrimal and infraorbital canals posteriorly: (0) absent; (1) present. Gauthier^13^.

118. Maxilla narial margin rises at: (0) high angle; (1) low angle. Pregill *et al*.^19^.

119. Maxilla firmly sutured to palatine: (0) present; (1) prominent palatine process of maxilla; (2) loosely ligamentous connection via projecting palatine process of maxilla and distinct maxillary process of palatine, with the former lying anterior to the latter; (3) maxilla

free of palatine, suspended from prefrontal; (4) maxilla rotates to erect fang, Kardong^35^. Cundall *et al*.^17^.

120. Maxilla suborbital ramus extends posteriorly: (0) to roughly midorbit (or anterior); (1) to posterior quarter of orbit; (2) to posterior edge of orbit; (3) posterior to orbit (or frontoparietal suture). Lee and Scanlon^31^ (ordered).

121. Maxilla suborbital process width ventral to ectopterygoid: (0) tapers posteriorly; (1) widens below articulation (i.e., ectopterygoid flange). Kluge^36^.

122. Jugal depth below orbit: (0) jugal suborbital ramus not much deeper dorsoventrally below mid-orbit than postorbital ramus is wide anteroposteriorly; (1) jugal very deep below orbit. Gauthier *et al*.^4^.

123. Maxilla suborbital process tip shape at jugal articulation: (0) suborbital margin slopes smoothly to tip; (1) with distinct step or V-shaped notch distally at jugal articulation. Gauthier *et al*.^4^.

124. Maxilla posterior process shortens: (0) to midorbit or longer; (1) to anterior half of oribit. Gauthier *et al*.^4^.

125. Maxilla, intramaxillary joint: (0) absent; (1) present. Cundall and Irish^37^.

126. Prefrontal: (0) present; (1) reduced; (2) absent. Kearney^38^ (ordered).

127. Prefrontal broadly overlaps frontal posterodorsally: (0) absent; (1) present. Gauthier *et al*.^4^.

128. Prefrontal orbitonasal margin: (0) slopes ventrolaterally; (1) vertical; (2) slopes ventromedially; (3) extends beneath subolfactory processes; (4) extends to

near contact with its opposite on midline. Gauthier *et al*.^4^. (ordered).

129. Prefrontal posterior extent along orbital margin: (0) terminates in anterior half of orbit; (1) extends to midorbit; (2) extends posterior to midorbit, Lee *et al.*^39^. Estes *et al.*^20^. (ordered).

130. Prefrontal boss: (0) absent; (1) present; (2) in projecting canthal crest. Gauthier^40^ (ordered).

131. Prefrontal nasolacrimal cornu: (0) absent; (1) present. Gauthier *et al*.^4^.

132. Prefrontal medial extent across anterior margin of frontal: (0) prefrontal extends 50% or less across frontal anterior width; (1) extends 50% to 65% across frontal anterior width; (2) extends 65% to 75% across frontal anterior width; (3) extends 85% or more across frontal to approach midline. Cundall *et al*.^17^ (ordered).

133. Prefrontal-frontal suture in cross-section: (0) prefrontal arcs gently about anterolateral frontal margin along entire anteroposterior length; (1) prefrontal strongly bifid, clasps frontal posteriorly then spreads dorsally and reduced ventrally anteriorly; (2) frontal clasps prefrontal

in V-shaped notch. Gauthier *et al*.^4^. (ordered).

134. Prefrontal length relative to height: (0) long anteroposteriorly; (1) short anteroposteriorly. Gauthier *et al*.^4^.

135. Prefrontal-maxilla articulation: (0) prefrontal posteroventromedial corner narrowly (or not at all) in contact with maxilla lateral to palatine; (1) prefrontal broadly contacts maxilla supradental shelf lateral to palatine; (2) prefrontal has mobile contact with maxilla; (3) rod-like prefrontal arched dorsally, bifid at each end, with mobile joints at maxilla and frontal (prefrontal functionally part of upper jaw). Tchernov et al.^33^.

136. Prefrontal arcs about orbitonasal fenestra, with posteroventromedial corner curving inwards toward palatine in cross-section: (0) absent; (1) present. Gauthier *et al*.^4^.

137. Lacrimal: (0) present; (1) absent. Estes *et al.*^20^.

138. Lacrimal position relative to lacrimal duct: (0) lacrimal with broad exposure laterally, reaching from lateral floor of lacrimal duct up the medial face of the maxilla to contact a lateral process of the prefrontal that roofs the lacrimal duct in cross section; (1) lacrimal arches over the lacrimal duct to replace the prefrontal dorsally, broadly floors the lacrimal duct with a medial process posteriorly passing up the lateral face of the prefrontal; (2) lacrimal reduced to floor of lacrimal duct and lingual surface of maxilla, and barely, if at all, exposed laterally; (3) lacrimal bone reduced ventrally, confined mainly to dorsolateral corner

of lacrimal duct. The complexity of lacrimal morphology is difficult to illustrate from any single view. Gauthier *et al*.^4^.

139. Lacrimal foramen size: (0) small; (1) large. Moody^12^.

140. Lacrimal foramen number: (0) one; (1) divided on orbital surface; (2) divided through to olfactory surface. Pregill *et al*.^19^. (ordered).

141. Lacrimal duct position: (0) between prefrontal and lacrimal; (1) enclosed in prefrontal, except ventrally; (2) enclosed entirely in prefrontal. Lee29 (ordered).

142. Jugal: (0) present; (1) absent. Estes *et al.*^20^.

143. Jugal extent anteriorly with respect to tooth row: (0) jugal broadly overlaps level of posterior maxillary tooth row; (1) jugal overlaps the most posterior maxillary tooth; (2) jugal

just reaches base of, or stops short of, the most posterior maxillary tooth. Gauthier *et al*.^4^.

(ordered).

144. Jugal anterior extent: (0) broadly separated from prefrontal; (1) reaches level of prefrontal. Gauthier *et al*.^4^.

145. Jugal-lacrimal overlap: (0) jugal lateral to lacrimal; (1) jugal medial to lacrimal; (2) jugal ventral to lacrimal. Gauthier *et al*.^4^.

146. Jugal articulation with maxilla in cross-section: (0) rounded ventral margin of jugal and shallow and more rounded contour of the maxilla supradental shelf; (1) acute ventral margin of jugal lies in narrow longitudinal groove on dorsal surface of maxillary supradental shelf. Gauthier *et al*.^4^.

147. Jugal lateral extent over maxilla in cross-section: (0) maxilla suborbital border wraps dorsally around jugal external margin; (1) jugal laps over external suborbital margin. Gauthier *et al*.^4^.

148. Jugal with inverted V-shaped notch clasping suborbital edge of maxilla: (0) absent; (1) present. Gauthier *et al*.^4^.

149. Jugal lateral exposure below orbit: (0) absent; (1) partly exposed above orbital margin of maxilla; (2) entirely exposed above orbital margin of maxilla. Estes *et al.*^20^. (ordered).

150. Jugal suborbital ramus: (0) shallow; (1) deep. Gauthier *et al*.^4^.

151. Jugal suborbital boss: (0) absent; (1) present. Borsuk-Białynicka and Moody^41^.

152. Jugal postorbital ramus development: (0) complete bony postorbital bar; (1) incomplete bony postorbital bar; (2) bony postorbital bar absent. Estes *et al.*^20^. (ordered).

153. Jugal postorbital ramus shape in lateral outline: (0) narrow; (1) wide. Gauthier^13^.

154. Jugal contacts squamosal: (0) present; (1) absent, Gauthier^13^; (2) broad contact. Gauthier *et al*.^4^.

155. Jugal posterior process: (0) complete lower temporal bar; (1) reduced to a discrete bony posterior process, Gauthier, Estes and de Queiroz^10^; (2) absent. Benton^42^.

156. Jugal posterior process orientation: (0) more posterior in orientation; (1) more ventral in orientation. Gauthier *et al*.^4^.

157. Jugal medial ridge: (0) medial ridge weak, jugal lateral to ectopterygoid at base in dorsal view; (1) medial ridge pronounced, base of medial ridge projects behind

ectopterygoid base in dorsal view. Gauthier *et al*.^4^.

158. Jugal cross-section at level of ectopterygoid: (0) subtriangular; (1) depressed. Gauthier *et al*.^4^.

159. Squamosal: present (0), absent (1). Estes *et al.*^20^.

160. Squamosal length relative to epipterygoid position: (0) squamosal does not extend anterior to level of epipterygoid; (1) squamosal extends anterior to level of epipterygoid. Gauthier *et al*.^4^.

161. Squamosal temporal ramus-parietal contact: (0) temporal ramus diverges from parietal supratemporal process; (1) temporal ramus broadly contacts parietal supratemporal

process. Gauthier *et al*.^4^.

162. Squamosal base of temporal ramus: (0) diverges from parietal; (1) base lies against parietal. Gauthier *et al*.^4^.

163. Squamosal temporal ramus width: (0) slender; (1) widens posteriorly; (2) widens anteriorly with medial shelf along parietal that roofs posterior end of upper temporal

fenestra. Gauthier *et al*.^4^.

164. Squamosal temporal ramus shape: (0) compressed; (1) depressed. Gauthier *et al*.^4^.

165. Squamosal ascending process: (0) present; (1) absent. Gauthier^13^.

166. Supratemporal: (0) present; (1) absent. Estes *et al.*^20^.

167. Supratemporal shortens: (0) supratemporal longer than squamosal-parietal contact; (1) supratemporal shorter than squamosal-parietal contact; (2) supratemporal very

small. Gauthier *et al*.^4^. (ordered).

168. Supratemporal lengthens: (0) posterior to level of parietal notch; (1) near to level of parietal notch; (2) anterior to level of parietal notch. Pregill et al.^19^ (ordered).

169. Supratemporal anterior suture with parietal shape: (0) supratemporal lies flat against supratemporal process of parietal; (1) inserts in slot in supratemporal process of parietal. Frost and Etheridge^43^.

170. Supratemporal position on parietal: (0) partly ventral; (1) partly ventrolateral; (2) all lateral, Etheridge & de Queiroz (1988); (3) dorsolateral (on either parietal or braincase alone). de Queiroz^44^.

171. Supratemporal anterior terminus: (0) posterior to level of trigeminal nerve exit; (1) anterior to level of trigeminal nerve exit. Tchernov *et al*.^33^.

172. Supratemporal anterior extent in snakes without supratemporal process of parietal: (0) supratemporal does not extend anterior of posterior border of parietal table; (1) supratemporal extends anterior of posterior border of parietal table. Gauthier *et al*.^4^.

173. Supratemporal orientation: (0) anterior to quadrate head; (1) dorsal to quadrate head. Rieppel^45^.

174. Supratemporal free posteriorly: (0) supratemporal ends near attachment to braincase; (1) extends freely posterior to otoccipital. Kluge^36^.

175. Supratemporal hidden in dorsal view: (0) supratemporal at least partly exposed dorsally on lateral side of parietal supratemporal process; (1) slender and hidden completely

from view by parietal-squamosal contact dorsally. Gauthier *et al*.^4^.

176. Supratemporal posterior exposure on parietal supratemporal process: (0) narrow or absent; (1) present broadly. Etheridge & de Queiroz^30^.

177. Quadrate head attachment: (0) tapering peg-like head loosely attached in socket formed largely by squamosal; (1) quadrate head pivots on slender tapering tip of squamosal (Gauthier 1984); (2) bluntly abuts supratemporal and squamosal. Gauthier *et al*.^4^.

178. Quadrate head suspension: (0) supratemporal and squamosal separate quadrate head from braincase (except narrowly beneath tip of supratemporal); (1) quadrate head abuts braincase ventral to supratemporal; (2) quadrate head broadly contacts braincase anteriorly.

Gauthier *et al*.^4^.

179. Quadrate suprastapedial process: (0) absent; (1) present. Lee^14^.

180. Quadrate lateral conch: (0) present; (1) absent. Benton^42^.

181. Quadratojugal: (0) present; (1) absent. Benton^42^.

182. Quadrate-pterygoid overlap: (0) extensive; (1) short overlap or small lappet,

Gauthier^40^; (2) very narrow overlap or lappet absent, Benton^42^; (3) no overlap, ligamentous connection only. Gauthier *et al*.^4^. (ordered).

183. Quadrate accessory process arising off anteromedial edge near quadrate head abuts braincase in region of horizontal semicircular canal: (0) absent; (1) present. Gauthier *et al*.^4^.

184. Quadrate stylohyal process on medial face of quadrate: (0) absent; (1) present as oval disc; (2) present as narrow cylindrical ridge. Rieppel^46^ (ordered).

185. Quadrate height to braincase depth ratio (braincase depth measured from near the quadrate head): (0) less than 50%; (1) 50–59%; (2) 60–69%; (3) 70–79%; (4) more than

79%. Gauthier *et al*.^4^. (ordered).

186. Quadrate “pythonomorph”: (0) bowed more or less, but not in both lateral and posterior views; (1) massive, and strongly bowed anteriorly in lateral view and laterally in posterior view, throughout length, and with prominent ventrally-directed suprastapedial process forming cavum tympani. DeBraga and Carroll^47^.

187. Quadrate foramen size: (0) large; (1) small; (2) tiny. Gauthier *et al*.^4^. (ordered).

188. Quadrate slopes anteroventrally (more than 90° equals anterior slope from quadrate head): (0) vertical to posterior slope; (1) 94–107°; (2) 108–121°; (3) 122–135°; (4) more

than or equal to 136°. Gauthier *et al*.^4^. (ordered).

189. Quadrate slopes posteroventrally (less than 90° equals posterior slope from quadrate head): (0) vertical to anterior slope (87–93°); (1) 86–68°; (2) 67–49°; (3) 48–31°; (4)

less than 30°. Kluge^18^ (ordered).

190. Stapes: (0) imperforate; (1) perforate. Gauthier, Estes and de Queiroz^10^.

191. Stapedial shaft: (0) long and slender; (1) short and thick. Lee^29^.

192. Stapedial footplate: (0) small; (1) large. Lee^29^.

193. Stapedial footplate: (0) does not fill fenestra ovalis; (1) fills fenestra ovalis. Lee^29^.

194. Fenestra ovalis orientation: (0) opens directly laterally; (1) opens anterolaterally; (2) opens ventrolaterally; (3) opens posterolaterally. Gauthier, Estes and de Queiroz^10^.

195. Extracolumella: (0) present; (1) absent. Rieppel^11^.

196. Septomaxilla: (0) present; (1) absent. Estes *et al.*^20^.

197. Septomaxilla dorsolateral contacts: (0) no contacts; (1) abuts laterally with prefrontal and nasal; (2) abuts laterally with nasal only; (3) abuts laterally with prefrontal only. Gauthier *et al*.^4^.

198. Anterior end of septomaxilla: (0) meets maxilla in immovable joint; (1) mobile, septomaxilla not contacting maxilla. Lee^29^.

199. Septomaxilla position relative to vomeronasal organ: (0) occupies a lateral position, not contributing to nasal cavity or to roofing of vomeronasal organ; (1) occupies a more medial position, contributing to nasal cavity and roofing vomeronasal organ. Gauthier, Estes and de Queiroz^10^.

200. Septomaxilla, dorsal expansion: (0) flat or weakly convex, vomeronasal organ small; (1) expanded and convex, reflecting large size of vomeronasal organ. Gauthier, Estes and de Queiroz^10^.

201. Septomaxilla: (0) does not contact the dorsal surface of the palatal shelf of the maxilla; (1) contacts the dorsal surface of the palatal shelf of the maxilla. Lee^29^.

202. Septomaxilla divides vomeronasal organ: (0) absent; (1) present. Gauthier *et al*.^4^.

203. Septomaxilla medial flange produced into a long, posterodorsally directed, blade-like process: (0) absent; (1) present, but nasal intercedes between septomaxilla and frontal; (2) extends to frontal beneath nasal, Tchernov et al. (2000); (3) develops an expanded faceted articulation with the frontal. Caldwell^48^ (ordered).

204. Septomaxilla lateral flange: (0) absent; (1) present; (2) reaches well above roof of vomeronasal organ. Lee^49^ (ordered).

205. Septomaxilla medial flange: (0) absent; (1) present. Estes *et al.*^20^.

206. Septomaxilla, posterior extent of medial flange: (0) short, not reaching level of prefrontal; (1) long, extends posteriorly to anteroposterior level of anterior margin of

prefrontal. Lee^29^.

207. Septomaxilla posterior process on laterally ascending flange: (0) short or absent; (1) long, extends posteriorly deep to prefrontals. Caldwell^48^.

208. Nervus ethmoidalis medialis: (0) above septomaxilla; (1) enclosed in septomaxilla anteriorly; (2) in anterior half of septomaxilla; (3) enclosed posteriorly in septomaxilla. Gauthier *et al*.^4^. (ordered).

209. Vomeronasal organ, concha: (0) simple diverticulum of nasal capsule; (1) completely separated from nasal capsule, with fungiform body. Gauthier, Estes and de Queiroz^10^.

210. Vomeronasal organ, cupola: (0) fenestrated medially, even if only narrowly; (1) closed medially. Cundall et al.^17^.

211. Vomeronasal organ and mushroom body: (0) not fully enclosed by septomaxilla and vomer; (1) fully enclosed by septomaxilla and vomer only. Estes *et al.*^20^.

212. Vomer fusion: (0) absent; (1) partial; (2) fully fused. Estes *et al.*^20^.

213. Vomer size: (0) vomer extends backwards no further than anteriormost contact of palatine with maxilla; (1) vomer extends backwards beyond anteriormost contact of palatine with maxilla. Estes *et al.*^20^.

214. Vomer: (0) main portion plate-like; (1) main portion rodlike. Lee^29^.

215. Vomer overlaps (dorsally) the palatal shelf of the maxilla behind posterior margin of opening of vomeronasal organ: (0) absent; (1) present. Gauthier, Estes and de Queiroz^10^.

216. Vomer: (0) does not establish any sutural contact with the palatal shelf of the maxilla behind the incisura Jacobsoni; (1) establishes narrow contact with the palatal shelf of the

maxilla behind the incisura Jacobsoni; (2) establishes broad contact with the palatal shelf of the maxilla along the entire length of the lateral margin of vomer. Rieppel *et al.*^34^. (ordered).

217. Vomer to vomeronasal organ relation: (0) vomer ventral to vomeronasal organ; (1) encapsulates vomeronasal organ posteriorly and medially; (2) with margins

enclosing posterior wall sloping ventrolaterally; (3) further expanded laterally to completely encapsulate vomeronasal organ posteriorly. Rieppel *et al.*^34^. (ordered).

218. Vomer meets septomaxilla: (0) at posterior margin of opening of vomeronasal organ; (1) at lateral margin of opening of vomeronasal organ. Tchernov *et al*.^33^.

219. Margin of vomer at opening of vomeronasal organ: (0) flat; (1) curled downwards. Gauthier *et al*.^4^.

220. Vomeronasal nerve exit: (0) dorsal to vomer; (1) via canals dorsally on vomer; (2) via foramen at back end of vomer; (3) via sieve-like arrangement of foramina through back end of vomer. Rieppel *et al.*^34^ (ordered).

221. Vomer degree underlap of palatine: (0) just at tips; (1) extending posteriorly to level of maxillaectopterygoid first contact. Kearney^38^.

222. Vomer ventral longitudinal ridges: (0) absent; (1) long and converge toward midline, well-developed below vomeronasal nerve exit from septomaxilla; (2) short parasagittal ridges anteriorly on vomer at level of vomeronasal duct opening; (3) discrete parasagittal canals anteriorly on vomer delimited by lateral and median ridges. Gauthier *et al*.^4^.

223. Vomer septum transversely fenestrate: (0) absent; (1) present; (2) at posteroventral corner of vomer septum. Gauthier *et al*.^4^. (ordered).

224. Vomer septum (vertical lamina) height: (0) low, not forming septum; (1) partly separating olfactory chambers; (2) nearly completely separating olfactory chambers

along with septomaxilla and nasal; (3) only ventral edge of septum remains; (4) V-shaped notch separates dorsal and ventral rami of vomer septum. Gauthier *et al*.^4^.

225. Vomer, posterodorsal margin forms expanded hollow flange: (0) absent; (1) present. Gauthier *et al*.^4^.

226. Vomer, transverse flange rises vertically to meet septomaxilla and encloses vomeronasal organ posteriorly: (0) absent; (1) present Gauthier *et al*.^4^.

227. Vomer contact with subolfactory process of frontal: (0) rufescens, oblique ventral view of palate). absent; (1) present. Rieppel^22^.

228. Vomer, descending tubercle (or ridge) at vomero-palatine junction: (0) absent; (1) tubercle present; (2) ridge/tubercle present on vomer and/or adjacent palatine. Gauthier *et al*.^4^.

229. Vomer, foramina on palatal surface near midline: (0) paired; (1) single. Gauthier *et al*.^4^.

230. Vomer, teeth: (0) present; (1) absent. Gauthier, Estes and de Queiroz^10^.

231. Palatines: (0) separated; (1) anterior contact only; (2) contact extends to midpoint,

or beyond. Lee^14^ (ordered).

232. Palatine relative to maxilla-lacrimal-jugal articulation: (0) palatine sits medial to lacrimal and/or jugal and maxilla in cross section; (1) palatine inserts between lacrimal and/or jugal and maxilla in cross section. Gauthier *et al*.^4^.

233. Palatine dorsal canal: (0) shallow longitudinal sulcus; (1) upturned lateral and medial edges of palatine demarcate deep, narrow canal ending in enclosed fossa. Gauthier *et al*.^4^.

234. Palatine, vomerine process dorsally on vomer: (0) vomer attaches over entire face of vomerine process of palatine; (1) narrow slender tip of palatine loosely attached

to vomer, Rieppel et al. (2008); (2) long slender palatine process clasped in groove on dorsal surface of vomer; (3) ventral edge of vomerine process of palatine attached ligamentously between bifid palatine process of vomer, Cundall *et al*.^17^.

235. Palatine, vomerine process buttresses vomer: (0) palatine vomerine process tapers anteromedially; (1) splays laterally at tips to buttress vomer posteriorly. Gauthier *et al*.^4^.

236. Palatine, vomerine process passes vomer: (0) medial to vomer tines; (1) lateral to vomer tines (posteromedial process of vomer). Gauthier *et al*.^4^.

237. Ventral projections from anterior end of palatine, near palatine-vomer suture: (0) absent; (1) present. Gauthier *et al*.^4^.

238. Ventromedial extension from maxillary process of palatine (choanal processes of palatine): (0) present but not descending ventromedially; (1) present and descending ventromedially to reach in between (or close to) posterior tips of vomers; (2) absent. Cundall *et al*.^17^. (ordered).

239. Maxillary process of palatine: (0) is situated anterior to the posterior end of palatine; (1) is situated at the posterior end of the palatine. Tchernov *et al*.^33^.

240. Palatine contribution to suborbital fenestra: (0) reduced posteromedially, and pterygoid broadly exposed in suborbital fenestra; (1) palatine extends posteriorly along lateral edge

of pterygoid so that pterygoid narrowly enters suborbital fenestra; (2) palatine fully excludes pterygoid from border of suborbital fenestra. Gauthier *et al*.^4^.

241. Palatine-pterygoid overlap: (0) palatine overlaps pterygoid at tip and ectopterygoid near base, otherwise lateral in position; (1) palatine overlaps pterygoid dorsally from lateral to near medial margin of pterygoid, with loose abutment laterally; (2) palatine barely overlaps pterygoid laterally and pterygoid does not extend well anterior to ectopterygoid-jugal-maxilla juncture; (3) palatine barely overlaps pterygoid, joint nearly transverse; (4) complex

pattern of clasping projections. Cundall *et al*.^17^.

242. Palatine anterior “dentigerous” process: (0) absent; (1) present only as short extension of palatine anterior to maxillary process, Lee and Scanlon^31^; (2) present (with teeth). Cundall *et al.*^17^. (ordered).

243. Palatine dentigerous process reduction: (0) long, bearing six or more teeth; (1) short, bearing five or fewer teeth. Gauthier *et al*.^4^.

244. Infraorbital canal divides anteriorly in palatine: (0) single foramen anteriorly; (1) double anteriorly, with medial palatine ramus small and lateral large. Gauthier *et al*.^4^.

245. Infraorbital canal position: (0) lateral, between palatine and dorsal surface of supradental shelf of maxilla; (1) medial, entirely in palatine. de Queiroz^44^.

246. Palatine foramen: (0) absent; (1) present, enters palatine dorsally toward its anterior end to pass anteroventrolaterally into the infraorbital canal. Gauthier *et al*.^4^.

247. Palatine, choanal process: (0) curves medially and meets the vomer in a well-defined articular facet; (1) touches or abuts the vomer without articulation, or remains separated from vomer. Tchernov *et al.*^33^.

248. Palatine, choanal process: (0) forms an extensive concave surface dorsal to the ductus nasopharyngeus; (0) narrows to form a curved finger-like process; (2) forms a short vertical or horizontal lamina that does not reach the vomer. Tchernov *et al*.^33^ (ordered).

249. Palatine: (0) simplicipalatinate; (1) incipient duplicipalatinate; (2) intermediate; (3) fully duplicipalatinate. Rieppel *et al.*^34^.

250. Palatine choanal fossa development: (0) absent; (1) present anteriorly on palatine; (2) extending about half way back on palatine; (3) fully developed to end of element. Gauthier *et al*.^4^. (ordered).

251. Palatine, subchoanal process medial edge shape in ventral view: (0) present only on anterior one-third of palatine; (1) roughly arcuate; (2) roughly parasagittal. Gauthier *et al*.^4^. (ordered).

252. Palatine, posterior emargination of anterodorsal margin of choanal fossa: (0) anterior to anteroposterior midpoint of palatine-maxilla suture; (1) extends posterior to anteroposterior

midpoint of palatine-maxilla suture. Gauthier *et al*.^4^.

253. Posteromedial process of palatine: (0) long, overlaps at least two pterygoid teeth; (1) short, overlaps no more than one pterygoid tooth. Kluge^18^.

254. Palatine, shape of posterolateral margin at pterygoid suture: (0) unmodified; (1) palatine with discrete surface set off from choanal fold, extending along lateral margin from

maxillary to pterygoid sutures; (2) transversely broad palatine at pterygo-palatine suture strongly restricts suborbital fenestra. Gauthier *et al*.^4^.

255. Palatine teeth: (0) present; (1) absent. Gauthier^13^.

256. Palatine teeth size: (0) small conical denticles; (1) enlarged, but smaller than marginal teeth; (2) highly enlarged, similar in size to marginal teeth. Lee^31^ (ordered).

257. Pterygoids: (0) contacting each other; (1) palatal rami fully separated. Estes *et al.*^20^.

258. Pterygoid separation on midline: (0) pterygoids narrowly separated for most of their length; (1) broad at base, narrow anteriorly; (2) broad at base, but not as narrowly separated anteriorly; (3) broad throughout length. Estes *et al.*^20^. (ordered).

259. Pterygoid, palatine ramus: (0) contacts vomer; (1) does not contact vomer. Gauthier, Estes and de Queiroz^10^.

260. Pterygoid-palatine joint; length of complex pattern of projections in snakes: (0) long; (1) medium; (2) short. Rieppel *et al*.^50^ (ordered).

261. Pterygoid, palatine ramus clasps pterygoid ramus of palatine: (0) absent; (1) present. Wu *et al.*^27^.

262. Pterygoid transverse process and ectopterygoid (pterygoideus muscle insertion) nearly as deep as mandible (at least 80% mandible depth), and ectopterygoid transversely broad, covering most of transverse process of pterygoid in anterior view: (0) absent; (1) present. Gauthier *et al*.^4^.

263. Pterygoid posterior extent: (0) pterygoid does not reach level of occipital condyle; (1) pterygoid reaches level of occipital condyle; (2) pterygoid reaches well posterior to level

of occipital condyle. Gauthier *et al*.^4^. (ordered).

264. Pterygoid, quadrate ramus short and small, tightly wrapping around posteromedial (ventromedial if quadrate horizontally oriented) surface of quadrate: (0) absent; (1)

present. Wu et al.^27^.

265. Pterygoid, quadrate ramus: (0) robust, rounded or triangular in cross-section, but without groove; (1) blade-like and with distinct longitudinal groove for the insertion of the

protractor pterygoidei muscle. Tchernov *et al.*33.

266. Pterygoid, ventral flange (“wing-shaped extension”) of quadrate ramus: (0) absent; (1) present. Gauthier *et al*.^4^.

267. Pterygoid teeth: (0) present; (1) absent. Pregill *et al.*^19^.

268. Pterygoid teeth: (0) small conical denticles; (1) enlarged, but smaller than marginal teeth; (2) highly enlarged, similar in size to marginal teeth. Lee^31^. (ordered).

269. Pterygoid teeth: (0) restricted to palatal ramus of pterygoid; (1) extend posteriorly onto quadrate ramus of pterygoid. Mahler and Kearney^51^.

270. Ectopterygoid: (0) present; (1) absent. Gauthier *et al*.^4^.

271. Ectopterygoid size and restriction of suborbital fenestra: (0) ectopterygoid relatively slender, fenestra widely open; (1) ectopterygoid enlarged medially, restricting suborbital fenestra; (2) ectopterygoid highly enlarged medially, closing suborbital fenestra. Estes *et al.*^20^. (ordered).

272. Ectopterygoid angulation in dorsal view: (0) nearly orthogonal; (1) obtuse angle (including crescentic curve). Smith^26^.

273. Ectopterygoid anterior length: (0) well separated from palatine above maxilla; (1) near to or in contact with palatine. Estes *et al.*^20^.

274. Anterior end of ectopterygoid: (0) restricted to posteromedial edge of maxilla; (1) located dorsal to maxilla, invading the dorsal surface of the maxilla to variable degrees. Tchernov *et al.*^33^.

275. Ectopterygoid-maxilla suture: (0) ectopterygoid lies dorsally along supradental shelf of maxilla; (1) ectopterygoid abuts posteromedial corner of maxilla; (2) ectopterygoid with slot laterally clasping maxilla; (3) ectopterygoid overlapping maxilla more ventrally than dorsally; (4) interdigitating suture, with maxilla at least partly overlapping ectopterygoid dorsally. Smith^26^.

276. Ectopterygoid maxillary process shape in dorsal view: (0) tapers or parallel-sided; (1) widens anteriorly; (2) to more than three times wider anteriorly relative to ectopterygoid shaft.

Gauthier et al. (2012) (ordered).

277. Ectopterygoid maxillary process anterior notch: (0) tapers forward of maxilla contact; (1) notched anteriorly; (2) with large, rectangular, lateral ramus produced directly laterally. Gauthier *et al*.^4^. (ordered).

278. Ectopterygoid-maxilla posterior process suture: (0) ectopterygoid medial and mainly dorsal to maxilla; (1) ectopterygoid abuts maxilla on posteromedial edge only; (2) ectopterygoid contacts jugal only. Gauthier *et al*.^4^.

279. Lateral edge of maxillary ramus of ectopterygoid: (0) slopes medially; (1) straight. Tchernov *et al*.^33^.

280. Ectopterygoid, prefrontal and palatine relations: (0) ectopterygoid does not underlap palatine posteriorly below prefrontal; (1) ectopterygoid underlaps palatine below prefrontal. Gauthier *et al*.^4^.

281. Ectopterygoid-palatine ventral articulation: (0) palatine-maxilla contact excludes ectopterygoid; (1) ectopterygoid anterior process largely separates palatine from maxilla posteriorly. Gauthier *et al*.^4^.

282. Ectopterygoid hooked posterior process flat and exposed dorsally, ventrally and laterally: (0) absent; (1) present. Gauthier *et al*.^4^.

283. Ectopterygoid posterior process: (0) prominent; (1) small lateral knob; (2) absent (ordered). Smith^26^.

284. Ectopterygoid posterior process lengthens: (0) does not extend past coronoid apex; (1) extends past coronoid apex. Gauthier *et al*.^4^.

285. Ectopterygoid dorsal process height: (0) tall; (1) short; (2) absent (not extending up medial face of jugal). Gauthier *et al*.^4^. (ordered).

286. Ectopterygoid: (0) does not contact prefrontal; (1) contacts prefrontal at base of orbit. Gauthier *et al*.^4^.

287. Ectopterygoid-pterygoid contact: (0) predominantly dorsal; (1) predominantly lateral. Tchernov *et al.*^33^.

288. Ectopterygoid pterygoid process length: (0) short; (1) longer, but still anterior to trigeminal foramen; (2) longest, extending posterior to trigeminal foramen. Gauthier *et al*.^4^. (ordered).

289. Ectopterygoid overlap of pterygoid: (0) short; (1) long. Gauthier *et al*.^4^.

290. Epipterygoid: (0) present; (1) absent. Estes *et al.*^20^.

291. Epipterygoid, in resting position: (0) located lateral to prootic (even if only narrowly so); (1) located entirely anterior to prootic. Gauthier *et al*.^4^.

292. Epipterygoid relative to alar process of prootic: (0) epipterygoid anterolateral to prootic alar process; (1) epipterygoid abuts anteroventral tip of alar process. Gauthier *et al*.^4^.

293. Epipterygoid shortens: (0) long (reaches nearly to level of top of braincase, or above quadrate head, or more than half distance between pterygoid and parietal table); (1) short (reaches only to level of quadrate head, barely to semicircular canal, or half or less of distance

between pterygoid and parietal table). Gauthier *et al*.^4^.

294. Epipterygoid-parietal contact: (0) absent; (1) overlaps parietal temporal muscle origin. Gauthier *et al*.^4^.

295. Epiptergyoid: (0) expanded dorsoventrally and ventrally; (1) columelliform. Estes *et al.*^20^.

296. Braincase fusion: (0) unfused in adult; (1) opisthotic and prootic fused in adult; (2) complete braincase fusion in adult. Gauthier^40^.

297. Processus ascendens of synotic tectum: (0) absent; (1) present. Estes *et al.*^20^.

298. Supraoccipital: (0) single; (1) double. Estes *et al.*^20^.

299. Supraoccipital origin of temporal muscles: (0) restricted to parietal; (1) spread onto supraoccipital contacting nuchal crest in roughly T-shaped outline; (2) spread onto

supraoccipital to form Y-shaped crest; (3) temporal muscles spread onto braincase dorsally, but sagittal and nuchal crests join to form roughly anchor-shaped outline. Gauthier *et al*.^4^..

300. Supraoccipital nuchal crest lateral extent: (0) absent; (1) present on supraoccipital; (2) present on supraoccipital and otoccipital. Gauthier *et al*.^4^. (ordered).

301. Supraoccipital crest: (0) absent; (1) present, Estes *et al.*^20^.; (2) meets

ventral parietal. Rieppel^52^ (ordered).

302. Supraoccipital relative to oto-occipital on midline: (0) overlaps oto-occipital laterally; (1) laps over oto-occipital on midline as part of sagittal crest, Lee and Scanlon^31^; (2) that is, in turn, capped by the parietal, so that all three bones are visible in cross section. Kluge^36^. (ordered).

303. Supraoccipital contribution to internal sidewall of neurocranium: (0) participates in sidewall; (1) absent, only dorsal plate remains; (2) dorsal plate absent. Gauthier *et al*.^4^. (ordered).

304. Epiotic foramen: (0) absent; (1) present. Moody^12^.

305. Prootic, alar process: (0) small or absent; (1) prominent. Gauthier^13^.

306. Prootic, supratrigeminal process: (0) absent; (1) weakly developed, not projecting beyond cupola anterior, Moody^12^; (2) present as a finger-like projection above trigeminal notch, projecting beyond cupola anterior. Estes *et al.*^20^ (ordered).

307. Crista prootica (ridge on lateral surface of the prootic, overhanging facial foramen): (0) well-developed lateral flange; (1) reduced to weak ridge; (2) absent. Presch^53^.

308. Crista prootica: (0) does not extend onto basipterygoid process; (1) extends onto basipterygoid process forming open or closed bony canal. Estes *et al.*^20^

309. Crista prootica aliform in outline in ventral view (extended butterfly shape): (0) absent; (1) present; (2) prominent, extending further laterally. Estes *et al.*^20^ (ordered).

310. Crista tuberalis and crista prootica: (0) separate; (1) combined to surround stapedial footplate and lateral aperture of recessus scalae tympani. Estes *et al.*^20^

311. Crista interfenestralis: (0) prominent; (1) reduced, Rieppel^52^; (2) absent. Rieppel (1984a) (ordered).

312. Crista tuberalis: (0) prominent; (1) reduced; (2) absent. Rieppel^54^. (ordered).

313. Facial foramen (lateral exit on prootic for the facial or VII cranial nerve): (0) single; (1) double. Lee^14^.

314. Prootic participates in medial aperture of the recessus scala tympani (MARST): (0) absent; (1) prootic forms part of MARST. Gauthier and Bell (unpubl. Data in Gauthier *et al*.^4^).

315. Posterior auditory foramen: (0) bordered by opisthotic (=oto-occipital) posteromedially; (1) enclosed entirely in prootic. Gauthier *et al*.^4^.

316. Orbitosphenoid, calcified/ossified: (0) absent; (1) present; (2) expanded to floor the braincase. Wu *et al*.^27^. (ordered).

317. Orbitosphenoid: (0) well developed; (1) reduced. Gauthier *et al*.^4^.

318. Orbitosphenoid: (0) paired; (1) single (fused ventrally). Gauthier *et al*.^4^.

319. Optic foramen: (0) present; (1) absent. Rieppel^55^.

320. Optic foramen: (0) not fully enclosed by bone; (1) enclosed partly or entirely by frontals; (2) entirely within orbitosphenoid; (3) entirely within parietal. Wu *et al.*^27^.

321. Trigeminal foramen or foramina: (0) anterior margin not enclosed in bone; (1) anterior margin enclosed by descending flange of parietal; (2) anterior margin enclosed by orbitosphenoid; (3) enclosed by prootic. Wu *et al.*^27^.

322. Trigeminal nerve maxillary branch: (0) pierces the lateral (maxillary) process of the palatine; (1) passes dorsally between the palatine and the prefrontal. Tchernov *et al*.^33^.

323. Ophidiosphenoid (equals “laterosphenoid” or “pleurosphenoid”): (0) absent; (1) present. Rieppel^56^.

324. Dorsum sellae shape in longitudinal cross-section: (0) crista sellaris forms posterior wall, usually low and vertically disposed with more or less anterior slope; (1) dorsum sellae poorly differentiated with, at most, shallow fossa with low crista sellaris; (2) enclosed in distinct

fossa, a cup-like depression walled laterally and ventrally by the basisphenoid and anteriorly by the parasphenoid rostrum; (3) completely enclosed tube-like dorsum sellae, C.J. Bell (pers. comm. in Gauthier *et al*.^4^.) Rieppel^57^.

325. Dorsum sella fossa roofed posteriorly by crista sellaris (not scored in species with reduced/absent crista sellaris): (0) fossa only modestly roofed by crista sellaris; (1)

roofing more extensive over deep fossa. Gauthier *et al*.^4^.

326. Parabasisphenoidal keel: (0) absent; (1) present below dorsum sellae; (2) deep keel. Kluge^36^. (ordered).

327. Parasphenoid rostrum in cross-section below posterior frontal articulation: (0) somewhat subrectangular; (1) distinctly I-beam shaped, strongly compressed laterally, abruptly narrows at trabeculae; (2) with an arrowhead-shaped apex in cross-section; (3) with prominent ventrolaterally directed alae. Tchernov *et al*.^33^.

328. Cultriform process: (0) long; (1) short; (2) absent. Gauthier *et al*.^4^. (ordered).

329. Vidian canal rostral opening: (0) roofed by parietal; (1) exits via parasphenoid rostrum only. Gauthier *et al*.^4^.

330. Vidian canal opening on right side: (0) is not larger than that of left Vidian canal; (1) is larger than that of the left Vidian canal. Kluge^18^.

331. Trabeculae cranii: (0) tropibasic; (1) platybasic. Rieppel*^54^*.

332. Basipterygoid process (and synovial palatobasal articulation): (0) present, formed by ossified basitrabecular process; (1) present, formed by outgrowth from parabasisphenoid

(no basitrabecular process known; synovial palatobasal articulation absent); (2) basipterygoid

process absent. Rieppel^46^.

333. Basipterygoid process: (0) long, i.e., projecting far beyond the body of the basisphenoid; (1) short, i.e., not projecting very far beyond the body of the basisphenoid. Lee^14^.

334. Basipterygoid process: (0) not expanded at distal end; (1) distal end expanded. Lee^14^.

335. Sesamoid bone at basipterygoid-pterygoid articulation: (0) absent; (1) present. Gauthier *et al*.^4^.

336. Vidian canal formed by the basisphenoid enclosing the internal carotid artery, and the base of the palatine artery, as they pass over the basipterygoid process: (0) absent; (1) present.

Estes *et al.*^20^.

337. Vidian canal caudal opening: (0) within basisphenoid; (1) anterior margin at basisphenoid-prootic suture; (2) entirely within prootic; (3) the dibamid-amphisbaenian condition. Estes *et al.*^20^. (ordered).

338. Carotid artery exits rostral end of Vidian canal: (0) at same level (or slightly above) as the remnant of the embryonic neurocranial trabeculae; (1) below the level of the remnant of the embryonic neurocranial trabeculae. Conrad and Norell^58^.

339. Basal tubera position: (0) posterolateral, with apex on lateral edge of basioccipital just behind base of prooticopisthotic suture; (1) anteromedial, with apex at lateral juncture

of sphenoid and basioccipital, anterior and medial to prootic-opisthotic suture. Gauthier *et al*.^4^.

340. Apophyseal ossification (Element “X”) caps basal tubera: (0) absent; (1) present; (2) huge. Gauthier *et al*.^4^. (ordered).

341. Occipital condyle: (0) posterior surface of condyle straight in ventral view; (1) posterior surface of condyle concave in ventral view. Lee^14^.

342. Basioccipital: (0) contributes to ventral border of foramen magnum; (1) excluded from ventral border of foramen magnum by contact of exoccipitals. Rieppel^59^.

343. Basioccipital ventral keel: (0) absent; (1) crest; (2) keel. Kluge^36^. (ordered).

344. Medial aperture of the recessus scala tympani (MARST): (0) between basioccipital and opisthotic; (1) entirely in opisthotic. Gauthier *et al*.^4^.

345. Cranial nerve IX exits braincase via: (0) MARST internally and lateral aperture of recessus scala tympani (LARST) externally; (1) exits dorsal to MARST then out

LARST. Gauthier *et al*.^4^.

346. Cranial nerve IX exits braincase via: (0) foramen magnum; (1) laterally via LARST; (2) posteriorly via vagus (=jugular) foramen. Rieppel^60^. (ordered).

347. Medial aperture of the recessus scalae tympani (MARST) subdivided, IX cranial nerve exits posteriorly: (0) absent; (1) large oval MARST undivided, with IX cranial

nerve exiting at posterodorsal end; (2) MARST divided into anterior and posterior openings by bony process, with IX cranial nerve exiting via posterodorsal foramen. Gauthier and Bell (in Gauthier *et al*.^4^) (ordered).

348. Vagus foramen (“jugular foramen” in other amniotes) far from MARSTN: (0) with hypoglossal foramina lying below and between them medially; (1) vagus foramen

closer to MARST, with hypoglossal foramina extending posterior to vagus. Gauthier *et al*.^4^.

349. Hypoglossal (XII) foramina exit(s) relative to vagus (X–XI) foramen on external surface of braincase: (0) hypoglossal foramina separated from vagus (jugular) foramen (Conrad 2008); (1) at least one hypoglossal foramen emerges from the same fossa as the vagus foramen; (2) only one hypoglossal foramen still exits separately from the vagus foramen fossa; (3) all three hypoglossals emerge from the same fossa as the vagus foramen. Lee^49^. (ordered).

350. LARST (lateral aperture of recessus scalae tympani): (0) open; (1) small, Rieppel^52^; (2) closed. Rieppel^55^. (ordered).

351. Perilymphatic foramen faces: (0) ventrally; (1) medially; (2) laterally; (3) posteriorly. Rieppel^57^.

352. Opisthotic-exoccipital fusion to form oto-occipital: (0) incompletely fused or separate in adult; (1) completely fused early in post-hatching ontogeny. Estes *et al.*^20^.

353. Oto-occipitals (exoccipital part) contact above foramen magnum to exclude supraoccipital: (0) absent; (1) present. Estes *et al.*^20^.

354. Metotic fissure: (0) open; (1) subdivided by contact of basal plate and otic capsule. Gauthier, Estes and de Queiroz^10^.

355. Mandibular symphysis: (0) anterior tips of dentary with distinct flat symphyseal area; (1) anterior tips of dentary smoothly rounded and without distinct symphyseal area. Lee^14^.

356. Dentary anterodorsal edge of dental parapet at tip: (0) straight; (1) tipped down (and medially). Gauthier *et al*.^4^.

357. Dentary bowed ventrally along long axis: (0) straight to slightly bowed; (1) distinctly bowed ventrally. Conrad^61^.

358. Dentary overlaps postdentary bones laterally: (0) extensive; (1) reduced. Gauthier^13^.

359. Dentary suspended from: (0) overlapping parts of coronoid, surangular, prearticular, splenial and angular; (1) surangular; (2) prearticular. Gauthier^13^.

360. Dentary subdental shelf/gutter development in anterior part of dentary: (0) subdental shelf absent; (1) weakly developed subdental shelf; (2) pronounced subdental gutter. Estes *et al.*^20^. (ordered).

361. Dentary, number of mental foramina on lateral surface: (0) none; (1) one; (2) two; (3) three; (4) four or more. Lee^14^. (ordered).

362. Dentary, size of posteriormost mental foramen: (0) same size as others; (1) enlarged relative to others. Gauthier *et al*.^4^.

363. Dentary mental foramen position: (0) near tip of dentary; (1) displaced caudally; (2) displaced further caudally. Gauthier *et al*.^4^.

364. Dentary coronoid process posterior termination: (0) below (or anterior) to level of coronoid apex; (1) just behind level of coronoid apex; (2) well posterior to level of coronoid

apex. Gauthier *et al*.^4^.

365. Dentary subdental shelf hooks around anterior rim of the anterior inferior alveolar foramen: (0) absent; (1) present. Gauthier^13^.

366. Dentary surangular process: (0) lies flat against the dorsolateral face of the surangular below the coronoid; (1) set in a posterodorsally trending groove, open dorsally, that supports it from below on the dorsolateral face of the surangular below the coronoid; (2) set in deep V-shaped, laterally-facing recess on dorsolateral face of surangular behind coronoid. Gauthier *et al*.^4^.

367. Dentary coronoid process posterodorsal extension: (0) absent or with only small dorsal extension; (1) large, but extending between lateral and medial processes of coronoid; (2) large, but extending dorsally to overlap most of anterolateral surface of coronoid; (3) extremely well developed, covering almost entire lateral surface of coronoid. Estes *et al.*^20^. (ordered).

368. Dentary angular process reduced; (0) angular process extends to or past coronoid apex; (1) anterior to coronoid apex; (2) anterior to level of coronoid bone. Gauthier^13^. (ordered).

369. Dentary posterior termination on latral face of mandible: (0) below (or anterior to) level of coronoid apex; (1) just posterior to coronoid apex; (2) well posterior to level of coronoid apex; (3) nearly to posterior surangular foramen. (ordered). Etheridge and de Queiroz^30^.

370. Dentary angular process prominently bifid: (0) absent; (1) present. Gauthier *et al*.^4^.

371. Meckel’s canal: (0) opens medially for most of length; (1) opens ventrally anterior to anterior inferior alveolar foramen. Gauthier^13^.

372. Dentary restricts Meckel’s canal: (0) does not restrict or enclose Meckelian canal; (1) lower dentary border of Meckel’s canal folds up to approach closely upper border to restrict canal; (2) upper and lower borders form sutural contact anterior to splenial; (3) Meckel’s canal closed and fused anterior to splenial. Etheridge and de Queiroz^30^. (ordered).

373. Splenial attachment to dentary above Meckel’s canal: (0) close throughout length; (1) loose, with dorsal dentary suture confined to posterodorsal corner of splenial. Pregill *et al*.^19^.

374. Splenial: (0) present; (1) absent; (2) fused to dentary. Gauthier, Estes and de Queiroz^10^.

375. Splenial anterior extent: (0) around one-third (or less) length relative to dentary tooth row; (1) about one-half; (2) about two-thirds; (3) three-fourths (or more). Estes *et al.*^20^. (ordered).

376. Splenial posterior extent: (0) extends posteriorly to or beyond apex of coronoid; (1) does not extend posteriorly to apex of coronoid. Pregill *et al*.^19^.

377. Splenial-angular articulation: (0) splenial overlaps angular; (1) with ball on splenial (below level of posterior mylohyoid foramen) fitting into socket on angular; (2) with ball on angular fitting into socket on splenial; (3) flat, abutting joint. Gauthier *et al*.^4^.

378. Splenial anterior inferior alveolar foramen (aiaf) position relative to dentary: (0) enclosed entirely in splenial; (1) between splenial and dentary. Gauthier *et al*.^4^.

379. Splenial anterior inferior alveolar foramen position relative to anterior mylohyoid foramen: (0) anterodorsal; (1) dorsal to posterodorsal. Gauthier *et al*.^4^.

380. Angular: (0) present; (1) absent. Estes *et al.*^20^.

381. Angular posterior extent: (0) reaches mandibular condyle; (1) does not reach mandibular condyle. Gauthier, Estes and de Queiroz^10^.

382. Angular taller anteriorly, closely approaching coronoid (or, if coronoid absent, tooth-bearing margin of dentary above Meckelian canal): (0) absent, angular broadly separated

from coronoid; (1) present; (2) with finger-like process over-arching Meckel’s canal. Gauthier *et al*.^4^.

383. Angular medial exposure (relative degree of medial exposure scored with the teeth pointing straight up): (0) broad; (1) reduced; (2) narrow. Lee^14^.

384. Posterior mylohyoid foramen position: (0) absent; (1) medial; (2) ventral; (3) lateral. Gauthier *et al*.^4^.

385. Posterior mylohyoid foramen position relative to coronoid apex: (0) below; (1) posterior; (2) anterior. Frost & Etheridge^43^.

386. Coronoid eminence: (0) present; (1) absent. Gauthier, Estes and de Queiroz^10^.

387. Coronoid eminence composition: (0) formed by both surangular and coronoid; (1) formed exclusively by coronoid; (2) formed exclusively by surangularN. Gauthier, Estes and de Queiroz^10^.

388. Coronoid anteromedial process fits into sulcus beneath tooth-bearing border of dentary (at or behind end of tooth row): (0) absent; (1) present; (2) and wraps around ventral margin of dentary tooth-bearing border at apex posteriorly. Smith^24^ (ordered).

389. Coronoid bone: (0) present, well develope; (1) present, small and straplike; (2) absent. Gauthier *et al*.^4^. (ordered).

390. Coronoid-surangular articulation: (0) coronoid restricted to medial aspect of mandible; (1) coronoid extends onto dorsal surface of surangular; (2) coronoid arches over dorsal margin of mandible to reach lateral face of surangular. Estes *et al.*^20^. (ordered).

391. Coronoid, anteromedial process: (0) present; (1) absent. Lee^14^.

392. Coronoid, anteromedial ventral margin (at/behind end of tooth row): (0) overlapped by splenial; (1) abuts splenial; (2) does not contact splenial. Lee^14^. (ordered).

393. Coronoid, posteromedial process: (0) absent; (1) present. Lee^14^.

394. Coronoid, anterolateral dentary process: (0) absent; (1) present; (2) overlaps dentary past level of tooth row. Gauthier *et al*.^4^. (ordered).

395. Coronoid, shape of anterolateral dentary process: (0) extends anteroventrally and smoothly tapers into dentary; (1) extends anteriorly, with dorsal and ventral margins more

parallel sided, terminating in a blunt edge anteriorly. Gauthier *et al*.^4^.

396. Surangular inserts into dentary lateral to the intramandibular septum, entering the intramandibular canal (which houses the alveolar branch of the inferior alveolar nerve, according to Oelrich 1956): (0) absent; (1) present slightly; (2) present deeply. Gauthier^13^. (ordered).

397. Surangular, external foramina: (0) two foramina, anterior and posterior; (1) single foramen (anterior surangular foramen). Gauthier *et al*.^4^.

398. Adductor fossa: (0) faces dorsomedially, medial wall below lateral wall; (1) faces dorsally, medial/lateral walls same height; (2) no distinct medial wall; (3) faces dorsolaterally, lateral wall below medial wall. Lee^14^.

399. Surangular adductor fossa on external face of mandible: (0) shallow and extends ventrally no more than halfway down; (1) deep and extends ventrally more than half way down (nearly to angular bone). Gauthier^40^.

400. Surangular dorsal margin: (0) nearly horizontal, rising somewhat toward the coronoid, anterodorsal edge set below level of tooth crowns; (1) rises steeply anterodorsally to coronoid,

with apex reaching above level of tooth crowns. Gauthier *et al*.^4^.

401. Prearticular and surangular fused in adult: (0) separate; (1) fused. Gauthier *et al*.^4^.

402. Prearticular broadly contacts surangular behind posteromedial process of coronoid, restricting mandibular adductor fossa anteriorly. (0) absent; (1) present. Gauthier *et al*.^4^.

403. Prearticular crest: (0) absent; (1) present. Estes *et al.*^20^.

404. Retroarticular process: (0) present; (1) very short or absent. Gauthier, Estes and de Queiroz^10^.

405. Retroarticular process orientation: (0) not inflected medially; (1) inflected medially. Estes *et al.*^20^.

406. Retroarticular process orientation in lateral (or posterior) view: (0) extends straight posteriorly; (1) inflected ventrally. Gauthier^13^.

407. Retroarticular process dorsal surface: (0) horizontal; (1) inclined posterodorsally. Gauthier *et al*.^4^.

408. Retroarticular process emarginate distally: (0) absent; (1) present. Gao and

Norell^62^.

409. Retroarticular process, lateral notch forming waist proximally: (0) absent; (1) present. Estes et al. (1988).

410. Retroarticular process breadth (greatest width) relative to mandibular condyle (glenoid): (0) narrower; (1) wider. Estes *et al.*^20^.

411. Prearticular, pterygoideus process (angular process of Oelrich^63^) (i.e., part of retroarticular process): (0) absent; (1) present. Estes *et al.*^20^.

412. Premaxillary teeth (apart from median tooth): (0) similar size or larger than anterior maxillary teeth; (1) distinctly smaller than anterior maxillary teeth. Pregill et al. (1986).

413. Median premaxillary tooth: (0) absent; (1) present. Lee^14^.

414. Enlarged median tooth on fused premaxilla: (0) median tooth same size as other premaxillary teeth; (1) slightly enlarged median premaxillary tooth; (2) greatly enlarged median premaxillary tooth. Lee^14^. (ordered).

415. Maxillary tooth row extent posteriorly: (0) to roughly midorbit (or anterior); (1) to posterior third of orbit; (2) posterior to orbit. Gauthier *et al*.^4^. (ordered).

416. Maxillary tooth crown height: (0) constant throughout tooth row; (1) length varies, resulting in sinuous occlusal surface; (2) length varies, resulting in convex occlusal surface; (3) length decreases posteriorly; (4) length increases posteriorly. Gauthier *et al*.^4^.

417. Maxilla, enlarged teeth (“fangs”) (relative to adjacent teeth): (0) absent; (1) present on anterior maxilla; (2) present on posterior maxilla. Gauthier *et al*.^4^.

418. Maxilla tooth row length: (0) to or behind midorbit; (1) anterior to midorbit; (2) anterior to orbit. Pregill *et al.*^19^ (ordered).

419. Premaxillary tooth count: (0) none; (1) one to three; (2) four to six; (3) seven to nine; (4) 10 or more. de Queiroz^44^. (ordered).

420. Maxillary tooth count: (0) 0, Conrad^61^; (1) 2–5; (2) 7–15; (3) 16–27; (4) 31 or more. Lee^14^. (ordered).

421. Dentary tooth count: (0) 0, Conrad^61^; (1) 4–9; (2) 10–20; (3) 21–35; (4) 36 or more. Lee^14^. (ordered).

422. Marginal teeth: (0) all vertical; (1) all recurved; (2) anterior teeth recurved and posterior teeth vertical. Gauthier *et al*.^4^.

423. Position of marginal teeth relative to tooth-bearing element: (0) on medial side of tooth-bearing element; (1) near/on apical margin of tooth-bearing element. Estes *et al.*^20^.

424. Fusion of marginal teeth: (0) unfused to each other; (1) fused to each other. Gauthier *et al*.^4^.

425. Bases of marginal teeth: (0) smooth, dentine and enamel not infolded; (1) dentine and enamel infolded into pulp cavity (“plicidentine’), resulting in longitudinal grooves externally at base of teeth. Pregill *et al.*^19^.

426. Bases of marginal teeth expanded: (0) absent; (1) present. Conrad^61^.

427. Marginal tooth spacing: (0) crowns closely spaced; (1) crowns separated by large gaps. Lee^14^.

428. Position of replacement teeth: (0) lingual; (1) posterolingual. Pregill *et al*.^19^.

429. Orientation of replacement teeth: (0) erupt upright, growing straight upwards into functional position; (1) erupt horizontally, and then rotate through 90° about the base into

functional position. Lee^64^.

430. Tooth replacement: (0) present; (1) absent. Gauthier *et al*.^4^.

431. Resorption pits: (0) present; (1) absent. Pregill *et al.*^19^.

432. Development of resorption pits: (0) at base of teeth; (1) on bony tooth pedicel. Rieppel and Zaher^65^.

433. Palatal teeth: (0) constant in size across palatal tooth row; (1) decrease in size posteriorly. Mahler and Kearney^51^.

434. Cusps on posterior teeth: (0) unicuspid; (1) bicuspid; (2) tricuspid. Gauthier *et al*.^4^.

435. Venom groove on anteromedial surface of teeth: (0) absent; (1) present, unenclosed; (2) present, enclosed tube. Gauthier *et al*.^4^. (ordered).

436. V-shaped wear facets of maxillary teeth incised on lateral face of dentary between dentary teeth: (0) absent; (1) present. Evans *et al.*^66^.

437. Teeth swollen, set off from tooth shafts above jaw parapet: (0) absent; (1) present. Gauthier *et al*.^4^.

438. Basihyal: (0) present; (1) absent. Gauthier *et al*.^4^.

439. Basihyal, relationship to skull (when mouth is closed): (0) anterior to braincase; (1) ventral to braincase; (2) posterior to braincase. Gauthier *et al*.^4^.

440. Hyoid, lingual process length: (0) short; (1) medium; (2) long. Gauthier *et al*.^4^. (ordered).

441. Hyoid, distal part of lingual process: (0) not detached; (1) detached. Schwenk^67^.

442. Free epibranchials (second epibranchial): (0) absent; (1) present. Gauthier, Estes and

de Queiroz^10^.

443. Free epibranchial: (0) simple (short or sigmoidal); (1) complex (has hooks

or processes, and/or doubles back on itself). Gauthier *et al*.^4^.

444. First epibranchial: (0) shorter than first ceratobranchial; (1) longer than or nearly equal to first ceratobranchial. Gauthier *et al*.^4^.

445. First ceratobranchial (in lateral view): (0) no dorsolateral angulation; (1) weak dorsolateral angulation (has a distinct bend); (2) strong dorsolateral angulation (90° or more); (3) entire element straight or oriented vertically Gauthier *et al*.^4^.

446. Second ceratobranchials: (0) present; (1) absent. Estes *et al.*^20^.

447. Second ceratobranchial: (0) shorter than first ceratobranchial, Lang (1989); (1) nearly equal to or longer than first ceratobranchial. Etheridge and de Queiroz^30^.

448. Second ceratobranchial apposed on midline: (0) absent; (1) present. McGuire^68^.

449. Large, wing-like hyoid cornu: (0) absent; (1) present. Kluge^69^.

450. Hyoid cornu: (0) less than the length of the epihyal; (1) greater than or equal to the length of the epihyal. Presch^53^.

451. Epihyal: (0) meets hyoid cornu at (or near) its distal end; (1) meets hyoid cornu along its body. Gauthier *et al*.^4^.

452. Epihyal: (0) expansion or elaboration at proximal end absent; (1) simple expansion at proximal end present; (2) hook-like elaboration at proximal end present; (3) lateral flange at proximal end present; (4) medial flange at proximal end present. Gauthier *et al*.^4^.

453. Lateral flange at midpoint of epihyal: (0) absent; (1) present. Gauthier *et al*.^4^.

454. Presacral vertebrae number reduction: (0) 24 or more presacrals; (1) 23 presacrals, Etheridge and de Queiroz30; (2) fewer than 23 presacrals. Estes *et al.*^20^. (ordered).

455. Presacral vertebrae number increase I: (0) 24 or fewer; (1) 25; (2) 26; (3) 27; (4) 28 or more. Estes *et al.*^20^. (ordered).

456. Presacral vertebrae number increase II: (0) 32 presacrals or fewer; (1) 33–39; (2) 50–55; (3) 61–84; (4) 89 or more. Lee and Scanlon^31^. (ordered).

457. Presacral vertebrae number increase III: (0) less than 104; (1) 118–132 (2) 144–156; (3) 168–180; (4) 184 or more. Lee and Scanlon^31^. (ordered).

458. Presacral vertebrae number increase IV: (0) less than 193; (1) 197–209; (2) more than 219. Lee and Scanlon^31^. (ordered).

459. Cervical vertebra number reduction: (0) six or more; (1)N five; (2) four; (3) three; (4) two. Estes *et al.*^20^. (ordered).

460. Cervical vertebrae number increase: (0) six or fewer; (1) seven; (2) eight or more. Pregill *et al*.^19^. (ordered).

461. Cervical intercentrum position: (0) intercentral; (1) posterior end of preceding centrum; (2) anterior end of following centrum; (3) absent. Estes *et al.*^20^.

462. Cervical rib ossified portion shape: (0) widens distally, at least in last cervical; (1) tapers distally. Gauthier *et al*.^4^.

463. Cervical ribs start on vertebra number: (0) two; (1) three; (2) four; (3) five; (4) six. Estes *et al.*^20^. (ordered).

464. Cervical intercentrum length relative to pedicle length: (0) intercentrum longer than pedicle; (1) intercentrum shorter than pedicle. Gauthier *et al*.^4^.

465. Cervical pedicle (outgrowth of pleurocentrum to which intercentrum may attach): (0) absent; (1) projecting ventrally with discrete fore and aft margins. Pregill *et al*.^19^.

466. Vertebral pedicle (“hypapophysis”) posterior extent: (0) in anterior half of vertebral column; (1) throughout vertebral column.

467. Vertebral centrum articulation: (0) amphicoelous (and notochordal); (1) procoelous. Gauthier, Estes and de Queiroz^10^.

468. Zygosphene-zygantrum accessory intervertebral articulations: (0) absent; (1) dorsolaterally directed facet continuous with prezygapophyseal articulation located just up edge of neural arch, Gauthier, Estes and de Queiroz^10^; (2) tall, laterally directed facet continuous with prezygapophyseal articulation and extending as high as top as neural canal; (3) separate facet set on distinct pedicle and facing ventrolaterally, de Queiroz^44^. Estes *et al.*^20^. (ordered).

469. Vertebrae (and ribs), mid-dorsals are pachyostotic: (0) absent; (1) present. Rieppel and Head^70^.

470. Caudal autotomic septum position relative to caudal rib: (0) within caudal rib; (1) anterior to caudal rib; (2) posterior to caudal rib; (3) absent. Estes *et al.*^20^.

471. Caudal rib (transverse process) shape: (0) single rib without basal foramen; (1) foramen passing through base of rib; (2) divergent bifid ribs on some caudals. Estes *et al.*^20^.

472. Posterior caudal vertebrae, groove on dorsal surface of neural spines: (0) absent; (1) present. Kluge^18^.

473. Caudal vertebrae, pterapophysis: (0) absent; (1) present. Kluge^18^.

474. Caudal vertebrae, distal tip of anterior zygapophyses: (0) undifferentiated; (1) elaborated into a horizontal blade. Kluge^36^.

475. Caudal haemal arch (intercentrum) position: (0) intercentral, pedicles feeble/absent; (1) contacting mainly condyle but also distinct pedicles beneath preceding centrum; (2) mainly contacting pedicles on preceding centrum but still bordering condyle; (3) well forward of

condyle on preceding centrum. Pregill *et al.*^19^. (ordered).

476. Caudal haemal arch pedicle length: (0) short; (1) long. Pregill *et al.*^19^.

477. Vertebra whose rib first attaches to sternum: (0) seventh (or more anteriorly); (1) eighth; (2) ninth. Estes *et al.*^20^. (ordered).

478. Trunk ribs pachyostotic: (0) slender, cancellous ribs; (1) thick, dense ribs. Caldwell^48^.

479. Postcloacal bones: (0) absent; (1) present. Estes *et al.*^20^.

480. Sternum: (0) present; (1) absent. Lee^14^.

481. Sternal fontanelle: (0) absent; (1) present. Estes *et al.*^20^.

482. Sternal fontanelle number: (0) single; (1) double. Moody^12^.

483. Number of rib attachment points to sternum (including attachment of xiphisternum): (0) five; (1) four; (2) three; (3) two or fewer. Gauthier, Estes and de Queiroz^10^ (ordered).

484. Xiphisternum: (0) present; (1) absent. Gauthier, Estes and de Queiroz^10^.

485. Xiphisternal fontanelle: (0) absent; (1) present.

486. Number of xiphisternal rib attachment points: (0) none; (1) one; (2) two; (3) three; (4) four. Gauthier *et al*.^4^. (ordered).

487. Number of postxiphisternal (or poststernal) inscriptional ribs united along the ventral midline to form continuous chevron shaped structures: (0) 0; (1) 1–4; (2) 5–11; (3) 12–30; (4) more than 31. Estes *et al.*^20^. (ordered).

488. Scapulocoracoid: (0) large; (1) reduced; (2) absent. Lee^14^. (ordered).

489. Scapula: (0) short and wide; (1) elongate and thin. Grismer^71^.

490. Suprascapular cartilage: (0) present; (1) absent. Gauthier *et al*.^4^.

491. Suprascapula: (0) large (approximately equal to length of scapula); (1) small. Estes *et al.*^20^.

492. Scapula, emargination on anterodorsal edge (scapular fenestra): (0) absent; (1) present. Estes *et al.*^20^.

493. Scapulocoracoid emargination: (0) absent; (1) present. Gauthier, Estes and de Queiroz^10^.

494. Scapulocoracoid emargination: (0) closed by cartilage; (1) open; (2) closed by scapula and coracoid. Gauthier *et al*.^4^.

495. Coracoid, anterior (primary) emargination (fenestra): (0) absent; (1) present. Pregill *et al*.^19^.

496. Coracoid, posterior (secondary) emargination (fenestra): (0) absent; (1) present. Pregill *et al*.^19^.

497. Coracoid size: (0) enlarged, extending anteriorly to level of clavicles; (1) not enlarged, not extending anteriorly to level of clavicles. Gauthier *et al*.^4^.

498. Epicoracoid cartilage extent: (0) contacts mesoscapula and suprascapula; (1) does not contact mesoscapula and suprascapula. Pregill *et al.*^19^.

499. Clavicle: (0) present; (1) absent. Estes *et al.*^20^.

500. Clavicle: (0) no notch or fenestration present; (1) notch present; (2) fenestration present. Etheridge and de Queiroz^30^.

501. Clavicle: (0) rod-like; (1) greatly expanded proximally. Gauthier^13^.

502. Clavicular angulation: (0) simple curved rod, following contour of scapulocoracoid; (1) strongly angulated, curving anteriorly away from scapulocoracoid. Estes *et al.*^20^.

503. Distal clavicle articulation: (0) with scapula; (1) with suprascapula; (2) no distal articulation. Gauthier, Estes and de Queiroz^10^.

504. Clavicles, medial contact: (0) clavicles do not meet on midline; (1) clavicles meet on midline. Gauthier *et al*.^4^.

505. Interclavicle: (0) present; (1) absent. Estes *et al.*^20^.

506. Interclavicle lateral process: (0) present; (1) absent. Estes *et al.*^20^.

507. Interclavicle anterior process (extending beyond lateral process): (0) absent; (1) present. Gauthier^13^.

508. Interclavicle anterior process, length (as ratio of interclavicle length): (0) 0.01-0.20; (1) more than 0.20. Gauthier^13^.

509. interclavicle, anterior end: (0) ventral to clavicles; (1) dorsal to clavicles; (2) abuts clavicles; (3) lies posterior to clavicles. Gauthier *et al*.^4^.

510. Pubis: (0) present; (1) absent. Lee^14^.

511. Pubis, symphyseal process orientation in ventral view: (0) medially directed; (1) anteromedially directed. Estes *et al.*^20^.

512. Pubis, symphyseal process: (0) thick; (1) thin. Estes *et al.*^20^.

513. Pubis, symphyseal process: (0) expanded distally; (1) tapered, not expanded distally. DeBraga and Carroll^47^.

514. Pectineal (pubic) tubercle: (0) closer to acetabulum than to symphysis; (1) closer to symphysis than to acetabulum (or equal distance). Estes *et al.*^20^.

515. Pubis, tubercle orientation in ventral view: (0) anteriorly directed; (1) ventrally directed. Gauthier *et al*.^4^.

516. Ischium: (0) present; (1) absent. Lee^14^.

517. Ischial tubercle: (0) present; (1) absent, or continuous with hypoischial cartilage. Estes *et al.*^20^.

518. Hypoischium: (0) well developed (expanded at distal end); (1) vestigial (no expansion at distal end); (2) absent. Lee^72^. (ordered).

519. Hypoischial foramen: (0) absent; (1) present. Gauthier *et al*.^4^.

520. Ilium: (0) present; (1) absent. Lee^14^.

521. Ilium, tubercle: (0) present; (1) absent. Lee^14^.

522. Ilium, blade orientation: (0) slopes posterodorsally; (1) oriented vertically; (2) oriented anteriorly. Bell^21^.

523. Ilium, dorsal ends blades: (0) not compressed laterally, and do not expand or converge dorsomedially; (1) compressed laterally, suprailiac cartilages expanded into triangular

plates that converge dorsomedially. Estes *et al.*^20^.

524. Pelvic elements (ilium, ischium, pubis): (0) in close sutural contact throughout postnatal ontogeny and co-ossified into a single pelvic bone late in postnatal ontogeny; (1) distinct elements weakly united in non-sutural contacts (Lee^14^). Gauthier, Estes and de Queiroz^10^.

525. Hyperischium: (0) present; (1) absent. Gauthier *et al*.^4^.

526. Hyperischial foramen: (0) absent; (1) present. Gauthier *et al*.^4^.

527. Epiphyses on long bones: (0) present; (1) absent. Gauthier, Estes and de Queiroz^10^.

528. Proximal forelimb long bones (humerus, radius and ulna): (0) present; (1) absent. Lee^14^.

529. Ratio of radius/ulna to humerus: (0) 0.50–0.61; (1) 0.62–0.97; (2) 0.98–1.10. Gauthier *et al*.^4^. (ordered).

530. Ectepicondylar foramen: (0) present; (1) absent. Estes *et al.*^20^.

531. Ulnar patella: (0) present; (1) absent. Gauthier *et al*.^4^.

532. Ulna, olecranon process on proximal epiphysis: (0) prominent; (1) short or absent. Gauthier, Estes and de Queiroz^10^.

533. Ulna, enlarged distal epiphysis that is nearly hemispherical in profile and fits into a concomitantly enlarged depression on the ulnare: (0) absent; (1) present. Gauthier, Estes and de Queiroz^10^.

534. Radius, styloid process: (0) absent; (1) present on posterolateral surface of distal epiphysis. Gauthier, Estes and de Queiroz^10^.

535. Carpal intermedium: (0) large; (1) small; (2) absent. Gauthier, Estes and de Queiroz^10^. (ordered).

536. Ball and socket intercarpal joint formed by large central carpal or lateral centrale (ball) and radiale, ulnare and pisiform (socket): (0) absent; (1) present. Estes *et al.*^20^.

537. Lateral centrale in hand: (0) separated from second distal carpal; (1) contacting second distal carpal. Gauthier, Estes and de Queiroz^10^.

538. Proximal end of first metacarpal: (0) separated from medial centrale; (1) contacting medial centrale. Gauthier, Estes and de Queiroz^10^.

539. Palmar sesamoid: (0) absent; (1) present. Gauthier *et al*.^4^.

540. Metacarpals II–IV: (0) longer than proximal phalanges; (1) shorter than proximal phalanges. Estes *et al.*^20^.

541. Metacarpals, sesamoids ventral to distal heads: (0) absent; (1) present. Gauthier *et al*.^4^.

542. Phalangeal count, reduction in manus digits II–IVN: (0) three, four, five; (1) reduced to three in digits III and IV; (2) reduced to four in digit IV; (3) reduced to three in digit III and four in digit IV. Gauthier *et al*.^4^.

543. Phalangeal count, digit V of manus: (0) three; (1) two; (2) four. Gauthier *et al*.^4^.

544. Hyperphalangy in manus: (0) absent; (1) present in more than one digit; (2) present only in digit 1; (3) present only in digit 5. Bell^21^.

545. Opposing digits in manus: (0) digits nonopposing; (1) digits 1, 2 and 3 opposing digits 4 and 5. Estes *et al.*^20^.

546. Penultimate phalanges in hand: (0) shorter than or equal to antepenultimate; (1) longer than antepenultimate. Gauthier *et al*.^4^.

547. Sesamoids dorsal to distal heads of penultimate phalanges (manus): (0) present; (1) absent. Gauthier *et al*.^4^.

548. Femur: (0) present; (1) absent. Lee^14^.

549. Femur: (0) curved in dorsoventral plane; (1) not curved in dorsoventral plane. Lee^14^.

550. Femur, internal trochanter: (0) well developed as a prominent, distinct head; (1) poorly developed or absent. Estes *et al.*^20^.

551. Tibial patella: (0) present; (1) absent. Gauthier *et al*.^4^.

552. Tibial lunula: (0) present; (1) absent. Gauthier *et al*.^4^.

553. Fibular lunula: (0) present; (1) absent. Gauthier *et al*.^4^.

554. Dorsal and ventral tibiofemoral lunulae: (0) both present and separate; (1) ventral present, dorsal absent; (2) both absent; (3) both present and fused; (4) dorsal present,

ventral absent. Gauthier *et al*.^4^.

555. Tibia, notching of distal epiphysis: (0) notch not present, epiphysis gently convex for astragalocalcaneal articulation; (1) distinct notch present, fitting onto a ridge on the astragalocalcaneum. Estes *et al.*^20^.

556. Fibulo-astragalar joint: (0) occupies less than half of distal end of fibula; (1) involves most of distal end of fibula. Gauthier, Estes and de Queiroz^10^.

557. Tibia and fibula: (0) remain widely separated at distal ends; (1) very close or in contact at distal ends. Gauthier *et al*.^4^.

558. Ball and socket intertarsal joint formed by distal tarsal 4 (ball) and astragalocalcaneum (socket): (0) absent; (1) present. Estes *et al.*^20^.

559. Third distal tarsal: (0) present; (1) absent. Gauthier *et al*.^4^.

560. Second distal tarsal: (0) present; (1) absent. Gauthier, Estes and de Queiroz^10^.

561. Astragalus and calcaneum: (0) fused with no suture visible in adult, Gauthier, Estes and de Queiroz^10^; (1) co-ossified with suture visible; (2) separated. Lee^14^.

562. Sesamoid between metatarsal I and astragalocalcaneum (ventrally): (0) present; (1) absent. Gauthier *et al*.^4^.

563. Metatarsals II–IV: (0) longer than proximal phalanges; (1) shorter than proximal

phalanges. Estes *et al.*^20^.

564. Metatarsal V: (0) hooked; (1) broad proximally, but not hooked. Gauthier, Estes and de Queiroz^10^.

565. Metatarsals, sesamoids ventral to distal heads: (0) absent; (1) present. Gauthier *et al*.^4^.

566. Phalangeal counts, reduction in pes: (0) two, three, four, five, four; (1) reduced to three phalanges in digits III, IV and V; (2) reduced to four phalanges in digit IV and

three phalanges in digit V; (3) reduced to two phalanges in digit V; (4) reduced to three phalanges in digit V. Gauthier *et al*.^4^.

567. Hyperphalangy in digits of pes: (0) absent; (1) present in more than one digit; (2) present only in digit V. Bell^21^.

568. Opposing digits in pes: (0) digits non-opposing; (1) digits 1 and 2 opposing

digits 3, 4 and 5. Estes *et al.*^20^.

569. Sesamoids dorsal to distal heads of penultimate phalanges: (0) present; (1)

absent. Gauthier *et al*.^4^.

570. Osteoderms on body (and/or tail): (0) not imbricate; (1) imbricate, with gliding surface anteriorly; (2) imbricate anteroposteriorly (with gliding surface), but interdigitate laterally.

Gauthier^13^. (ordered).

571. Osteoderm ornamentation: (0) vermiculate or smooth; (1) tuberculate. Gauthier^13^.

572. Dermal skull bone ornamentation: (0) smooth; (1) lightly rugose about frontoparietal suture; (2) present over dorsum; (3) present on jugal postorbital bar. Estes *et al.*^20^. (ordered).

573. Palpebral osteoderm below supraorbital scales (and their osteoderms): (0) absent; (1) present. Pregill *et al*.^19^.

574. Supracilliary osteoderm (pierced vertically by foramina): (0) absent; (1) present. Gauthier *et al*.^4^.

575. Osteoderms inside supraorbital scales: (0) absent; (1) single; (2) compound. Estes et al. (1988).

576. Supraorbital osteoderms insert into sulcus along frontal supraorbital margin: (0) absent; (1) present. Gauthier *et al*.^4^.

577. Osteoderms in cheek scales: (0) absent; (1) single; (2) compound. Estes *et al.*^20^.

578. Osteoderms in gular scales: (0) absent; (1) single; (2) compound. Gauthier *et al*.^4^.

579. Osteoderms in dorsal scales: (0) absent; (1) single; (2) compound. Gauthier^13^.

580. Osteoderms in ventral scales: (0) absent; (1) single; (2) compound. Gauthier^13^.

581. Osteoderms in skull roof scales: (0) single; (1) compound. Gauthier^13^.

582. Osteoderms invest imbricate caudal scales: (0) absent; (1) present.

583. Mineralized cranial scales hinges: (0) absent; (1) present. Gauthier *et al*.^4^.

584. Scleral ossicle count: (0) 16 or more; (1) 14–15; (2) 12–13; (3) 11 or fewer. Pregill *et al*.^19^. (ordered).

585. Scleral ossicles: (0) complex and irregular in shape; (1) square or rectangular in shape. Lee^14^.

586. Interorbital septum: (0) present; (1) absent. Hallermann^73^.

587. Statolithic masses: (0) absent; (1) present. Gauthier *et al*.^4^.

588. Calcified endolymph: (0) absent Moody (1980); (1) present, but confined to occiput; (2) present, extends posteriorly into neck. Estes *et al.*^20^. (ordered).

589. Foretongue retracts into hind tongue: (0) absent; (1) present; (2) tongue can be retracted entirely into buccal cavity below larynx. Schwenk^67^. (ordered).

590. Tongue tip notching, as percentage of tongue length: (0) no notch; (1) less than 10%; (2) 10–20%; (3) 20–40%; (4) more than 45%. Schwenk^67^. (ordered).

591. Tongue papilla crenellated: (0) continuous smooth distal edges of papilla; (1) crenellated distal edge. Schwenk^67^.

592. Tongue papilla shape: (0) long, filamentous, and densely packed papilla; (1) shorter, larger, somewhat compressed and tipped caudally (scale-like papilla); (2) deeply imbricate flat scales. Schwenk^67^. (ordered).

593. Hind-tongue epithelium: (0) discrete papilla (filamentous or scale-like); (1) transverse plicae confined to lateral margins of posterior lobes; (2) transverse plicae extend across hindtongue; (3) and into the anterior half of the tongue. Schwenk^67^. (ordered).

594. Infralingual folds: (0) absent; (1) present. Harris^74^.

595. Papilla on ventrolateral margins of entire tongue: (0) papillose; (1) plicate (transverse scale rows). (Schwenk^67^). Gauthier *et al*.^4^.

596. Tongue papilla arrangement: (0) not in oblique rows; (1) arranged in regular oblique rows. Schwenk^67^.

597. Hypoglossal muscle: (0) paired and smooth ventrally; (1) multiple, and with fine transverse grooves beneath each muscle bundle. Schwenk^67^.

598. Facial tongue wiping (tongue acts as an accessory eyelid): (0) absent; (1) present. Greer^75^.

599. Foretongue surface: (0) papillose; (1) smooth. Schwenk^67^.

600. Foretongue filamentous epithelium anterior extent: (0) extends to tongue tip as long filaments; (1) those overlying chemosensory part of tongue are depressed to varying degree. Schwenk^67^.

601. Arrowhead tongue tip: (0) tongue lateral margins continuous at tip; (1) notched just behind tip. Schwenk^67^.

602. Tongue width across posterior notch/maximum tongue length: (0) 50–60%; (1) 40–44%; (2) 30–35%; (3) 22–25%; (4) less than 12%. Schwenk^67^ (ordered).

603. Hind tongue papilla: (0) not sharply pointed; (1) sharply pointed. Schwenk^67^.

604. Prey prehension: (0) crickets (or larger animals) taken primarily with tongue; (1) primarily with jaws. Schwenk^67^.

605. Amniote penis: (0) absent; (1) hemipenis present. Gauthier, Estes and de Queiroz^10^.

606. Hemipenis mineralizations: (0) absent; (1) comb-like; (2) sleeve-like; (3) spine-like. Gauthier *et al*.^4^.

607. Rectus abdominis muscles: (0) not attached to belly skin; (1) attached to hinges between ventral transverse scale rows. Bhullar^76^.

608. Ulnar nerve pathway: (0) superficial to limb muscles; (1) deep to limb muscles. Jullien & Renous-Lécuru (1972).

609. Dorsal shank muscle innervation: (0) peroneal nerve; (1) interosseous nerve. Jullien & Renous-Lécuru^77^.

610. Ovipary vs. ovovivipary/vivipary: (0) ovipary; (1) ovovivipary to vivipary. Gauthier *et al*.^4^.

# External morphology (Reeder *et al*.^78^)

**Nasal region**

1 (611). Enlarged rostral scale: (0) absent, undifferentiated, (1) present.

2 (612). Rostral scale: (0) not greatly expanded and dominating anterior portion of head, (1) expanded dorsally (onto top of skull), posteriorly, and laterally, dominating anterior portion of head.

3 (613). Posterior split in rostral scale: (0) absent, (1) present (division along posterior margin of scale near midline).

4 (614). External narial opening: (0) enclosed entirely within nasal scale, (1) at edge of nasal scale.

5 (615). External narial opening: (0) separated from rostral, (1) partially in rostral.

6 (616). External narial opening: (0) separated from supralabial, (1) partially in supralabial.

7 (617). Nasal-rostral contact: (0) absent, separated by scales, (1) present. When the narial opening is between scales rather than entirely within a scale, state 1 is used when one of these scales bordering the nasal contacts the rostral.

8 (618). Contact between nasal and supralabial scales: (0) absent (separated by other scales), (1) present. When the narial opening is between scales rather than entirely within a scale, state 1 is used when one of these scales bordering the nasal contacts the supralabial.

*9 (619). Minimum number of scales separating the nasal scales medially: (0) none (nasal scales contact medially), (1) one scale, (2) two, (3) three, (4) four or more scale rows. Previously used phylogenetically (in modified form) by Wiens^79^ (his char. 22). In species that varied intraspecifically, the mean number was used. Although this character ranges up to much higher scale counts, the states were truncated to allow for the five ordered states possible in current implementations of Bayesian analysis. If the nasal opening is surrounded by two or more scales, the count is from the medial-most scale containing the nasal opening, but does not include that scale. The rostral is not counted. Ordered.

**Eye region**

10 (620). Dorsal cephalic scales: (0) relatively small; (1) enlarged (larger than adjacent neck scales).

11 (621). Dorsal cephalic scales: (0) smooth, (1) some or all keeled.

12 (622). Head scales vertically elongate: (0) no, (1) yes, including scales associated with eye and nostril (extending almost from mouth to top of skull).

13 (623). Enlarged frontal scale between orbits: (0) absent, (1) present. Previously used phylogenetically by Wiens^79^ (his char. 18).

14 (624). Median contact of frontoparietals: (0) present, (1) absent, frontoparietals reduced and separated by contact of frontal and interparietal. Scored as unknown in taxa lacking frontals, frontoparietals, and an interparietal.

15 (625). Circumorbital scales (distinct semicircular row of scales bordering orbital region): (0) absent, (1) present.

16 (626). Enlarged supraoculars: (0) absent, (1) present (scales over eye larger than surrounding scales).

17 (627). Supraoculars: (0) two or more rows of scales, (1) single row of enlarged scales between superciliary and frontal. Taxa with multiple rows of scales in this region were given state 0, regardless of whether or not they had well-differentiated supraoculars. Previously used phylogenetically by Wiens^79^ (his char. 25).

18 (628). Single enlarged supraocular scale row between orbit laterally and frontal medially: (0) no, (1) yes, supraciliary scales absent.

19 (629). Median contact of enlarged supraoculars: (0) absent, (1) present.

20 (630). Supercilary scales: (0) non-overlapping, (1) overlapping. Used previously in modified form by Etheridge and de Queiroz^30^ (their char. 46) and subsequent authors.

21 (631). Supralabial scales: (0) not entering orbit, (1) entering orbit. In most squamates examined, one or more enlarged scales (suboculars) separate the supralabials from the rim of the orbital opening. In others, supralabials extend to the orbital opening.

22 (632). Enlarged subocular scale (elongate scale ventral to orbit): (0) absent (or series of smaller scales), (1) present (single scale larger than surrounding scales). In many taxa, there is a single scale below the orbit that is also part of the labial series bordering the upper jaw (see character 21). In these cases, it is possible that the subocular has entered the labial series or that the labial has shifed into the subocular position, and these taxa were coded as unknown for the presence or absence of a subocular. Used previously phylogenetically in modified form by Etheridge and de Queiroz^30^ (their char. 47) and subsequent authors.

23 (633). Subocular-supralabial contact: (0) absent, row of one or more scales between scales above lip (supralabials) and below the orbit (suborbitals), (1) present. Taxa lacking any subocular scales below the eye (e.g., many snakes) were coded as inapplicable.

24 (634). Enlarged interparietal scale: (0) absent, (1) present (scale distinctly larger than surrounding head scales present over parietal region of skull). Used in modified form by Etheridge and de Queiroz^30^ (their char. 45) and subsequent authors.

25 (635). Temporal region covered almost exclusively by pair of enlarged temporal scales: (0) no, (1) yes. Derived state is mostly in advanced snakes.

26 (636). Heat-sensing pit in loreal region: (0) absent, (1) present.

**Mouth**

27 (637). Mouth: (0) large, extending posterior to eye, (1) greatly reduced, posterior terminus at or anterior to level of eye.

28 (638). Groove allowing tongue to be extruded when mouth is closed: (0) absent, (1) groove present in rostral (sometimes in mental also, but too difficult to score consistently as a separate state). The derived state is found in snakes.

**Eye**

29 (639). Eye: (0) visible externally, (1) not visible externally. Previously used phylogenetically (in modified form) by Kearney^38^ (her char. 10). Derived state is found only in *Rhineura*.

30 (640). Eye, surrounded by single scale: (0) no, multiple scales, (1) single scale. Previously used phylogenetically (in modified form) by Lee and Scanlon^31^ (their char. 253).

31 (641). Fleshy eyelids: (0) present; (1) absent. Previously used phylogenetically (in modified form) by Lee and Scanlon^31^ (their char. 253).

32 (642). Lower eyelid scales: (0) homogeneous, (1) some enlarged scales.

33 (643). Transparent spectacle in lower eyelid scales: (0) absent, (1) present.

34 (644). Thick granular eyelid covering eye except for small opening for pupil (chamaeleon condition): (0) absent; (1) present. Used previously phylogenetically by Estes *et al.*^20^ (p. 199).

**Ear**

35 (645). External ear opening (0) present, (1) absent. Previously used phylogenetically by Estes *et al*.^20^ (48).

36 (646). Tympanum: (0) large and superficial; (1) small and inset. Used previously phylogenetically by Estes *et al*.^20^.

37 (647). External ear opening: (0) entirely or mostly uncovered, (1) partially covered by fleshy lobule, (2) partially covered by scales only, no fleshy lobule. Unordered.

**Throat/chin**

38 (648). Complex dewlap apparatus (gular fan), in males: (0) absent; (1) present, extensive flap of skin on throat, usually with modified scales.

39 (649). Mental scale: (0) entire, (1) with a partial split at the posterior end. Previously noted by Frost and Etheridge^43^ (from E. Williams, pers. comm.).

40 (650). Elongate postmentals: (0) absent, (1) present.

41 (651). Postmentals (scales immediately posterior to mental): (0) two (enlarged scales sometimes separated by additional scales) or more scales immediately posterior to mental, (1) one, single unpaired scale immediately posterior to mental.

42 (652). Enlarged scales medial to infralabials: (0) present, (1) absent, scales in chin more-or-less homogeneous.

*43 (653). Infralabials, anteriormost pair(s): (0) separate medially, (1) one row contacting medially posterior to mental, (2) two rows contacting medially. It is possible that the medially contacting infralabials actually represent postmentals that have shifted their position. Ordered.

44 (654). Infralabial and supralabial scales: (0) not greatly expanded, (1) greatly expanded, with single pair of sublabials covering most of chin region (i.e., dibamids).

45 (655). Mental groove: (0) absent, (1) present, a distinct infolding of skin and scales along the anterior midline of the chin region.

*46 (656). Gular fold: (0) present, complete, (1) incomplete; (2) absent. Ordered. Previously used phylogenetically by Etheridge and de Queiroz^30^ (their char. 43) and subsequent authors.

**Dorsal body region**

47 (657). Body scales: (0) not arranged in non-overlapping rings; (1) annulated, arranged in non-overlapping rows. Previously used phylogenetically by Kearney^38^ (her char. 3).

48 (658). *Varanus*-type scale on dorsum, with small scale surrounded by tiny granular scales: (0) absent, (1) present.

49 (659). Dorsal scales: (0) homogeneous, lacking enlarged scales, (1) heterogeneous, with enlarged scales separated by smaller scales, either in rows or scattered.

50 (660). Dorsal body scales: (0) small, granular, non-overlapping, (1) mucronate (pointed, overlapping), (2) cycloid (rounded, overlapping), (3) rectangular. Unordered. Expanded from char. 148 of Estes *et al*.^20^.

51 (661). Dorsal body scales: (0) all smooth; (1) some or all keeled.

52 (662). Dorsals, with longitudinal ridges in addition to mid-dorsal keels: (0) absent (including species lacking keels), (1) present

53 (663). Enlarged middorsal scale row: (0) middorsal scales same size as surrounding scales; (1) middorsal scales enlarged relative to surrounding scales in both sexes; (2) sexually dimorphic, mid-dorsals enlarged in males. Unordered. Expanded and modified from char. 146 of Estes *et al.*^20^.

54 (664). Nuchal crest of enlarged scales: (0) absent, (1) present in both sexes; (2) sexually dimorphic, mid-dorsals enlarged in males. Unordered. Has similar distribution to that of mid-dorsal scale row, but clearly not identical.

55 (665). Lateral body scales: (0) granular, non-overlapping, (1) imbricate overlapping. Although this may seem redundant with the condition of the dorsal scales, several taxa have enlarged and imbricate dorsal scales but granular lateral scales.

56 (666). Lateral fold along side of body: (0) absent, (1) present (often associated with row of granular scales in species with larger scales). Previously used phylogenetically in squamates by Estes *et al*.^20^.

**Venter**

57 (667). Gastrosteges (enlarged mid-ventral scale row in snakes): (0) absent, multiple scale rows; (1) present, single mid-ventral scale row distinctly larger than surrounding scale rows. Previously used phylogenetically (in modified form) by Lee and Scanlon^31^ (their char. 255).

58 (668). Ventrals: (0) same size as adjacent laterals, (1) at least some distinctly larger than adjacent laterals.

59 (669). Ventral body scales: (0) mostly small and granular; (1) large and flat. Note that this character is independent of the preceding character, because scales can be large and flat and still be the same size as the adjacent laterals.

60 (670). Ventral scales: (0) all smooth, (1) some or all keeled.

61 (671). Ventrals, posterior edge: (0) cycloid, mucronate, or otherwise rounded, (1) square (posterior margin more-or-less straight). Some snakes with gastrosteges have variation that approaches the condition in state 1, but were coded as 0.

62 (672). Femoral pores: (0) absent; (1) present in both sexes, (2) sexually dimorphic. Unordered. In some cases, femoral and preanal pores may be difficult to distinguish (e.g. *Gekko gecko, Takydromus ocellatus*). In general, pores on the legs were considered femoral pores, and pores between the legs were considered preanals, but pores that extended from the legs onto the preanal region were considered femorals. Taxa lacking hindlimbs were coded as unknown. Previously used phylogenetically by Estes *et al*.^20^ (but noted by Camp^80^), their character 144.

63 (673). Preanal pores: (0) absent; (1) present. Previously used phylogenetically by Estes *et al.*^20^, their character 144.

**Tail**

64 (674). Tail tip: (0) pointed, tapered, (1) blunt and rounded

65 (675). Small sharply pointed spike at posterior tail tip: (0) absent, (1) present.

*66 (676). Tail shape (cross-section): (0) dorsoventrally flattened (depressed), (1) rounded, (2) laterally compressed. Ordered.

67 (677). Caudal scales: (0) approximately same size as dorsal body scales: (1) distinctly larger than dorsal body scales

68 (678). Caudals (dorsal): (0) smooth, (1) mostly keeled.

69 (679). Caudal scales: (0) not arranged in non-overlapping vertical rows; (1) annulated, arranged in non-overlapping vertical rows.

70 (680). Enlarged preanal scales: (0) absent, (1) present (preanals enlarged relative to adjacent ventrals).

71 (681). Anal plate: (0) two or more scales, (1) single plate. Species without a distinct anal plate but with multiple scales in the pre-anal region were coded as having state 0. Previously used phylogenetically by Lee and Scanlon^31^ (their char. 257).

72 (682). Enlarged postanal scales (males): (0) absent, (1) present (some scales posterior to vent greatly enlarged relative to adjacent scales).

73 (683). Lateral postanal tubercles: (0) absent, (1) present (enlarged scale or scales on medial surface of anterior end of tail).

*74 (684). Subcaudals (scales on ventral mid-line of tail): (0) divided, two or more scale rows, (1) some single, some divided, (2) all single. Ordered. Previously used phylogenetically (in modified form) by Lee and Scanlon^31^ (their char. 256).

**Limbs**

75 (685). External hindlimb: (0) present, (1) absent in both sexes, (2) sexually dimorphic, present in males, absent in females. Unordered. Taxa in which hindlimbs were observed in all individuals but the sex was uncertain were coded as having state 0.

76 (686). External hindlimbs: (0) cylindrical, (1) flattened and flaplike. Taxa having the fully-developed limbs and those having only a claw were both coded as having state 0.

77 (687). Expanded toe pads: (0) absent, (1) present. Previously used phylogenetically by Etheridge and de Queiroz^30^ (their char. 40) and subsequent authors.

78 (688). Number of rows of subdigital scales along mid-ventral surface of digits (manus): (0) one, (1) two or more (some or all scales). Previously used phylogenetically by Estes et al. (1988) and Etheridge and de Queiroz^30^.

79 (689). Keels on subdigital scales (hand, longest finger): (0) absent, (1) present. Used previously phylogenetically by Etheridge and de Queiroz^30^ (their char. 39).

80 (690). Digits connected in groups of 2 and 3 by skin (chameleon “mittens”): (0) no, (1) yes. Previously used phylogenetically (in modified form) by Estes *et al.*^20^ (p. 198).

81 (691). Claws: (0) not covered by sheath of scales, (1) entirely or mostly covered by sheath of scales.

**4. Results obtained using different data set and character ordering**

For the results, see Table S1.

**5. Autapomorphies of *Retinosaurus hkamtiensis* (characters that might change during ontogeny are indicated with an asterisk). The asterisked characters were changed to ? in the main analysis**

Char. 5: 0 --> 1

Char. 18: 1 --> 0

Char. 22: 1 --> 0

Char. 38: 0 --> 1*

Char. 56: 1 --> 2

Char. 76: 2 --> 0*

Char. 108: 1 --> 0*

Char. 116: 1 --> 2*

Char. 167: 2 --> 0*

Char. 185: 2 --> 4

Char. 188: 3 --> 4

Char. 217: 1 --> 0*

Char. 258: 3--> 2

Char. 281: 0 --> 1

Char. 286: 0 --> 1

Char. 297: 1 --> 0

Char. 390: 2 --> 1

Char. 394: 0 --> 1

Char. 399: 1 --> 0*

Char. 493: 1 --> 0*

Char. 500: 2 --> 0*

Char. 533: 1 --> 0 *

Char. 534: 1 --> 0*

Char. 535: 1 --> 0*

Char. 615: 0 --> 1

Char. 649: 0 --> 1

Char. 660: 0 --> 3

**6. The locality**

The amber, in which the holotype of *Retinosaurus hkamtiensis* (GRS 29689) was trapped, was recovered from the Hkamti District at Patabum (Sagaing State). It is located in close proximity of the Jade mines, 100 km to the southwest of the Hukawng Basin in the northern Myanmar Central Basin (see Supplementary Fig. 6; see also Sun et al.^81^). Amber from this mine at the Hkamti District has been dated to the Albian, ca. 110 million years ago (Ma), using zircon U-Pb isotopes^82^.


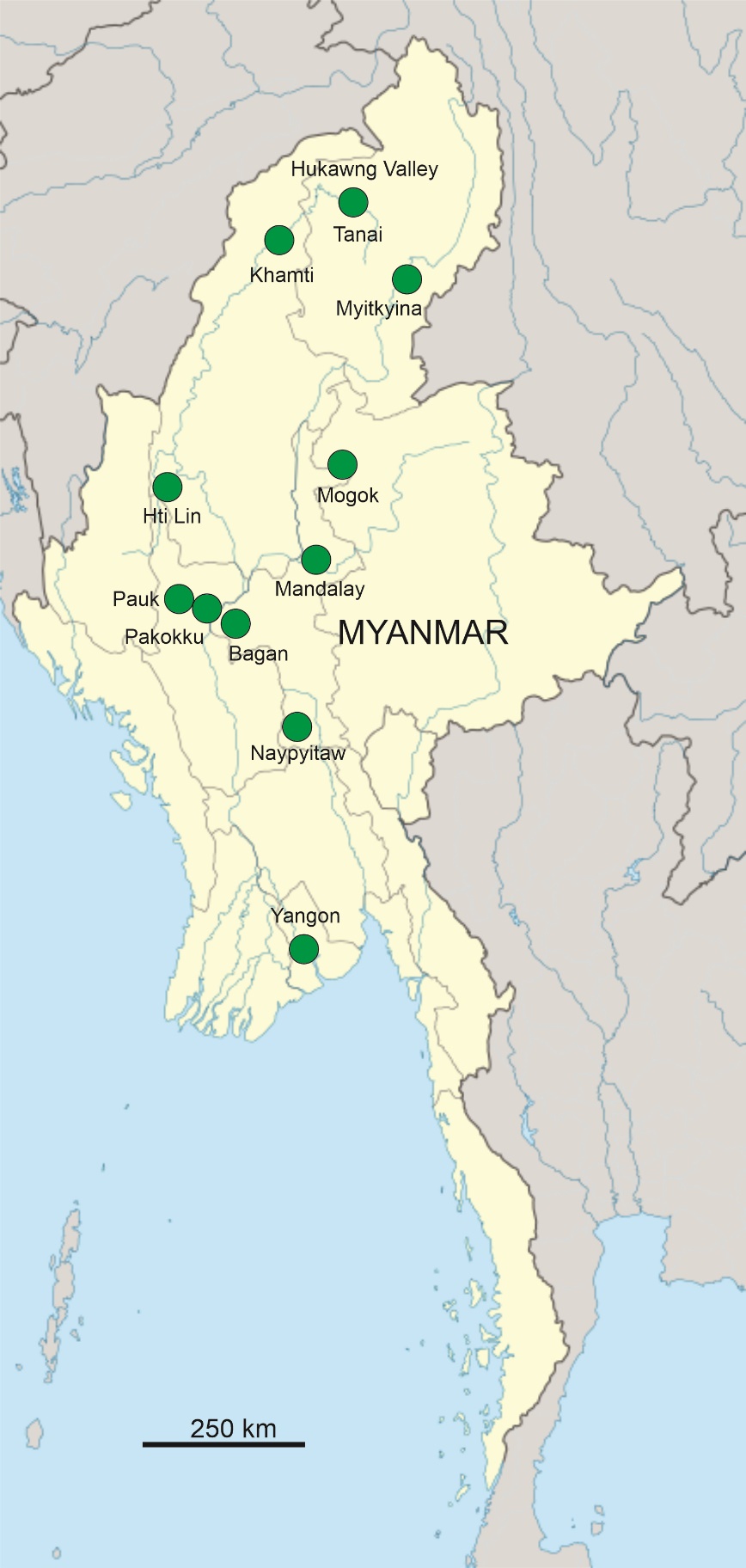


**Supplementary Fig. 6.** The significant amber-mining areas in Myanmar (the map has been modified from the naturalearthdata map which is in the GNU Free Documentation License).

**7. References**

1. Romer, A. S. Osteology of the Reptiles. (University of Chicago Press; 1–772, 1956).

2. Evans, S. E. The skull of lizards and Tuatara. (Eds. Gans, C., Gaunt, A. S. & Adler, K.) Biology of the Reptilia, 20 The Skull of Lepidosauria (Ithaca, New York. Society for the Study of Amphibians and Reptiles, Contributions to Herpetology, 23, 1–347, 2008).

3. Čerňanský, A., Smith, K. T., Klembara, J. Variation in the position of the jugal medial ridge among lizards (Reptilia: Squamata): its functional and taxonomic significance. *Anat. Rec.* **297**, 2262–2272 (2014).

4. Gauthier, J., Kearney, M., Maisano, J. A., Rieppel, O. & Behlke, A. Assembling the squamate tree of life: Perspectives from the phenotype and the fossil record. Bull., Peabody Mus. Nat. Hist. 53, 3–308 (2012).

5. Klembara, J. New finds of anguines (Squamata, Anguidae) from the early Miocene of Northwest Bohemia (Czech Republic). *PalZ.* **89**, 171–195 (2015).

6. Boistel, R. *et al.* Shake, rattle and roll: the bony labyrinth and aerial descent in squamates. *Integr. Comp. Biol.* **51**, 957–968 (2011).

7. Dickson, B. V., Sherratt, E., Losos, J. B. & Pierce, S. E. Semicircular canals in *Anolis* lizards: ecomorphological convergence and ecomorph affinities of fossil species. *R. Soc. Open Sci.* https://doi.org/10.1098/rsos.170058 (2017).

8. Palci, A., Hutchinson, M. N., Caldwell, M. W., Lee, M. S. Y. The morphology of the inner ear of squamate reptiles and its bearing on the origin of snakes. *R. Soc. Open Sci.* https://doi.org/10.1098/rsos.170685 (2017).

9. Daza, J.D., Bauer, A.M., Stanley, E.L., Bolet, A., Dickson, B. & Losos, J. B. An enigmatic miniaturized and attenuate whole lizard from the mid-Cretaceous amber of Myanmar. Breviora 563, 1–18 (2018).

10. Gauthier, J. A., Estes, R. & De Queiroz, K. A phylogenetic analysis of Lepidosauromorpha. (Eds. Estes, R. & Pregill, G.) Phylogenetic Relationships of the Lizard Families (Stanford, Stanford University Press, 15–98, 1988).

11. Rieppel, O.. The sound transmitting apparatus of primitive snakes and its phylogenetic significance. *Zoomorphology* **96**, 45–62 (1980a).

12. Moody, S. Phylogenetic and historical biogeographical relationships of the genera in the family Agamidae (Reptilia: Lacertilia). (Dissertation, Ann Arbor: University of

Michigan, ProQuest Dissertations and Theses, 1–373, 1980).

13. Gauthier, J. A. Fossil Xenosauridae and Anguidae from the Lower Eocene Wasatch Formation, southcentral Wyoming, and a revision of the Anguioidea. *University of*

*Wyoming Contributions to Geology* **21**, 7–54 (1982).

14. Lee, M. S. Y. Convergent evolution and character correlation in burrowing reptiles: towards a resolution of squamate relationships. *Biol. J. Linn. Soc.* **65**, 369–453 (1998).

15. Cundall, D. Feeding behaviour in *Cylindrophis* and its bearing on the evolution of alethinophidian snakes. *J. Zool.* **237**, 353–376 (1995).

16. Kearney, M. The phylogenetic position of *Sineoamphisbaena hexatabularis* reexamined. *J. Vertebr. Paleontol.* **23**, 394–403 (2003a).

17. Cundall, D., Wallach, V. & Rossman, D. A. The systematic relationships of the snake genus *Anomochilus*. *Zool. J. Linn. Soc.***109**, 275–299 (1993).

18. Kluge, A. G.. *Calabaria* and the phylogeny of erycine snakes. *Zool. J. Linn. Soc.* **107**, 293–351 (1993a).

19. Pregill, G. K., Gauthier, J. A. & Greene, H. W. The evolution of helodermatid squamates, with description of a new taxon and an overview of Varanoidea. *Trans. San Diego Soc. Nat. Hist.* 2**1**, 167–202 (1986).

20. Estes, R., de Queiroz, K. & Gauthier, J. A. Phylogenetic relationships within Squamata. (Eds. Estes, R. & Pregill, G.) Phylogenetic Relationships of the Lizard Families: Essays Commemorating Charles L. Camp (Stanford University Press, Stanford, California, 119–281, 1988).

21. Bell, G. L. A phylogenetic revision of North American and Adriatic Mosasauroidea. (Eds. Callaway, J. & Nicholls, E.) Ancient Marine Reptiles (San Diego, Academic Press, 293–332, 1997).

22. Rieppel, O. The naso-frontal joint in snakes as revealed by highresolution X-ray computed tomography of intact and complete skulls. *Zool. Anz.* **246**, 177–191 (2007).

23. Crother, B.I., Miyamoto, M. M. & Presch, W. F. Phylogeny and biogeography of the lizard family Xantusiidae. *Syst. Zool.* **35**, 37–45 (1986).

24. Smith, K. T. Eocene lizards of the clade *Geiseltaliellus* from Messel and Geiseltal, Germany, and the early radiation of Iguanidae (Reptilia: Squamata). *Bull., Peabody Mus. Nat. Hist.* **50**, 219–306 (2009a).

25. Kluge, A. G. *Aspidites* and the phylogeny of pythonine snakes. (Australian Museum Sydney South, NSW, Australia, *Records of the Australian Museum Suppl*. **19**, 1–77, 1993b).

26. Smith, K. T. A new lizard assemblage from the earliest Eocene (zone Wa0) of the Bighorn Basin, Wyoming, U.S.A.: biogeography during the warmest interval of the Cenozoic. *J. Syst. Palaeontol.* **7**, 299–358 (2009b).

27. Wu, X. C., Brinkman, D. B. & Russell, A. P. *Sineoamphisbaena hexatabularis*, an amphisbaenian (Diapsida: Squamata) from the Upper Cretaceous redbeds at Bayan Mandahu (Inner Mongolia, People’s Republic of China), and comments on the phylogenetic relationships of the Amphisbaenia. *Can. J. Earth Sci.* **33**, 541–577. 1996.

28. Arnold, E. N. Cranial kinesis in lizards; variations, uses, and origins. (Eds. Eggleton, P. & Vane-Wright, R. I.) Phylogeny and Ecology (London, Academic Press, 323–357, 1998).

29. Lee, M. S. Y. & Caldwell, M. W. Anatomy and relationships of *Pachyrhachis problematicus*, a primitive snake with hindlimbs. *Philos. Trans. R. Soc. B* **353**, 1521–1552 (1998).

30. Etheridge, R. & de Queiroz K. A phylogeny of Iguanidae. (Eds. Estes, R. & Pregill, G.) Phylogenetic Relationships of the Lizard Families (Stanford, Stanford University Press, 283–367, 1988).

31. Lee, M. S. Y. & Scanlon, J. D. Snake phylogeny based on osteology, soft anatomy, and ecology. *Biol. Rev.* **77**, 333–401 (2002).

32. Lang, M. Generic relationships within Cordyliformes (Reptilia: Squamata). *Bull. Inst. r. sci. nat. Belg., Entomol. biol.* **61**, 121–188 (1991).

33. Tchernov, E., Rieppel, O., Zaher, H., Polcyn, M. J. & Jacobs, L. J. A new fossil snake with limbs. *Science* **287**, 2010–2012 (2000).

34. Rieppel, O., Gauthier, J. A. & Maisano, J. Comparative morphology of the dermal palate in squamate reptiles, with comments on phylogenetic implications. *Zool. J. Linn. Soc.* **152**, 131–152 (2008).

35. Kardong, K. V. Kinesis of the jaw apparatus during the strike in the cottonmouth snake, *Agkistrodon piscivorus.* *Forma et Functio* **7**, 327–354 (1994).

36. Kluge, A. G. Boine snake phylogeny and research cycles. (Ann Arbor. Museum of Zoology, University of Michigan. pp. 58 *Miscellaneous Publications* 178, 1991).

37. Cundall, D. & Irish, F. J. The function of the intramaxillary joint in the Round Island boa, *Casarea dussumieri*. *J. Zool.* **217**, 569–598 (1989).

38. Kearney, M. Systematics of the Amphisbaenia (Lepidosauria: Squamata) based on morphological evidence from Recent and fossil forms. *Herpetol. Monogr.* **17**, 1–74 (2003b).

39. Lee, M. S. Y. *et al.* Miocene skinks and geckos reveal long-term conservatism of New Zealand’s lizard fauna. *Biol. Lett.* **5**, 833–837 (2009).

40. Gauthier, J. A. A cladistic analysis of the higher systematic categories of the Diapsida. (dissertation, Berkeley, University of California, 1–564, 1984).

41. Borsuk-Białynicka, M. & Moody, S. M. Priscagaminae, a new subfamily of the Agamidae (Sauria) from the late Cretaceous of the Gobi Desert. *Acta Palaeontol. Pol.* **29**, 51–81 (1984).

42. Benton, M. J. The relationships and early evolution of the Diapsida. (Eds. Ferguson, M. W. J.) The Structure, Development and Evolution of Reptiles (London, Cambridge University Press, Symposia of the Zoological Society of London 52, 575–596, 1984).

43. Frost, D. & Etheridge, R. A phylogenetic analysis and taxonomy of iguanian lizards (Reptilia: Squamata). *Misc. publ. Univ. Kans. Mus. Nat. Hist.* **81**, 1–65 (1989).

44. De Queiroz, K. Phylogenetic systematics of iguanine lizards: a comparative osteological study. *Univ. Calif. publ. zool.* **118**, 1–216 (1987).

45. Rieppel, O. The phylogenetic relationships within the Chamaeleonidae, with comments on some aspects of cladistic analysis. *Zool. J. Linn. Soc.* **89**, 41–62 (1987).

46. Rieppel, O. The evolution of the ophidian feeding system. *Zool. Jb. Anat.* **103**, 551–564. (1980b).

47. DeBraga, M. & Carroll, R. L. The origin of mosasaurs as a model of macroevolutionary patterns and processes. *Evol. Biol.* **27**, 245–322 (1993).

48. Caldwell, M. W. Squamate phylogeny and the relationships of snakes and mosasauroids. *Zool. J. Linn. Soc.* **125**, 115–147 (1999).

49. Lee, M. S. Y. The phylogeny of varanoid lizards and the affinities of snakes. *Philos. Trans. R. Soc. B* **352**, 53–91 (1997a).

50. Rieppel, O., Kluge, A. G. & Zaher, H. Testing the phylogenetic relationships of the Pleistocene snake *Wonambi naracoortensis* Smith. *J. Vertebr. Paleontol.* **22**, 812–829 (2002).

51. Mahler, L. & Kearney, M. The palatal dentition in squamate reptiles: morphology, development, attachment, and replacement. *Fieldiana* **1540**, 1–61 (2006).

52. Rieppel, O. The skull and jaw adductor musculature in some burrowing scincomorph lizards of the genera *Acontias*, *Typhlosaurus* and *Feylinia.* *J.Zool.* **195**, 493–528 (1981).

53. Presch, W. Cladistic relationships within Scincomorpha. (Eds. Estes, R. & Pregill, G.) Phylogenetic Relationships of the Lizard Families (Stanford: Stanford University Press, 471–492, 1988).

54. Rieppel, O. Miniaturization of the lizard skull: its functional and evolutionary implications. (Eds. Ferguson, M. W. J.) The Structure, Development and Evolution of Reptiles (London: Academic Press., Zoological Society of London Symposia 52, 503–520, 1984a).

55. Rieppel, O. The cranial morphology of the fossorial lizard genus *Dibamus* with a consideration of its phylogenetic relationships. *J. Zool.* **204**, 289–327 (1984b).

56. Rieppel, O. The homology of the laterosphenoid bone in snakes. *Herpetologica* **32**, 426–429 (1976).

57. Rieppel, O. The braincase of *Typhlops* and *Leptotyphlops* (Reptilia, Serpentes). *Zool. J. Linn. Soc.* **65**, 161–176 (1979a).

58. Conrad, J. L. & Norell, M. A. The braincases of two glyptosaurines (Anguidae, Squamata) and anguid phylogeny. *Am. Mus. Novit* **3613**, 1–24 (2008).

59. Rieppel, O. The classification of primitive snakes and the testability of phylogenetic theories. *Biol. Zent.* **98**, 537–552 (1979b).

60. Rieppel, O. The recessus scalae tympani and its bearing on the classification of reptiles. *J. Herpetol.* **19**, 373–384 (1985).

61. Conrad, J. L. Phylogeny and systematics of Squamata (Reptilia) based on morphology. *Bull. Am. Mus. Nat. Hist.* **310**, 1–182 (2008).

62. Gao, K.-Q. & Norell, M. A. Taxonomic composition and systematics of Late Cretaceous lizard assemblages from Ukhaa Tolgod and adjacent localities, Mongolian Gobi Desert. *Bull. Am. Mus. Nat. Hist.* **249**, 1–118 (2000).

63. Oelrich, T. M. Anatomy of the head of *Ctenosaura pectinata* (Iguanidae). Ann Arbor, MI: Museum of Zoology. *Miscellaneous Publications of the Museum of Zoology of the University of Michigan* **94**, 1–122. (1956).

64. Lee, M. S. Y. On snake-like dentition in mosasaurian lizards. *J. Nat. Hist.* **31**, 303–314 (1997b).

65. Rieppel, O. & Zaher, H. The intramandibular joint in squamates, and the phylogenetic relationships of the fossil snake *Pachyrhachis problematicus* Haas. *Fieldiana (Geology)* **1507**, 1–60 (2000).

66. Evans, S. E., Prasad, G. V. R. & Manthas, B. K. Fossil lizards from the Jurassic Kota Formation of India. *J. Vertebr. Paleontol.* **22**, 299–312 (2002).

67. Schwenk, K. Comparative morphology of the lepidosaur tongue and its relevance to squamate phylogeny. (Eds. Estes, R. & Pregill, G.) Phylogenetic Relationships of the Lizard Families (Stanford: Stanford University Press, 569–598, 1988).

68. McGuire, J. Phylogenetic systematics of crotaphytid lizards (Reptilia: Iguania: Crotaphytidae). *Bulletin of Carnegie Museum of Natural History* **32**, 1–143 (1996).

69. Kluge, A. G. Cladistic relationships in the Gekkonoidea (Squamata, Sauria). (Ann Arbor: Museum of Zoology, University of Michigan. 54 pp. *Miscellaneous Publications* 173, 1987).

70. Rieppel, O. & Head, J. New specimens of the fossil snake genus *Eupodophis* Rage and Escuillié, from the mid-Cretaceous of Lebanon. *Mem. Soc. ital. sci. nat. Mus. civ. stor. nat. Milano* **32**, 1–26 (2004).

71. Grismer, L. L. Phylogeny, taxonomy, classification, and biogeography of eublepharid geckos. (Eds. Estes, R. & Pregill, G.) Phylogenetic Relationships of the Lizard Families (Stanford: Stanford University Press, 369–469, 1988).

72. Lee, M. S. Y. Soft anatomy, diffuse homoplasy, and the relationships of lizards and snakes. *Zool. Scr.* **29**, 101–130 (2000).

73. Hallermann, J. The ethmoidal region of *Dibamus taylori* (Squamata: Dibamidae), with a phylogenetic hypothesis on dibamid relationships within Squamata. *Zool. J. Linn. Soc.* **122**, 385–426 (1998).

74. Harris, D. Infralingual plicae: support for Boulenger’s Teiidae (Sauria). *Copeia* **1985**, 560–565 (1985).

75. Greer, A. E. Facial tongue-wiping in xantusiid lizards: its systematic implications. *J. Herpetol.* **19**, 174–175 (1985).

76. Bhullar, B. A. S. A reevaluation of the unusual abdominal musculature of squamate reptiles (Reptilia: Squamata). *Anat. Rec.* **292**, 1154–1161 (2009).

77. Jullien, R. & Renous-Lécuru, S. Variations du rajet du nerf ulnaire (ulnaris) et de l’innervation des muscles dorsaux de la jambe chez les lacertiliens (reptiles, squamates): valeur systématique et application phylogénétique. *Bull. Mus. Natl. Hist. Nat.* ***2*3**, 207–245 (1972).

78. Reeder, T. W. *et al.* Integrated analyses resolve conflicts over squamate reptile phylogeny and reveal unexpected placements for fossil taxa. PLoS ONE 10, e0118199 (2015).

79. Wiens, J. J. Polymorphism in systematics and comparative biology. *Ann Rev Ecol Syst.* **30**, 327–362 (1999).

80. Camp, C.L. Classification of the lizards. Bull. Am. Mus. Nat. Hist. 48, 289–481 (1923).

81. Sun, T. T. *et al.* Burmese Amber from Hti Lin. J. Gemmol. 34, 606–615 (2015).

82. Xing, L. & Qiu L. Zircon UePb age constraints on the mid-Cretaceous Hkamti amber biota in northern Myanmar. Palaeogeogr. Palaeoclimatol. Palaeoecol. 558, 109960 (2020).
