# Supplementary material for: A new Early Cretaceous lizard in Myanmar amber with exceptionally preserved integument
Source: Sci Rep. 2022 Jan 31;12:1660. doi: 10.1038/s41598-022-05735-5 (PMC8803969; doi:10.1038/s41598-022-05735-5)
Supplement: Supplementary file 3 — Supplementary Information 3. [file 41598_2022_5735_MOESM3_ESM.docx]

**Table S1.** Results obtained using different data set and character ordering. Trees available in the supplementary file.
